# Biologically Active Metabolites from the Marine Sediment-Derived Fungus *Aspergillus flocculosus*

**DOI:** 10.3390/md17100579

**Published:** 2019-10-11

**Authors:** Anton N. Yurchenko, Phan Thi Hoai Trinh, Elena V. Girich (Ivanets), Olga F. Smetanina, Anton B. Rasin, Roman S. Popov, Sergey A. Dyshlovoy, Gunhild von Amsberg, Ekaterina S. Menchinskaya, Tran Thi Thanh Van, Shamil Sh. Afiyatullov

**Affiliations:** 1 G.B. Elyakov Pacific Institute of Bioorganic Chemistry, Far Eastern Branch of the Russian Academy of Sciences, Prospect 100-letiya Vladivostoka, 159, Vladivostok 690022, Russiasmetof@rambler.ru (O.F.S.); abrus__54@mail.ru (A.B.R.); prs_90@mail.ru (R.S.P.); dyshlovoy@gmail.com (S.A.D.); ekaterinamenchinskaya@gmail.com (E.S.M.); afiyat@piboc.dvo.ru (S.S.A.); 2 Department of Marine Biotechnology, Nhatrang Institute of Technology Research and Application, Vietnam Academy of Science and Technology, 650000 Nha Trang, Vietnam; phanhoaitrinh84@gmail.com (P.T.H.T.); tranthanhvan@nitra.vast.vn (T.T.T.V.); 3 School of Natural Science, Far Eastern Federal University, Sukhanova St., 8, Vladivostok 690000, Russia; 4 Laboratory of Experimental Oncology, Department of Oncology, Hematology and Bone Marrow Transplantation with Section Pneumology, Hubertus Wald-Tumorzentrum, University Medical Center Hamburg-Eppendorf, 20246 Hamburg, Germany; g.von-amsberg@uke.de; 5 Martini-Klinik Prostate Cancer Center, University Hospital Hamburg-Eppendorf, 20246 Hamburg, Germany

**Keywords:** marine-derived fungi, secondary metabolites, polyketides, drimanes, meroterpenoids, cytotoxicity

## Abstract

Four new compounds were isolated from the Vietnamese marine sediment-derived fungus *Aspergillus flocculosus*, one aspyrone-related polyketide aspilactonol G (**2**), one meroterpenoid 12-epi-aspertetranone D (**4**), two drimane derivatives (**7**,**9**), together with five known metabolites (**1**,**3**,**5**,**6**,**8**,**10**). The structures of compounds **1**–**10** were established by NMR and MS techniques. The absolute stereoconfigurations of compounds **1** and **2** were determined by a modified Mosher’s method. The absolute configurations of compounds **4** and **7** were established by a combination of analysis of ROESY data and coupling constants as well as biogenetic considerations. Compounds **7** and **8** exhibited cytotoxic activity toward human prostate cancer 22Rv1, human breast cancer MCF-7, and murine neuroblastoma Neuro-2a cells.

## 1. Introduction

Marine fungi are rich sources of new biologically active compounds [1]. Fungi of the genus *Aspergillus*, section *Circumdati* (*Aspergillus insulicola*, *Aspergillus flocculosus*, *Aspergillus ochraceus*, *Aspergillus ochraceopetaliformis,* and others) [2], are known to produce metabolites belonging to various chemical classes: aspyrone-related pentaketides [3,4], meroterpenoids [5,6], diketopiperazine alkaloids [7], drimane sesquiterpenoids and their nitrobenzoyl derivatives [8,9], steroids, and cerebrosides [10]. Many of them possess antimicrobial [4,10], antiviral [11], cytotoxic [8,11], and neuroprotective [12] activities.

Aspyrone-related pentaketides are polyketide metabolites commonly found in this fungal group [13]. Usually, they are divided into three structural types: linear (aspinonene) [3], δ-lactones (aspyrone) [3], and γ-lactones (iso-aspinonene, aspilactonols) [3,14]. Meroterpenoid metabolites of *Aspergillus,* section *Circumdati* fungi are represented mainly by triketidesesquiterpenoids with rare α-pyrone-contained linear or angular skeleton. To date, only several representatives of this chemical class belonging to the aspertetranones [5] and ochraceopones [6] series were reported. Nitrobenzoyl derivatives of drimane-sesquiterpenoids were initially found in *A. insulicola* species but can also be produced by other related fungi [15]. These compounds are characterized by a small structural diversity with two isomeric backbones (cinnamolide- and confertifolin-based) and various locations of acyl groups. A residue of *p*-nitrobenzoic acid usually can be found at positions 9-OH or 14-OH. Nitrobenzoyl derivatives are relatively unstable compounds that cannot be hydrolyzed to form the corresponding sesquiterpenoids [8]. Acetylation of these compounds with acetic anhydride results in rearrangement and formation of several products [16].

**Figure 1:**
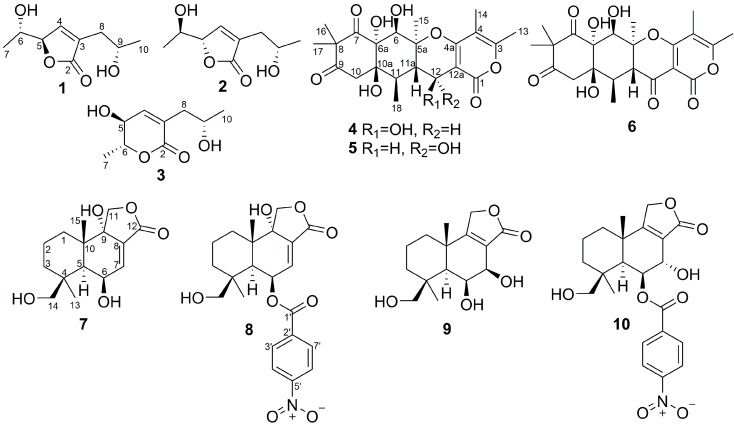
Chemical structures of the isolated compounds **1**–**10**.

Recently, we have started a project focusing on the search for producers of novel bioactive compounds among fungi isolated from various substrates found in the Vietnamese waters of the South China Sea [17,18]. Thus, from a sediment sample collected in Nha Trang Bay, we have isolated a strain of fungus *A. flocculosus*. Recently, we described the new neuroprotective alkaloid mactanamide produced by this strain [12]. Herein, we report the isolation, structure elucidation and cytotoxic activity of four new (**2**,**4**,**7**,**9**) and six known (**1**,**3**,**5**,**6**,**8**,**10**) metabolites produced by the same fungus (Figure 1).

## 2. Results and Discussion

The molecular formula of compound **1** was determined as C_9_H_14_O_4_ by an HRESIMS peak at *m*/*z* 209.0785 [M + Na]^+^, which was supported by the ^13^C NMR spectrum.

A close inspection of the ^1^H and ^13^C NMR data of **1** (Table 1, Figures S1–S3) revealed the presence of two methyls (δ_C_ 23.3, 18.8; δ_H_ 1.31, 1.25), one methylene (δ_C_ 34.9; δ_H_ 2.52, 2.45), three oxygen-bearing *sp*^3^-methines (δ_C_ 84.9, 67.8, 66.2; δ_H_ 4.85, 4.08, 4.05) and one *sp*^2^-methine (δ_C_ 147.4; δ_H_ 7.27). Two remaining signals at δ_C_ 132.8 and 174.2 ppm corresponded to a quaternary *sp*^2^-carbon and a carboxyl carbon, respectively.

**Figure 2:**
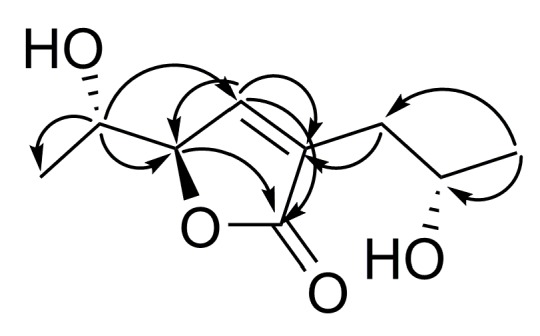
The key HMBC correlations of **1**.

The HMBC correlations (Figure 2 and Figure S6) from H-4 (δ_H_ 7.27) to C-2 (δ_C_ 174.2), C-3 (δ_C_ 132.8), and C-5 (δ_C_ 84.9) and from H-5 (δ_H_ 4.85) to C-2, C-3, and C-4 (δ_C_ 147.4) suggested the presence of a dihydrofuran ring. The structure of the 1-hydroxyethyl side chain and its location at C-5 in **1** was established by COSY correlations of H-6/H-5 and H-7 and HMBC correlations from H-6 (δ_H_ 4.05) to C-4, C-5, and C-7 (δ_C_ 18.8). The data of COSY spectrum (Figure S4) and HMBC correlations from H-10 (δ_H_ 1.25) to C-8 (δ_C_ 34.9), C-9 (δ_C_ 66.2), and from both H_2_-8 (δ_H_ 2.52, 2.45) to C-3, C-4, C-9, and C-10 (δ_C_ 23.3) determined the structure of the 2-hydroxypropyl side chain and its location at C-3.

**Figure 3:**
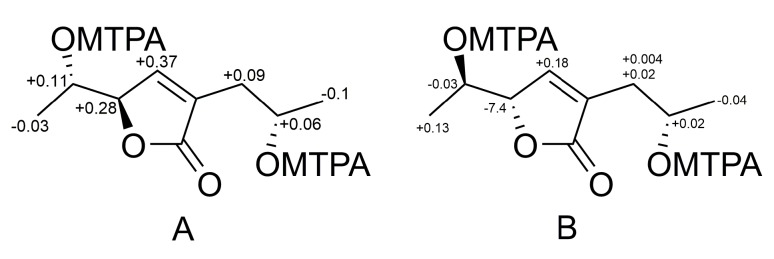
Δδ (δ_S_−δ_R_) values (in ppm) for the MTPA ester of **1** (**A**) and **2** (**B**).

The absolute configuration of the chiral centers C-6 and C-9 of **1** was established using a modified Mosher’s method. Esterification of the C-6 and C-9 hydroxy moieties of **1** with (*R*)- and (*S*)-MTPA chloride afforded the (*S*)- and (*R*)-bis-MTPA-esters, respectively. The observed chemical shift differences Δδ (δ_S_ − δ_R_) (Figure 3A) indicated 6*S*, *9S* configurations. The absolute configuration of C-5 stereocenter in **1** was proven as *R* on the basis of a characteristic Cotton’s effect at λ_217_ + 11.35 in the CD spectrum (Experimental Section and Figure S8) and a coupling constant value ^3^*J*_H5-H6_ = 4.4 Hz [14,19]. Compound **1** was recently reported as aspilactonol F, that was a component of unseparated mixture of epimers at C-9. Our study is the first determination of the absolute configurations of all stereocenters of aspilactonol F.

The molecular formula of compound **2** was determined as C_9_H_14_O_4_ (the same as **1**) on the basis of HRESIMS data and confirmed by ^13^C NMR. The NMR data of **2** were very similar to those of **1** (Table 1, Figures S9–S16). Thus, the planar structure of **2** was suggested to be the same as that of aspilactonol F (**1**).

Esterification of the C-6 and C-9 hydroxy moieties of **2** with (*R*)- and (*S*)-MTPA chloride afforded the (*S*)- and (*R*)-bis-MTPA-esters, respectively. The observed chemical shift differences Δδ (δ_S_ − δ_R_) (Figure 3B) indicated 6*R, 9S* configurations. The absolute configuration of the C-5 stereocenter in **2** was suggested as *S* on the basis of a strong negative Cotton’s effect at λ_216_ –11.51 in the CD spectrum (Experimental Section and Figure S17) [19]. Compound **2** was named aspilactonol G.

The molecular formula of compound **4** was established as C_22_H_28_O_9_ on the basis of HRESIMS, containing a peak at *m/z* 459.1628 [M + Na]^+^, and was supported by the ^13^C NMR spectrum.

An analysis of NMR data of **4** (Table 2, Figures S20–S24) revealed the presence of six methyl groups (δ_C_ 25.1, 24.0, 18.5, 17.3, 10.8, 9.5; δ_H_ 2.24, 1.89, 1.43, 1.41, 1.39, 1.31), one *sp*^3^-methylene group (δ_C_ 45.6; δ_H_ 2.86, 2.76), two *sp*^3^-methines (δ_C_ 39.5, 39.3; δ_H_ 2.32, 2.00), two oxygen-bearing ones (δ_C_ 75.15, 63.5; δ_H_ 4.63, 4.36), one quaternary *sp*^3^-carbon (δ_C_ 55.5), three oxygen-bearing quaternary *sp*^3^-carbons (δ_C_ 83.0, 76.5, 75.07), two quaternary *sp*^2^-carbons (δ_C_ 107.3, 102.2), three oxygen-bearing quaternary *sp*^2^-carbons (δ_C_ 164.4, 162.5, 157.9), and two ketone groups (δ_C_ 211.4, 209.1).

**Figure 4:**
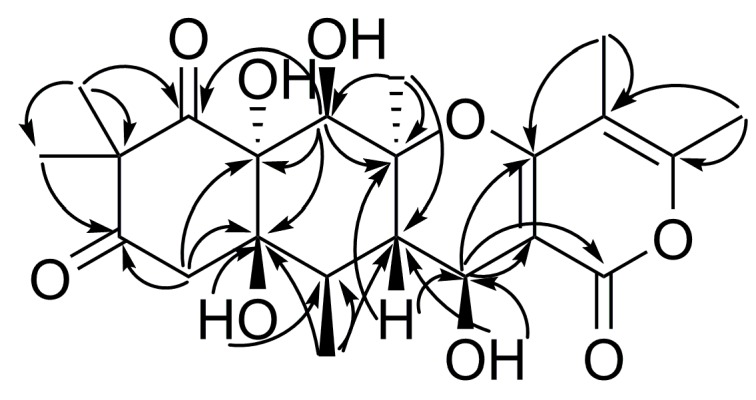
The key HMBC correlations of **4.**

The HMBC correlations of **4** (Figure 4 and Figure S25, Table 2) suggested the presence of a linear tetracyclic backbone like in the recently reported merosesquiterpenoids aspetetranones A-D [5]. The general features of the ^13^C NMR spectrum of **4** (Table 2, Figures S21–S22) were similar to those of aspertetranone D (**5**) [5], with the exception of the C-6, C-11, C-11a, C-12, C-15, and C-18 carbon signals. The main patterns of the experimental CD spectrum of **4** in methanol (Experimental section, Figure S27) matched well with those of aspertetranone D (**5**) [5]. The value of the vicinal coupling constant between H-11a and H-12 (9.4 Hz) in **4** instead of ^3^*J*_H11a-H12_ = 3.9 Hz in aspertetranone D (**5**) indicated a *β* orientation of the OH group at C-12 in **4**. Thus, the absolute configurations of chiral centers in **4** were suggested as 5a*S*, 6*R*, 6a*R*, 10a*R*, 11*R*, 11a*S*, 12*S*. Compound **4** was named 12-*epi*-aspertetranone D.

The molecular formula of compound **7** was established as C_15_H_22_O_5_ on the basis of an HRESIMS peak at *m*/*z* 305.1361 [M + Na]^+^, which was supported by the ^13^C NMR spectrum and corresponded to four double-bond equivalents.

A close inspection of the ^1^H and ^13^C NMR data of **7** (Table 3, Figures S30–S32) revealed the presence of two methyl groups (δ_C_ 26.8, 20.8; δ_H_ 1.23, 1.15), three *sp^3^*-methylene groups (δ_C_ 42.0, 32.6, 17.6; δ_H_ 2.13, 1.63, 1.50 (2H), 1.38, 1.24), two oxygen-bearing *sp*^3^-methylene groups (δ_C_ 75.0, 68.4; δ_H_ 4.44, 4.41, 4.24, 3.42), two *sp*^3^-methine groups (δ_C_ 63.5, 47.1; δ_H_ 4.62, 2.00), including one oxygen-bearing, one *sp*^2^-methine group (δ_C_ 139.1; δ_H_ 6.96), three quaternary *sp*^3^-carbons (δ_C_ 77.5, 39.0, 38.3), including one oxygen-bearing, and two quaternary *sp*^2^-carbons (δ_C_ 169.6, 130.1).

The ^13^C NMR data of **7** were similar to those of the drimane moiety of insulicolide A (**8**) [15], also reported as 9α-14-dihydroxy-6β-*p*-nitrobenzoylcinnamolide [8], with the exception of the C-3, C-6, C-7, C-8, and C-14 carbon signals. The COSY spectrum data (Figure S33) and HMBC correlations (Figure S35, Table 3) from H-6 (δ_H_ 4.62) to C-7 (δ_C_ 139.1), C-8 (δ_C_ 130.1), and C-10 (δ_C_ 39.0), from H-7 (δ_H_ 6.96) to C-5 (δ_C_ 47.1), C-9, and C-12 (δ_C_ 169.6), from H_3_-13 (δ_H_ 1.15) to C-3 (δ_C_ 42.0), C-4 (δ_C_ 38.3), C-5 (δ_C_ 47.1), and C-14 (δ_C_ 68.4), and from H_3_-15 (δ_H_ 1.23) to C-1 (δ_C_ 32.6), C-5, C-9, and C-10 proved the drimane framework of **7** the same as in insulicolide A (**8**).

The ROESY correlations (Figure S36) of H_3_-13 with H-5 (δ_H_ 2.00) and H-6, long-range COSY correlation H_3_-15/H-5, together with the vicinal coupling constant ^3^*J*_H5-H6_ = 4.4 Hz established the relative configurations of the C-4, C-5, C-6, and C-10 chiral centers. The absolute configurations of the stereocenters in **7** were suggested as depicted in Figure 1 from CD spectra similarity (Figures S37 and S38) and biogenetic relationship with insulicolide A (8), whose absolute configurations were determined previously by X-ray analysis [15]. Compound **7** was named 6β,9α,14-trihydroxycinnamolide.

The molecular formula of compound **9** was established as C_15_H_22_O_5_ on the basis of an HRESIMS peak at *m*/*z* 305.1361 [M + Na]^+^, which was supported by the ^13^C NMR spectrum.

A close inspection of the ^1^H and ^13^C NMR data of **9** (Table 3, Figures S39–S41) revealed the presence of two methyl groups (δ_C_ 27.9, 21.6; δ_H_ 1.40, 0.97), three *sp*^3^-methylene groups (δ_C_ 37.8 (2C), 18.0; δ_H_ 1.71, 1.59, 1.54, 1.45, 1.32, 1.10), two oxygen-bearing *sp^3^*-methylene groups (δ_C_ 68.1, 65.6; δ_H_ 4.94, 4.79, 3.94, 3.26), three *sp*^3^-methine groups (δ_C_ 70.0, 64.1, 48.6; δ_H_ 4.00, 3.99, 1.57), including two oxygen-bearing, two quaternary *sp*^3^-carbons (δ_C_ 38.3, 36.3), and three quaternary *sp*^2^-carbons (δ_C_ 173.4, 173.1, 122.1).

The HMBC correlations (Table 3, Figure S42) from H-6 (δ_H_ 3.99) to C-5 (δ_C_ 48.6), C-7 (δ_C_ 64.1), C-8 (δ_C_ 122.1), C-9 (δ_C_ 173.1), and C-10 (δ_C_ 36.3), from H-7 (δ_H_ 4.00) to C-12 (δ_C_ 173.4), from H_2_-11 (δ_H_ 4.94, 4.79) to C-8, C-9, and C-12, from H_3_-13 (δ_H_ 0.97) to C-3 (δ_C_ 37.8), C-4 (δ_C_ 38.3), C-5, and C-14 (δ_C_ 65.6), from H_3_-15 (δ_H_ 1.40) to C-1 (δ_C_ 37.8), C-5, C-9, and C-10 indicated the drimane moiety in **9** being the same as in 7α,14-dihydroxy-6β-p-nitrobenzoylconfertifolin [8].

**Figure 5:**
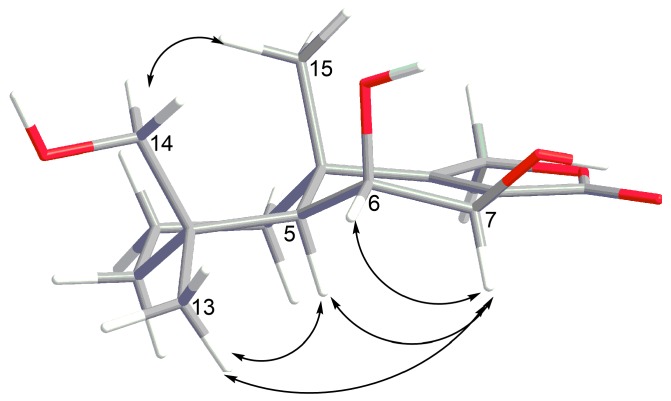
Key ROESY correlations of **9**.

The ROESY correlations (Figure 5 and Figure S43) of H_3_-13 with H-5 (δ_H_ 1.57), H-6 (δ_H_ 3.99), and H-7 (δ_H_ 4.00), of H_3_-15 with H_2_-14 (δ_H_ 3.94, 3.26), together with the coupling constant ^3^*J*_H6-H7_ = 2.1 Hz indicated the related configurations of the chiral centers in **9** as depicted (Figure 1). Compound **9** was named 6β,7β,14-trihydroxyconfertifolin.

Besides the new compounds **1,2,4,7**, and **9**, the known dihydroaspirone (**3**) [14], aspertetranones D (**5**) [5,6] and A (**6**) [5], insulicolide A (**8**) [15], and 7α,14-dihydroxy-6β-p-nitrobenzoylconfertifolin (**10**) [8] were isolated from this fungal strain.

All isolated compounds were tested for cytotoxicity toward murine neuroblastoma Neuro-2a cells (Table 4). Compound **7** demonstrated cytotoxic activity toward Neuro-2a cell, with the IC_50_ of 24.1 μM, while its analogue **9** was non-cytotoxic up to 100 μM. The highest activity was demonstrated for 9α,14-dihydroxy-6β-p-nitrobenzoylcinnamolide (**8**), with IC_50_ of 4.9 μM, while its analogue **10** did not affect the viability of Neuro-2a cells. Compounds **1**–**6** were non-cytotoxic against Neuro-2a cells at concentrations up to 100 μM.

Then, we investigated the effect of the compounds **1**–**10** on the viability and colony formation ability of human drug-resistant prostate cancer 22Rv1 cells (Table 4). MTT assay revealed the compounds **7** and **8** to be cytotoxic in 22Rv1 cells, with IC_50_ values of 31.5 µM and 3.0 µM, respectively. Compounds **1**–**6, 9**, and **10** were non-cytotoxic against these cells at concentrations up to 100 µM. In this model, docetaxel (positive control) showed cytotoxicity, with IC_50_ of 0.02 µM. At the same time, compounds **4** and **9** were able to inhibit the colony formation of 22Rv1 prostate cancer cells (in vitro prototype of in vivo anti-metastatic activity) for 41% and 36%, respectively, at 100 µM. It is known that 22Rv1 cells are resistant to hormone therapy because they express the androgen receptor splice variant AR-V7 [20]. The compounds which demonstrated cytotoxic activity toward AR-V7-positive 22Rv1 cells therefore may be promising for the therapy of human drug-resistant prostate cancer.

Finally, the new compounds **7** and **9** were tested for cytotoxicity toward human breast cancer cells MCF-7 and did not show any effect up to 100 µM (Table 4). Additionally, the known compounds **8** and **10** were examined in this experiment as reference substances. Compound **8** showed a weak cytotoxic effect, with IC_50_ of 59.6 μM, whereas, previously, a higher cytotoxicity of **8** toward MCF-7 cells was reported (IC_50_ = 6.08 μM) [11]. This could be explained by different treatment times used by us (24 h) in comparison with those used by Fang and colleagues (72 h) [11]. Moreover, different amounts of cells per well were used. Note, compound **10** was non-cytotoxic up to 100 µM.

The analysis of structure–activity relationships of compounds **7–10,** together with literature data, showed that these compounds have three relevant structural sites. First, a double bond at C7=C8 as part of an α,β-unsaturated lactone. Previously, it was shown that the cytotoxicity of such moiety can be explained by a nucleophilic Michael addition reaction with biological nucleophiles [8,21]. In the case of the non-cytotoxic compounds **9** and **10**, the double bond of the α,β-unsuturated lactone may be inaccessible for a nucleophile attack because of steric obstacles. Second, a hydroxyl group at C-9 in the drimane core is also essential for cytotoxicity. In fact, a recent report of a series of similar compounds revealed the most pronounced cytotoxicity for compounds possessing a 9-OH group [9]. Finally, our results strongly suggest that the presence of a *p*-nitrobenzoyl moiety significantly enhances the cytotoxic activity. Previously, Tan et al. [9] demonstrated that the nitrobezoylation of 6-OH increased the cytotoxicity of related compounds towards human renal cell carcinoma cells compared with that of 14-OH-derivatives. At the same time, it should be noted that another study of 6- and 14-nitrobenzoate derivatives cytotoxicity toward other cancer cell lines did not support this observation [11].

## 3. Materials and Methods

### 3.1. General Experimental Procedures

Optical rotations were measured on a Perkin-Elmer 343 polarimeter (Perkin Elmer, Waltham, MA, USA). UV spectra were recorded on a Specord UV−vis spectrometer (Carl Zeiss, Jena, Germany) in methanol. NMR spectra were recorded in CDCl_3_, acetone-d_6_ and DMSO-*d*_6_ with Bruker DPX-500 (Bruker BioSpin GmbH, Rheinstetten, Germany) and Bruker DRX-700 (Bruker BioSpin GmbH, Rheinstetten, Germany) spectrometers, using TMS as an internal standard. HRESIMS spectra were measured on a Maxis impact mass spectrometer (Bruker Daltonics GmbH, Rheinstetten, Germany).

Low-pressure liquid column chromatography was performed using silica gel (50/100 μm, Imid, Russia). Plates (4.5 cm × 6.0 cm) precoated with silica gel (5–17 μm, Imid) were used for thin-layer chromatography. Preparative HPLC was carried out with a Shimadzu LC-20 chromatograph (Shimadzu USA Manufacturing, Canby, OR, USA) using YMC ODS-AM (YMC Co., Ishikawa, Japan) (5 µm, 10 mm × 250 mm) and YMC SIL (YMC Co., Ishikawa, Japan) (5 µm, 10 mm × 250 mm) columns with a Shimadzu RID-20A refractometer (Shimadzu Corporation, Kyoto, Japan).

### 3.2. Fungal Strain

The strain of *A. flocculosus* was isolated from a sediment sample (Nha Trang Bay, South China Sea, Vietnam) and identified as described earlier [12]. The strain is stored at the collection of microorganisms of Nha Trang Institute of Technology and Research Application VAST (Nha Trang, Vietnam) under the code 01NT.1.12.3.

### 3.3. Cultivation of the Fungus

The fungus was cultured at 28 °C for three weeks in 50 × 500 mL Erlenmeyer flasks, each containing rice (20.0 g), yeast extract (20.0 mg), KH_2_PO_4_ (10 mg), and natural sea water from Nha Trang Bay (40 mL).

### 3.4. Extraction and Isolation

The fungal mycelia of *A. flocculosus* with the medium were extracted for 24 h with 15 L of EtOAc. Evaporation of the solvent, under reduced pressure, gave a dark brown oil (5.0 g), to which 250 mL H_2_O–EtOH (4:1) was added, and the mixture was thoroughly stirred to yield a suspension. It was extracted, successively, with hexane (150 mL × 2), EtOAc (150 mL × 2), and n-BuOH (150 mL × 2). After evaporation of the EtOAc layer, the residual materials (3.36 g) were passed over a silica gel column (35.0 cm × 2.5 cm), which was eluted with a hexane–EtOAc gradient (1:0–0:1). The n-hexane–EtOAc (80:20, 1.3 g) fraction was purified by a Sephadex LH-20 column (80 cm × 2 cm, 50 g) with CHCl_3_ to yield compound **8** (245 mg). The n-hexane–EtOAc (75:25) fraction AF-1-64 (393 mg) was purified by HPLC on a YMC-SIL column eluting with CHCl_3_–MeOH–NH_4_OAc (97:3:1) to yield compounds **3** (220 mg) and **4** (11 mg). The n-hexane–EtOAc (75:25) fraction AF-1-67 (483 mg) was purified by HPLC on a YMC-SIL column eluting with CHCl_3_–MeOH–NH_4_OAc (97:3:1) to yield compounds **5** (5.9 mg), **7** (9.0 mg), and **10** (3.1 mg). The n-hexane–EtOAc (75:25) fraction AF-1-88 (68.3 mg) was purified by HPLC on a YMC-SIL column eluting with CHCl_3_–MeOH–NH_4_OAc (97:3:1) to yield compounds **1** (2.9 mg) and **2** (3.8 mg). The n-hexane–EtOAc (70:30) fraction AF-1-93 (784 mg) was purified by HPLC first on a YMC-SIL column eluting with CHCl_3_–MeOH–NH_4_OAc (97:3:1) and then on a YMC ODS-AM column, eluting with MeOH–H_2_O (55:45) to yield compound **9** (5.5 mg). The n-hexane–EtOAc (60:40, 282 mg) fraction was purified by Sephadex LH-20 column (80 cm × 2 cm, 50 g) with CHCl_3_-EtOH (3:1) to yield compound **6** (68 mg).

Aspilactonol F (**1**): white powder; ${\left[\alpha \right]}_{D}^{20}$ +98 (*c* 0.20, MeOH); UV (MeOH) *λ*_max_ (log *ε*) 214 (4.03) nm; ECD (0.9 mM, MeOH) *λ*_max_ (Δ*ε*) 217 (+11.35) nm; ^1^H and ^13^C NMR data see Table 1, Figures S1–S7; HR ESIMS *m*/*z* 209.0785 [M + Na]^+^ (calcd. for C_9_H_14_O_4_Na, 209.0784, Δ −0.1 ppm).

Aspilactonol G (**2**): white powder; ${\left[\alpha \right]}_{D}^{20}$ –49 (*c* 0.49, MeOH); UV (MeOH) *λ*_max_ (log *ε*) 214 (4.05) nm; ECD (1.1 mM, MeOH) *λ*_max_ (Δ*ε*) 216 (–11.51) nm; ^1^H and ^13^C NMR data see Table 1, Figures S9–S16; HRESIMS *m*/*z* 209.0782 [M + Na]^+^ (calcd. for C_9_H_14_O_4_Na, 209.0784, Δ +1.1 ppm).

12-*Epi*-aspertetranone D (**4**): white powder; ${\left[\alpha \right]}_{D}^{20}$ +78 (*c* 0.07, MeOH); UV (MeOH) *λ*_max_ (log *ε*) 290 (3.93), 208 (4.53) nm; ECD (0.5 mM, MeOH) *λ*_max_ (Δ*ε*) 209 (+25.54), 284 (+1.86) nm; ^1^H and ^13^C NMR data see Table 2, Figures S20–S26; HRESIMS *m*/*z* 459.1628 [M + Na]^+^ (calcd. for C_22_H_28_O_9_Na, 459.1626, Δ −0.2 ppm).

6β,9α,14-trihydroxycinnamolide (**7**): white crystals; ${\left[\alpha \right]}_{D}^{20}$ –7.3 (*c* 0.15, MeOH); UV (MeOH) *λ*_max_ (log *ε*) 206 (3.61) nm; ECD (2.8 mM, MeOH) *λ*_max_ (Δ*ε*) 224 (–2.33) nm; ^1^H and ^13^C NMR data see Table 3, Figures S30–S36; HRESIMS *m*/*z* 305.1361 [M + Na]^+^ (calcd. for C_15_H_22_O_5_Na, 305.1359, Δ −0.5 ppm).

6β,7β,14-trihydroxyconfertifolin (**9**)*:* white crystals; ${\left[\alpha \right]}_{D}^{20}$ +93.5 (*c* 0.36, MeOH); UV (MeOH) *λ*_max_ (log *ε*) 214 (4.00) nm; ECD (1.1 mM, MeOH) *λ*_max_ (Δ*ε*) 217 (+3.68), 243 (+1.51) nm; ^1^H and ^13^C NMR data see Table 3, Figures S39–S47; HRESIMS *m*/*z* 305.1361 [M + Na]^+^ (calcd. for C_15_H_22_O_5_Na, 305.1359, Δ −0.5 ppm).

### 3.5. Preparation of (S)-MTPA and (R)-MTPA Esters of Aspilactonol F (1)

The compounds 4-dimethylaminopyridine (a few crystals) and (*R*)-MTPA-Cl (4 μL) were added to a solution of **1** (1.0 mg) in pyridine at room temperature and stirred for 5 h. After evaporation of the solvent, the residue was purified by HPLC on a YMC SIL column (EtOAc–hexane, 20:80) to afford the (*S*)-MTPA ester (0.5 mg). The (*R*)-MTPA ester (0.5 mg) was prepared in a similar manner using (*S*)-MTPA-Cl.

(*S*)-MTPA ester of **1**: ^1^H NMR (CDCl_3_, 500.13 MHz) δ: 6.88 (1H, brs, H-4), 5.28-5.34 (2H, m, H-6, H-9), 4.84 (1H, dd, *J =* 3.9; 1.7 Hz, H-5), 3.48 (3H, s, OMe), 3.43 (3H, s, OMe), 2.56-2.60 (2H, m, H_2_-8), 1.26 (3H, d, *J =* 6.5 Hz, Me-7), 1.24 (3H, d, *J =* 6.3 Hz, Me-10), 7.39–7.48 (10H, m, 2Ph). HRESIMS *m/z* 641.1576 [M + Na]^+^ (calcd for C_29_H_28_F_6_Na, 641.1581, Δ = 0.8 ppm).

(*R*)-MTPA ester of **1**: ^1^H NMR (CDCl_3_, 500.13 MHz) δ: 6.52 (1H, brs, H-4), 5.25 (1H, m, H-9), 5.20 (1H, dd, *J =* 6.6, 4.3 Hz, H-6), 4.56 (1H, dd, *J =* 4.3, 1.6 Hz, Hz, H-5), 3.56 (3H, s, OMe), 3.50 (3H, s, OMe), 2.48-2.51 (2H, m, H_2_-8), 1.35 (3H, d, *J =* 6.2 Hz, Me-10), 1.29 (3H, d, *J =* 6.6 Hz, Me-7), 7.38–7.52 (10H, m, 2Ph). HRESIMS *m/z* 641.1577 [M + Na]^+^ (calcd for C_29_H_28_F_6_Na, 641.1581, Δ = 0.6 ppm).

### 3.6. Preparation of (S)-MTPA and (R)-MTPA Esters of Aspilactonol G (2)

(*R*)-MTPA-Cl (9 μL) was added to a solution of **2** (1.9 mg) in pyridine at room temperature and stirred for 2 h. After evaporation of the solvent, the residue was purified by HPLC on a YMC SIL column (acetone–hexane, 25:75) to afford the (*S*)-MTPA ester (1.4 mg). The (*R*)-MTPA ester (1.5 mg) was prepared in a similar manner using (*S*)-MTPA-Cl.

(*S*)-MTPA ester of **2**: ^1^H NMR (CDCl_3_, 700 MHz) δ: 6.86 (1H, brs, H-4), 5.32 (1H, m, H-9), 5.23 (1H, m, H-6), 4.81 (1H, brd, *J =* 5.0 Hz, H-5), 3.52 (3H, s, OMe), 3.47 (3H, s, OMe), 2.65 (1H, dd, *J* = 15.8; 6.9, H-8), 2.48 (1H, ddt, *J* = 15.9; 5.0; 1.5, H-8), 1.39 (3H, d, *J =* 6.5 Hz, Me-7), 1.29 (3H, d, *J =* 6.2 Hz, Me-10), 7.38–7.50 (10H, m, 2Ph). HRESIMS *m/z* 641.1576 [M + Na]^+^ (calcd for C_29_H_28_F_6_Na, 641.1581, Δ = 0.8 ppm).

(*R*)-MTPA ester of **2**: ^1^H NMR (CDCl_3_, 700 MHz) δ: 6.68 (1H, brs, H-4), 5.30 (1H, m, H-9), 5.26 (1H, m, H-6), 4.82 (1H, m, Hz, H-5), 3.53 (3H, s, OMe), 3.48 (3H, s, OMe), 2.61 (1H, dd, *J* = 15.9; 7.2, H-8), 2.46 (1H, dd, *J* = 15.9; 4.7, H-8), 1.33 (3H, d, *J =* 6.3 Hz, Me-10), 1.25 (3H, d, *J =* 6.7 Hz, Me-7), 7.37–7.52 (10H, m, 2Ph). HRESIMS *m/z* 641.1576 [M + Na]^+^ (calcd for C_29_H_28_F_6_Na, 641.1581, Δ = 0.8 ppm).

### 3.7. Cell Culture

All cell lines used in this investigation were purchased from ATCC.

The neuroblastoma cell line Neuro-2a and the human breast cancer cell line MCF-7 were cultured in DMEM medium containing 10% fetal bovine serum (Biolot, St. Petersburg, Russia) and 1% penicillin/streptomycin (Invitrogen, Carlsbad, CA, USA).

The human prostate cancer cell line 22Rv1 was cultured according to the manufacturer’s instructions in 10% FBS/RPMI medium (Invitrogen). Cells were continuously kept in culture for a maximum of 3 months, were routinely inspected microscopically for stable phenotype, and regularly checked for contamination with mycoplasma. Cell line authentication was performed by DSMZ (Braunschweig, Germany) using highly polymorphic short tandem repeat loci [22].

All cells were incubated at 37 °C in a humidified atmosphere containing 5% (*v/v*) CO_2_.

### 3.8. Cytotoxicity Assay

The in vitro cytotoxicity of individual substances was evaluated using the MTT assay, which was performed as previously described [23]. Docetaxel was used as a control.

### 3.9. Colony Formation Assay

The colony formation assay was performed as described before with slight modifications [22]. 22Rv1 cells were treated with the testing compounds for 48 h and then were trypsinized. The number of alive cells was counted with the trypan blue exclusion assay as described before [24]. In total, 100 viable cells were plated into each well of six-well plates in complete fresh medium (3 mL/well) and were incubated for 14 days. Then, the medium was aspirated, and the surviving colonies were fixed with 100% MeOH, followed by washing with PBS, and air-drying at RT. Next, the cells were incubated with a Giemsa staining solution was for 25 min at RT, the staining solution was aspirated, and the wells were rinsed with dH_2_O and air-dried. The number of cell colonies was counted by naked eye.

## 4. Conclusions

A new aspyrone-related polyketide, aspilactonol G (**2**), a new meroterpenoid, 12-*epi*-aspertetranone D (**4**), two new drimane derivatives (**7**,**9**), together with six known metabolites were isolated from the Vietnamese marine sediment-derived fungus *A. flocculosus*. The structures of compounds **1**–**10** were established using spectroscopic methods. The absolute configurations of chiral centers were determined using either a modified Mosher’s method (for compounds **1** and **2**) or a combination of ROESY data, coupling constants analysis and biogenetic considerations for compounds **4**, **7** and **9**. Drimane sesquiterpenoid derivatives **7** and **8** showed cytotoxicity toward human prostate cancer 22Rv1, human breast cancer MCF-7, and murine neuroblastoma Neuro-2a cells. The analysis of structure–activity relationships of compounds **7**–**10** together with literature data showed that these compounds have three sites in their structures related to cytotoxicity, i.e., a double bond at C7=C8, a hydroxyl group at C-9, and a *p*-nitrobenzoyl moiety.

## Figures and Tables

**Table 1:** ^1^H and ^13^C NMR data (*δ* in ppm, CDCl_3_) for aspilactonols G (**1**) and F (**2**).

Position	1		2	
	*δ*_C_, mult	*δ*_H_ (*J* in Hz)	*δ*_C_, mult	*δ*_H_ (*J* in Hz)
2	174.2, C		174.1, C	
3	132.8, C		132.9, C	
4	147.4, CH	7.27, d (1.4)	147.3, CH	7.25, d (1.2)
5	84.9, CH	4.85, dd (4.4, 1.4)	84.8, CH	4.86, dd (4.2, 1.4)
6	67.8, CH	4.05, qd (6.4, 4.4)	67.6, CH	4.08, qd (6.6, 4.2)
7	18.8, CH_3_	1.31, d (6.4)	18.8, CH_3_	1.31, d (6.6)
8	34.9, CH_2_	2.52, ddt (15.0, 3.8, 1.4) 2.45, ddt (15.0, 7.8, 1.4)	35.2, CH_2_	2.55, ddt (14.6, 3.6, 1.4) 2.40, dd (14.6, 8.5)
9	66.2, CH	4.08, m	65.8, CH	4.04, m
10	23.3, CH_3_	1.25, d (6.3)	23.2, CH_3_	1.25, d (6.2)

^1^H NMR and ^13^C NMR spectroscopic data were measured at 500 MHz and 125 MHz, respectively.

**Table 2:** ^1^H and ^13^C NMR data (*δ* in ppm, CDCl_3_) for 12-*epi*-aspertetranone D (**4**).

Position	*δ*_C_, Mult	*δ*_H_ (*J* in Hz)	HMBC
1	164.4, C		
3	157.9, C		
4	107.3, C		
4a	162.5, C		
5a	83.0, C		
6	75.15, CH	4.36, s	5a, 6a, 7, 10a, 11a, 15
6a	76.5, C		
7	211.4, C		
8	55.5, C		
9	209.1, C		
10	45.6, CH_2_	2.86, d (17.7) 2.76, dd (17.7, 2.7)	6a, 9, 10a 9, 10a
10a	75.07, C		
11	39.5, CH	2.00, dd (12.0, 6.8)	5a, 10a, 11a, 18
11a	39.3, CH	2.32, dd (12.0, 9.4)	5a, 6, 10a, 11, 12, 18
12	63.5, CH	4.63, d (9.4)	1, 4a, 11, 11a, 12a
12a	102.2, C		
13	17.3, CH_3_	2.24, s	3, 4, 4a
14	9.5, CH_3_	1.89, s	3, 4, 4a
15	18.5, CH_3_	1.43, s	5a, 6, 11a
16	25.1, CH_3_	1.39, s	7, 8, 9, 17
17	24.0, CH_3_	1.41, s	7, 8, 9, 16
18	10.8, CH_3_	1.31, d (6.8)	10a, 11, 11a
6-OH		3.57, brs	
6a-OH		3.12, brs	
10a-OH		4.01, d (2.7)	10, 10a
12-OH		4.43, brs	11a, 12

^1^H NMR and ^13^C NMR spectroscopic data were measured at 500 MHz and 125 MHz, respectively.

**Table 3:** ^1^H and ^13^C NMR data (*δ* in ppm) for 6β,9α,14-trihydroxycinnamolide (**7**) and 6β,7β,14-trihydroxyconfertifolin (**9**).

Position	7 ^a^			9 ^b^		
	*δ*_C_, mult	*δ*_H_ (*J* in Hz)	HMBC	*δ*_C_, mult	*δ*_H_ (*J* in Hz)	HMBC
1	32.6, CH_2_	1.24, m 2.13, td (12.7, 5.7)	2, 3, 5, 9, 10, 15	37.8, CH_2_	1.59, m 1.54, m	2, 3, 5, 15
2	17.6, CH_2_	1.50, m	1, 3, 4	18.0, CH_2_	1.71, m 1.45, m	1, 3
3	42.0, CH_2_	1.38, td (12.9, 5.3) 1.63, m	2, 4, 13, 14 1, 2, 4, 5, 14	37.8, CH_2_	1.32, td (13.0, 3.8) 1.10, td (13.6, 4.3)	1, 2, 13, 14
4	38.3, C			38.3, C		
5	47.1, CH	2.00, d (4.0)	4, 6, 9, 13, 14, 15	48.6, CH	1.57, brs	1, 6, 9, 10, 14, 15
6	63.5, CH	4.62, t (4.2)	7, 8, 10	70.0, CH	3.99, brs	5, 7, 8, 9, 10
7	139.1, CH	6.96, d (4.0)	5, 9, 12	64.1, CH	4.00, d (2.1)	5, 6, 12
8	130.1, C			122.1, C		
9	77.5, C			173.1, C		
10	39.0, C			36.3, C		
11	75.0, CH_2_	4.24, d (9.8) 4.44, d (9.8)	8, 9, 12	68.1, CH_2_	4.94, dd (17.6, 1.7) 4.79, brd (17.6)	7, 8, 9
12	169.6, C			173.4, C		
13	26.8, CH_3_	1.15, s	3, 4, 5, 14	27.9, CH_3_	0.97, s	3, 4, 5, 14
14	68.4, CH_2_	3.42, d (11.4) 4.41, d (11.4)	3, 4, 5, 13	65.6, CH_2_	3.94, dd (11.3, 3.8) 3.26, dd (11.3, 6.0)	3, 4, 5, 13
15	20.8, CH_3_	1.23, s	1, 5, 9, 10	21.6, CH_3_	1.40, s	1, 5, 9, 10

^1^H NMR and ^13^C NMR spectroscopic data were measured ^a^ in CDCl_3_ at 500 MHz and 125 MHz, respectively, and ^b^ in DMSO-d_6_ at 700 MHz and 176 MHz, respectively.

**Table 4:** Cytotoxic effects of the isolated compounds **1**–**10**.

Compounds	Cytotoxicity IC_50_, µM			Colony Formation, %
	Neuro-2a	22Rv1	MCF-7	22Rv1
1	>100	>100	nt	-
2	>100	>100	nt	-
3	>100	>100	nt	-
4	>100	>100	nt	41
5	>100	>100	nt	-
6	>100	>100	nt	-
7	24.1	31.5	>100	-
8	4.9	3.0	59.6	-
9	>100	>100	>100	36
10	>100	>100	>100	-
Docetaxel	nt	0.02	nt	nt

“nt”: compound was not tested; “-“: compound did not demonstrate any effect at the concentration of 100 µM.

## Supplementary Materials

The following are available online at https://www.mdpi.com/1660-3397/17/10/579/s1, Figures S1–S57: 1D and 2D NMR spectra and ECD spectra of compounds **1**–**10**.
