# Supplementary material for: Biologically Active Metabolites from the Marine Sediment-Derived Fungus Aspergillus flocculosus
Source: Mar Drugs. 2019 Oct 11;17(10):579. doi: 10.3390/md17100579 (PMC6835654; doi:10.3390/md17100579)

Supplementary data

Biologically Active Metabolites from the Marine Sediment-Derived Fungus *Aspergillus flocculosus*

Anton N. Yurchenko [1,*], Phan Thi Hoai Trinh [2], Elena V. Ivanets [1], Olga F. Smetanina [1], Anton B. Rasin [1], Roman S. Popov [1], Sergey A. Dyshlovoy [1,3,4,5], Gunhild von Amsberg [4,5], Ekaterina S. Menchinskaya [1], Tran Thi Thanh Van [2] and Shamil Sh. Afiyatullov [1]

[1] G.B. Elyakov Pacific Institute of Bioorganic Chemistry, Far Eastern Branch of the Russian Academy of Sciences, Prospect 100-letiya Vladivostoka, 159, Vladivostok 690022, Russia; ev.ivanets@yandex.ru (E.V.I.), smetof@rambler.ru (O.F.S.); abrus__54@mail.ru (A.B.R.); prs_90@mail.ru (R.S.P.); dyshlovoy@gmail.com (S.A.D.); ekaterinamenchinskaya@gmail.com (E.S.M.); afiyat@piboc.dvo.ru (S.S.A.)

[2] Department of Marine Biotechnology, Nhatrang Institute of Technology Research and Application, Vietnam Academy of Science and Technology, Nha Trang, Vietnam; phanhoaitrinh84@gmail.com (P.T.H.T.); tranthanhvan@nitra.vast.vn (T.T.T.V.)

[3] School of Natural Science, Far Eastern Federal University, Sukhanova St., 8, Vladivostok 690000, Russia

[4] Laboratory of Experimental Oncology, Department of Oncology, Hematology and Bone Marrow Transplantation with Section Pneumology, Hubertus Wald-Tumorzentrum, University Medical Center Hamburg-Eppendorf, 20246 Hamburg, Germany; g.von-amsberg@uke.de (G.A.)

[5] Martini-Klinik Prostate Cancer Center, University Hospital Hamburg-Eppendorf, Hamburg, Germany

* Correspondence: yurchant@ya.ru; Tel.: +7-423-231-1168

Content

Figure S1. ^{1}H NMR (500 MHz, $CDCl_3$) spectrum of aspilactonol F (**1**) 4
Figure S2. ^{13}C NMR (125 MHz, $CDCl_3$) spectrum of aspilactonol F (**1**) 5
Figure S3. DEPT-135 (125 MHz, $CDCl_3$) spectrum of aspilactonol F (**1**) 6
Figure S4. ^{1}H-^{1}H COSY (500 MHz, $CDCl_3$) spectrum of aspilactonol F (**1**) 7
Figure S5. HSQC (500 MHz, $CDCl_3$) spectrum of aspilactonol F (**1**) 8
Figure S6. HMBC (500 MHz, $CDCl_3$) spectrum of aspilactonol F (**1**) 9
Figure S7. ROESY (500 MHz, $CDCl_3$) spectrum of aspilactonol F (**1**).......................................10
Figure S8. ECD spectrum of aspilactonol F (**1**) in methanol 11
Figure S9. ^{1}H NMR (500 MHz, $CDCl_3$) spectrum of aspilactonol G (**2**)....................................12
Figure S10. ^{13}C NMR (125 MHz, $CDCl_3$) spectrum of aspilactonol G (**2**)................................13
Figure S11. DEPT-135 (125 MHz, $CDCl_3$) spectrum of aspilactonol G (**2**)...............................14
Figure S12. ^{1}H NMR (700 MHz, acetone-d_6) spectrum of aspilactonol G (**2**)............................15
Figure S13. ^{13}C NMR (125 MHz, acetone-d_6) spectrum of aspilactonol G (**2**)...........................16
Figure S14. ^{1}H-^{1}H COSY (700 MHz, acetone-d_6) spectrum of aspilactonol G (**2**)......................17
Figure S15. HSQC (700 MHz, acetone-d_6) spectrum of aspilactonol G (**2**) 18
Figure S16. HMBC (700 MHz, acetone-d_6) spectrum of aspilactonol G (**2**) 19
Figure S17. ECD spectrum of aspilactonol G (**2**) in methanol..20
Figure S18. ^{1}H NMR (500 MHz, $CDCl_3$) spectrum of dihydroaspirone (**3**) 21
Figure S19. ^{13}C NMR (176 MHz, $CDCl_3$) spectrum of dihydroaspirone (**3**) 22
Figure S20. ^{1}H NMR (500 MHz, $CDCl_3$) spectrum of 12-*epi*-aspertetranone D (**4**) 23
Figure S21. ^{13}C NMR (176 MHz, $CDCl_3$) spectrum of 12-*epi*-aspertetranone D (**4**) 25
Figure S22.DEPT-135 (500 MHz, $CDCl_3$) spectrum of 12-*epi*-aspertetranone D (**4**) 26
Figure S23. ^{1}H-^{1}H COSY (500 MHz, $CDCl_3$) spectrum of 12-*epi*-aspertetranone D (**4**) 27
Figure S24. HSQC (500 MHz, $CDCl_3$) spectrum of 12-*epi*-aspertetranone D (**4**) 28
Figure S25. HMBC (500 MHz, $CDCl_3$) spectrum of 12-*epi*-aspertetranone D (**4**)......................29
Figure S26. ROESY (500 MHz, $CDCl_3$) spectrum of 12-*epi*-aspertetranone D (**4**).....................30
Figure S27. ECD spectrum of 12-*epi*-aspertetranone D (**4**) in methanol 31
Figure S28. ^{1}H NMR (500 MHz, $CDCl_3$) spectrum of aspertetranone D (**5**)...............................32
Figure S29. ^{13}C NMR (176 MHz, $CDCl_3$) spectrum of aspertetranone D (**5**).............................33
Figure S30. ^{1}H NMR (500 MHz, acetone-d_6) spectrum of aspertetranone A (**6**).........................34
Figure S31. ^{13}C NMR (125 MHz, acetone-d_6) spectrum of aspertetranone A (**6**).......................35
Figure S32. DEPT-135 (125 MHz, acetone-d_6) spectrum of aspertetranone A (**6**)......................36
Figure S33. ^{1}H NMR (500 MHz, $CDCl_3$) spectrum of 6β,9α,14-trihydroxycinnamolide (**7**)......37
Figure S34. ^{13}C NMR (500 MHz, $CDCl_3$) spectrum of 6β,9α,14-trihydroxycinnamolide (**7**).....38
Figure S35. DEPT-135 (500 MHz, $CDCl_3$) spectrum of 6β,9α,14-trihydroxycinnamolide (**7**)...39

Figure S36. ^{1}H-^{1}H COSY (500 MHz, $CDCl_3$) spectrum of 6β,9α,14-trihydroxycinnamolide (**7**) 40
Figure S37. HSQC (500 MHz, $CDCl_3$) spectrum of 6β,9α,14-trihydroxycinnamolide (**7**)..........41
Figure S38. HMBC (500 MHz, $CDCl_3$) spectrum of 6β,9α,14-trihydroxycinnamolide (**7**) 42
Figure S39. ROESY (500 MHz, $CDCl_3$) spectrum of 6β,9α,14-trihydroxycinnamolide (**7**) 43
Figure S40. ECD spectrum of 6β,9α,14-trihydroxycinnamolide (**7**) in methanol.......................44
Figure S41. ^{1}H NMR (500 MHz, acetone-d_6) spectrum of insulicolide A (**8**) 45
Figure S42. ^{13}C NMR (125 MHz, acetone-d_6) spectrum of insulicolide A (**8**) 47
Figure S43. DEPT-135 (125 MHz, acetone-d_6) spectrum of insulicolide A (**8**) 48
Figure S44. ECD spectrum of insulicolide A (**8**) in methanol 49
Figure S45. ^{1}H NMR (700 MHz, DMSO-d_6) spectrum of 6β,7α,14-trihydroxyconfertifolin (**9**) 50
Figure S46. ^{13}C NMR (125 MHz, DMSO-d_6) spectrum of 6β,7α,14-trihydroxyconfertifolin (**9**) 52
Figure S47. HSQC (700 MHz, DMSO-d_6) spectrum of 6β,7α,14-trihydroxyconfertifolin (**9**)....53
Figure S48. HMBC (500 MHz, DMSO-d_6) spectrum of 6β,7α,14-trihydroxyconfertifolin (**9**)...54
Figure S49. ROESY (700 MHz, DMSO-d_6) spectrum of 6β,7α,14-trihydroxyconfertifolin (**9**) .55
Figure S50. ^{1}H NMR (500 MHz, MeOD) spectrum of 6β,7α,14-trihydroxyconfertifolin (**9**) 57
Figure S51. ^{13}C NMR (125 MHz, MeOD) spectrum of 6β,7α,14-trihydroxyconfertifolin (**9**) 59
Figure S52. DEPT-135 (125 MHz, MeOD) spectrum of 6β,7α,14-trihydroxyconfertifolin (**9**) ..60
Figure S53. ^{1}H-^{1}H COSY (700 MHz, MeOD) spectrum of 6β,7α,14-trihydroxyconfertifolin (**9**) 61
Figure S54. ECD spectrum of 6β,7α,14-trihydroxyconfertifolin (**9**) in methanol.......................62
Figure S55. ^{1}H NMR (700 MHz, acetone-d_6) spectrum of 7α,14-dihydroxy-6β-p-nitrobenzoylconfertifolin (**10**) 63
Figure S56. ^{13}C NMR (175 MHz, acetone-d_6) spectrum of 7α,14-dihydroxy-6β-p-nitrobenzoylconfertifolin (**10**) 65
Figure S57. ECD spectrum of 7α,14-dihydroxy-6β-p-nitrobenzoylconfertifolin (**10**) in methanol 66

Figure S1. ^{1}H NMR (500 MHz, $CDCl_3$) spectrum of aspilactonol F (**1**)

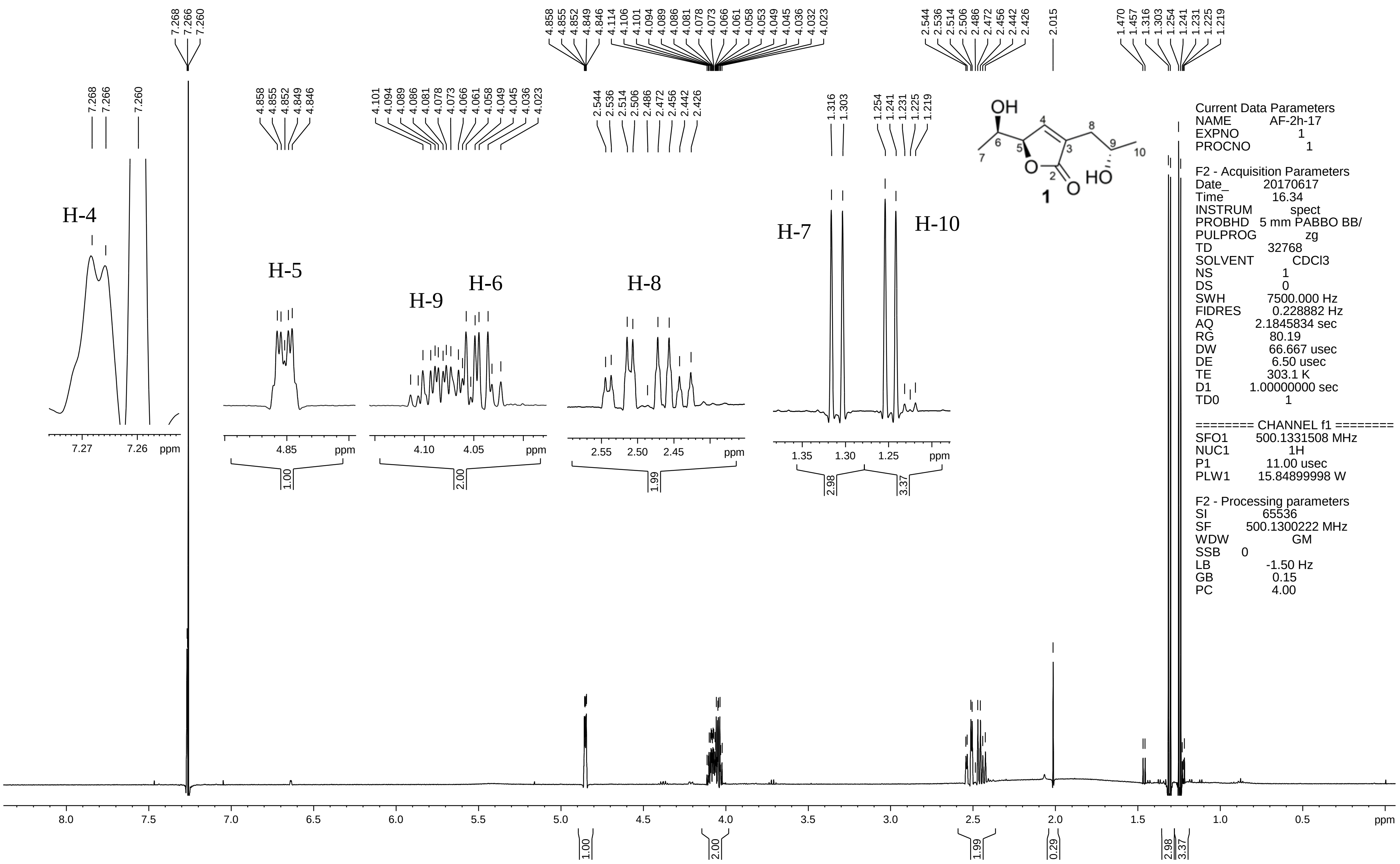

Figure S2. ^{13}C NMR (125 MHz, $CDCl_3$) spectrum of aspilactonol F (**1**)

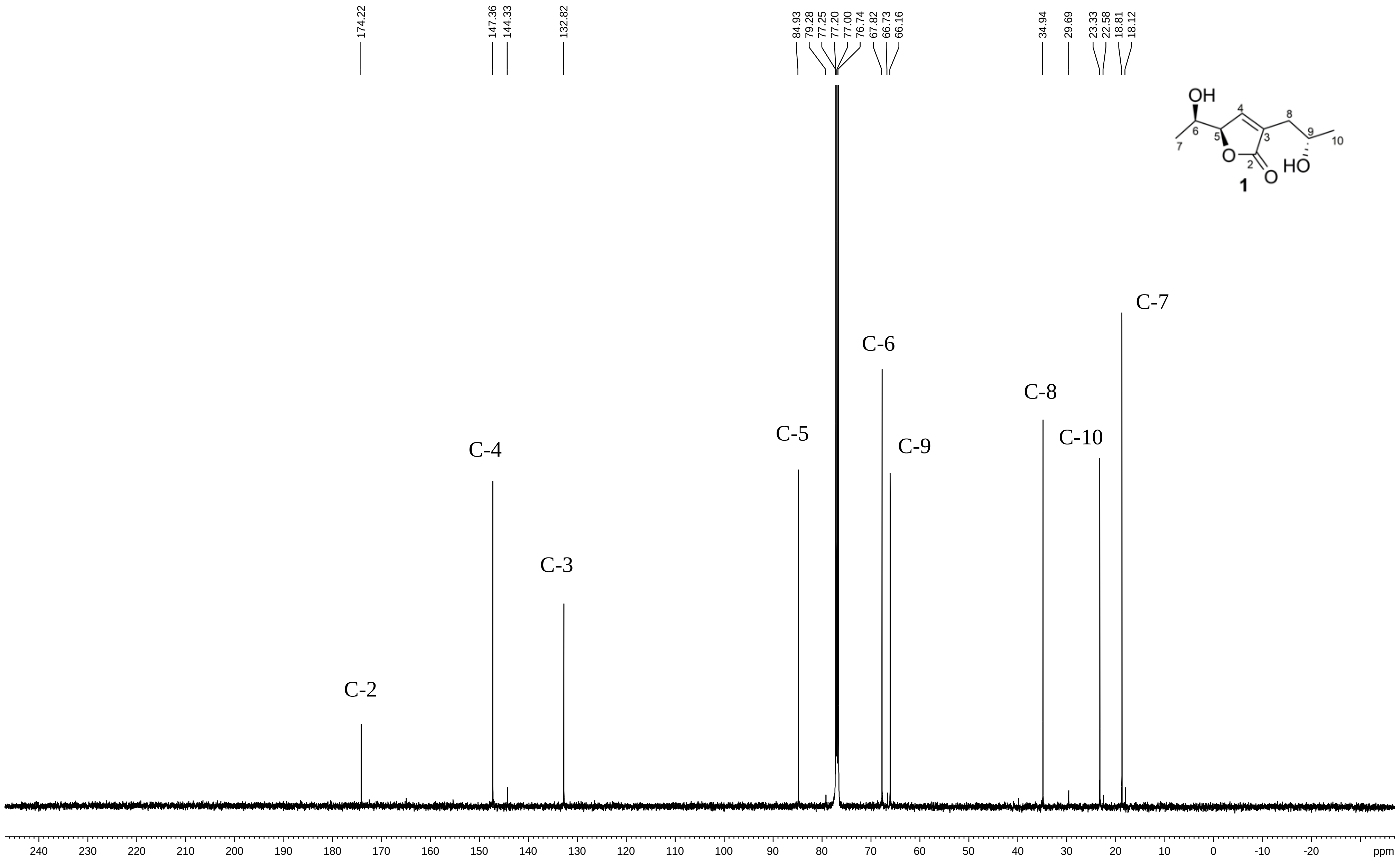

Figure S3. DEPT-135 (125 MHz, $CDCl_3$) spectrum of aspilactonol F (**1**)

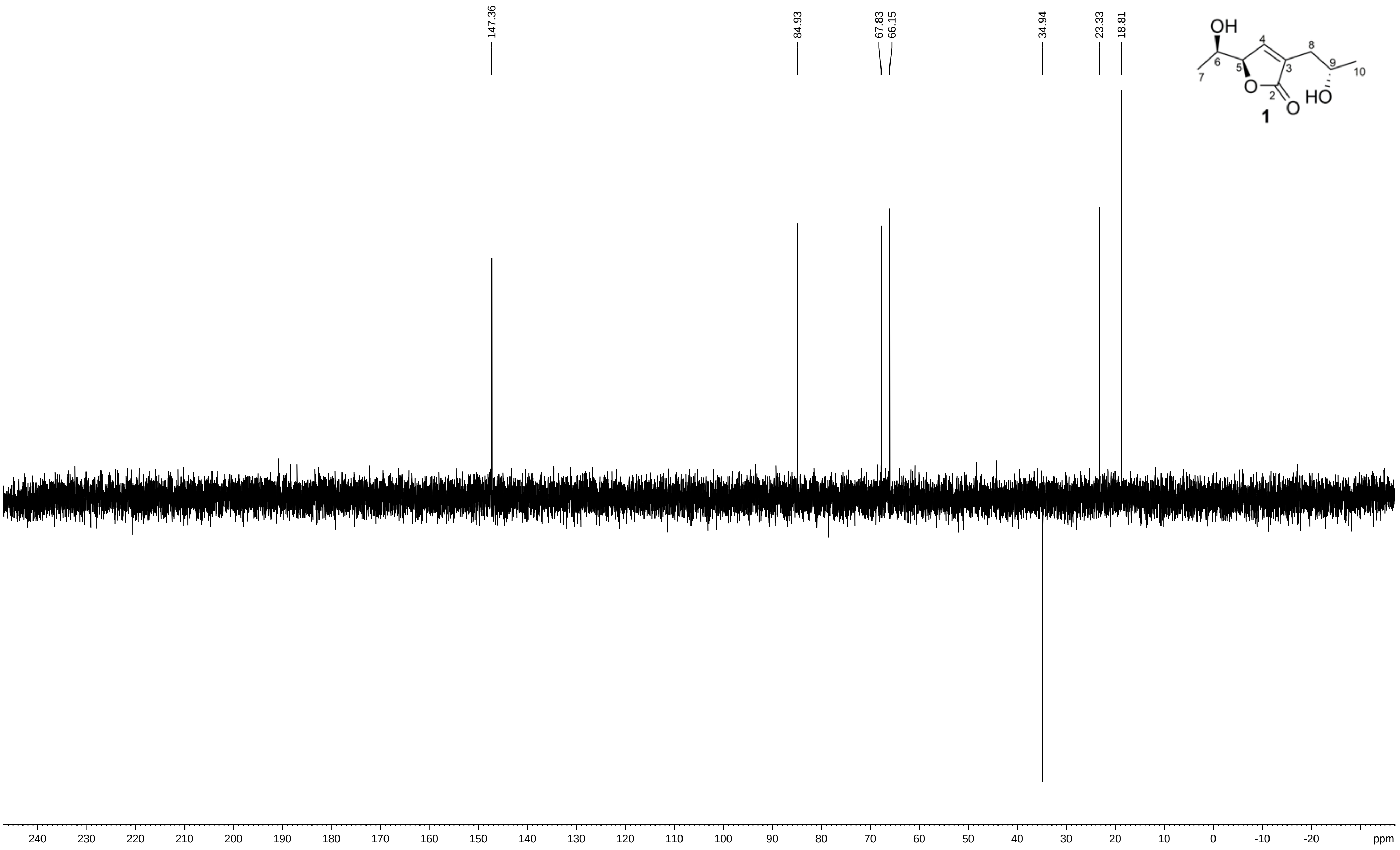

Figure S4. ^1H-^1H COSY (500 MHz, $CDCl_3$) spectrum of aspilactonol F (**1**)

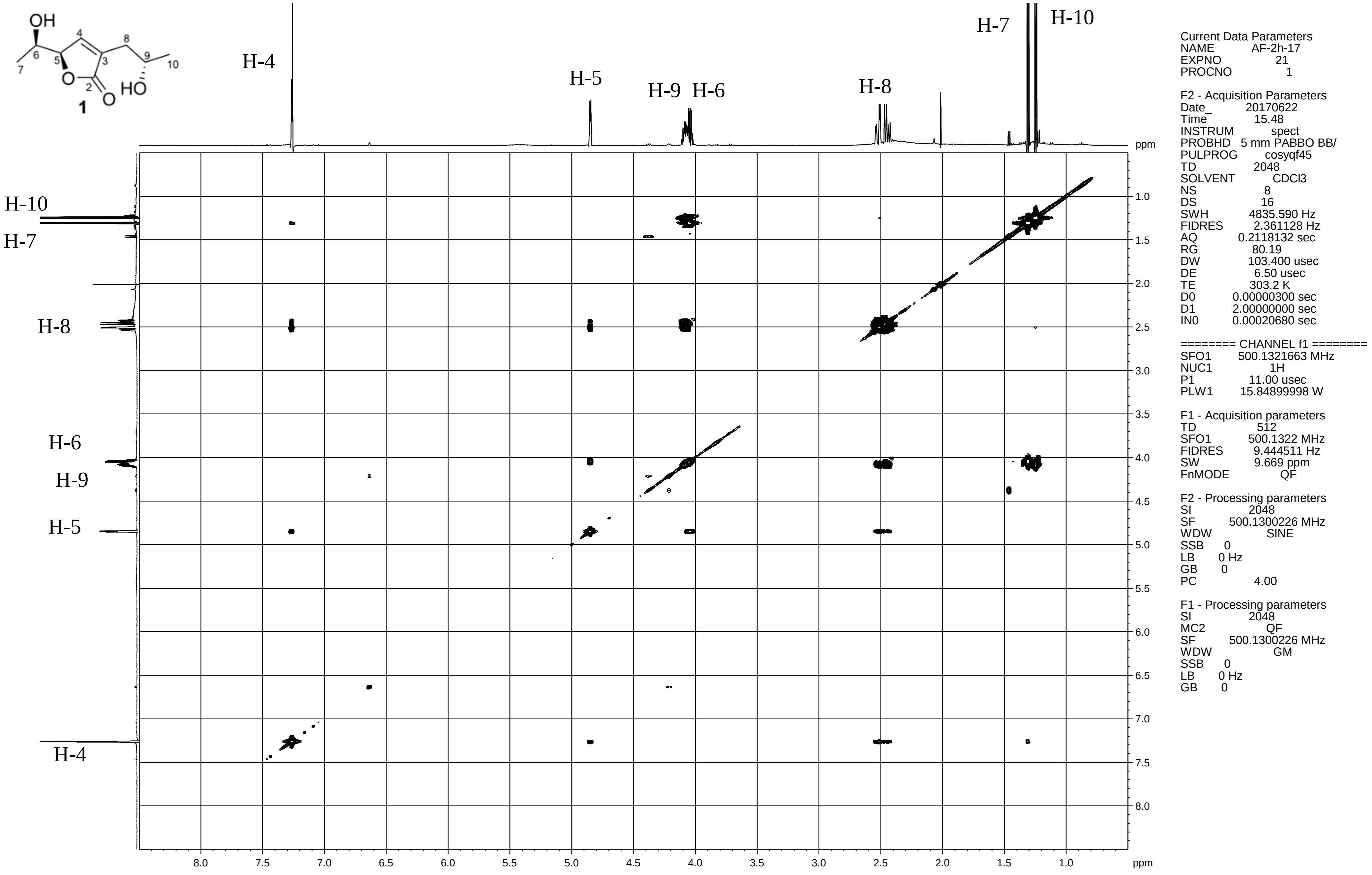

Figure S5. HSQC (500 MHz, $CDCl_3$) spectrum of aspilactonol F (**1**)

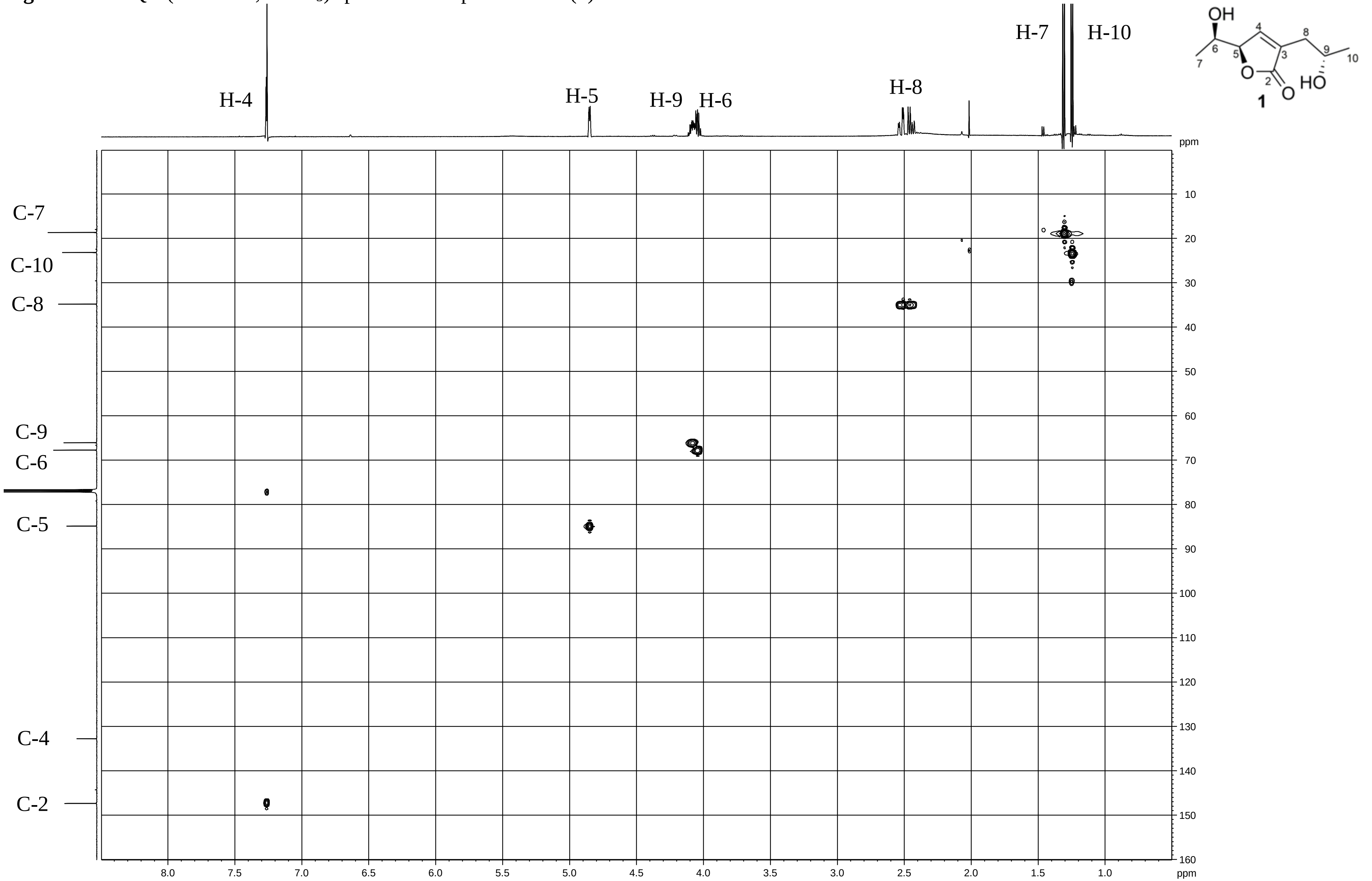

Figure S6. HMBC (500 MHz, $CDCl_3$) spectrum of aspilactonol F (**1**)

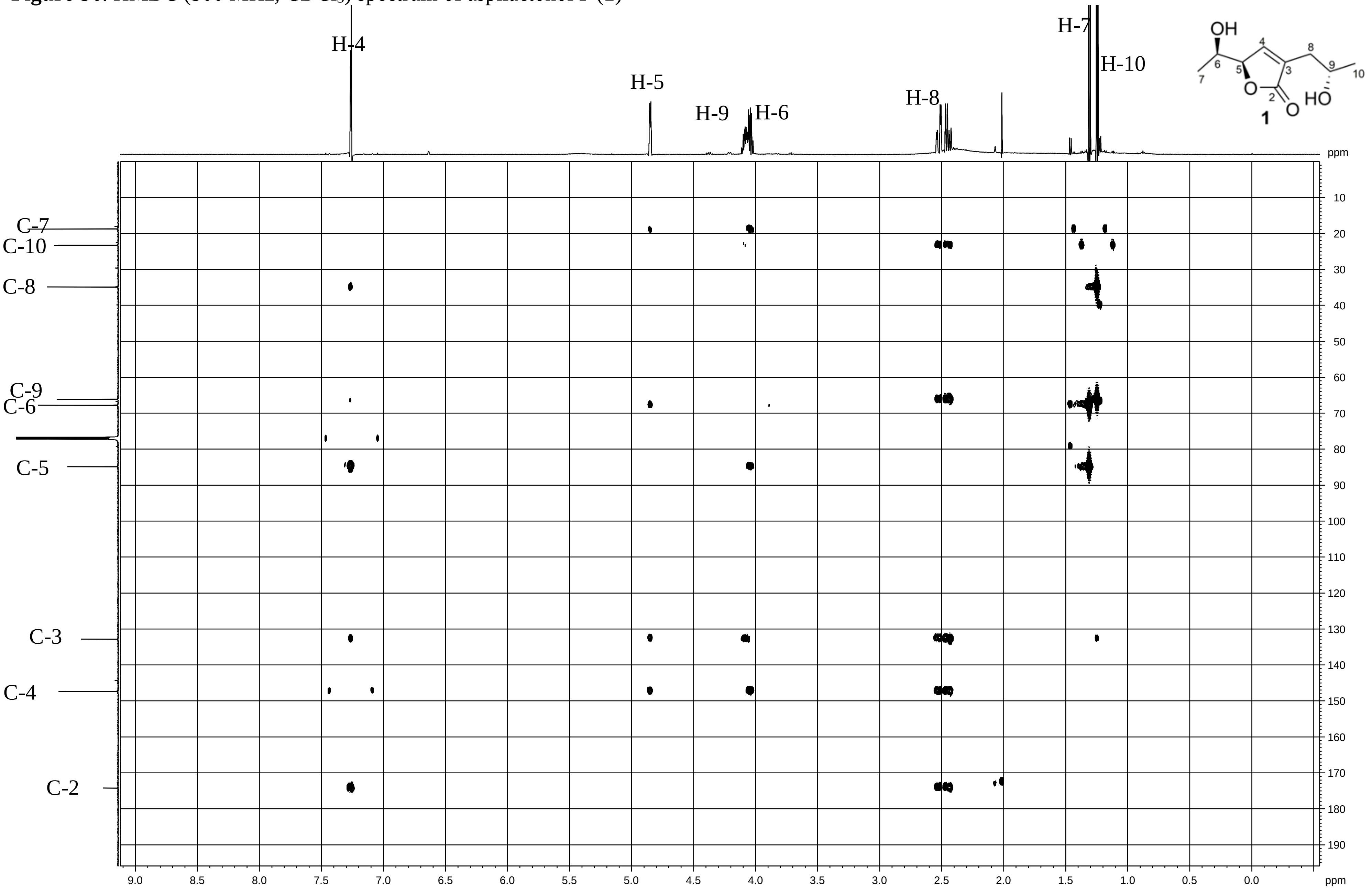

Figure S7. ROESY (500 MHz, $CDCl_3$) spectrum of aspilactonol F (**1**)

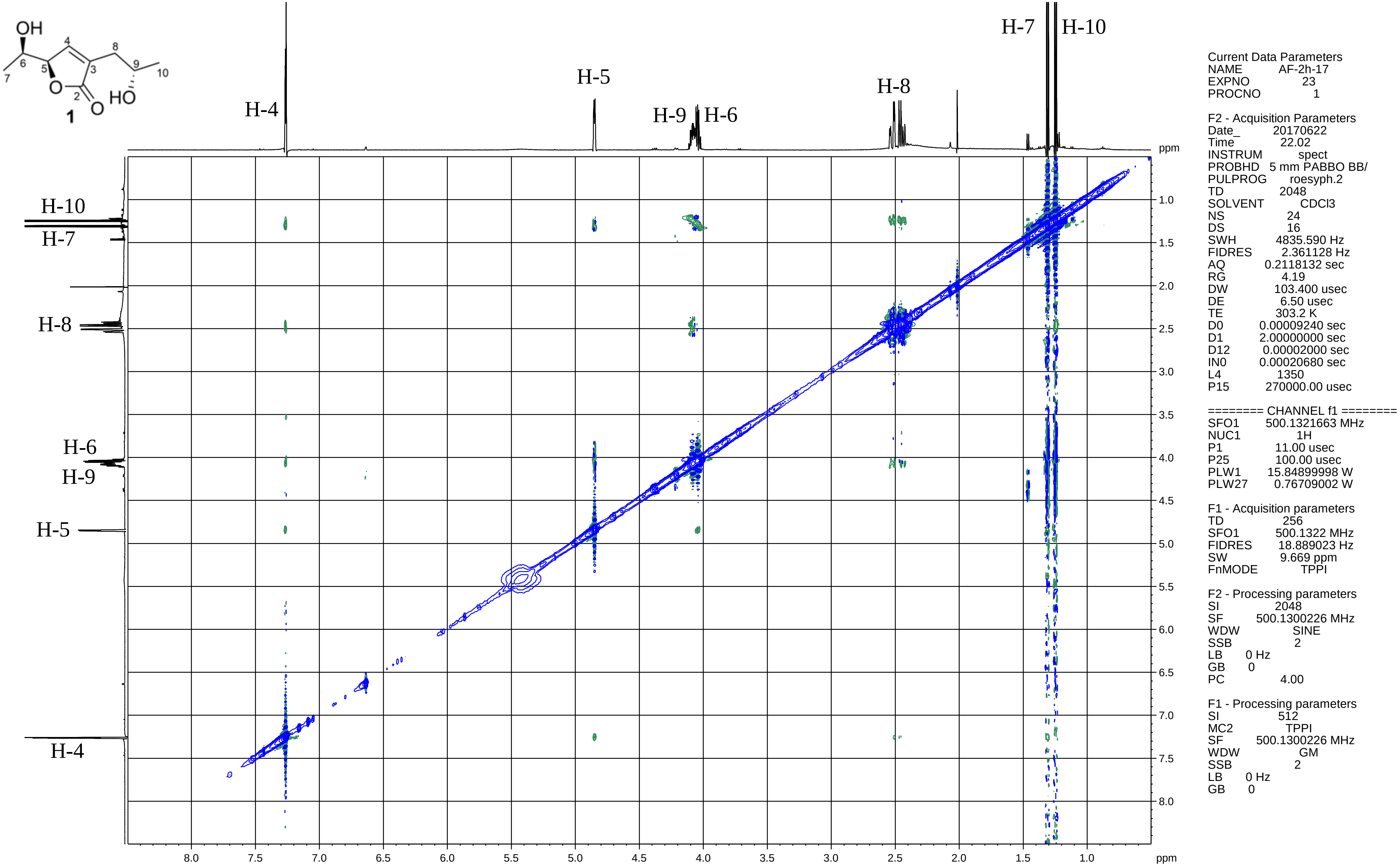

Figure S8. ECD spectrum of aspilactonol F (**1**) in methanol

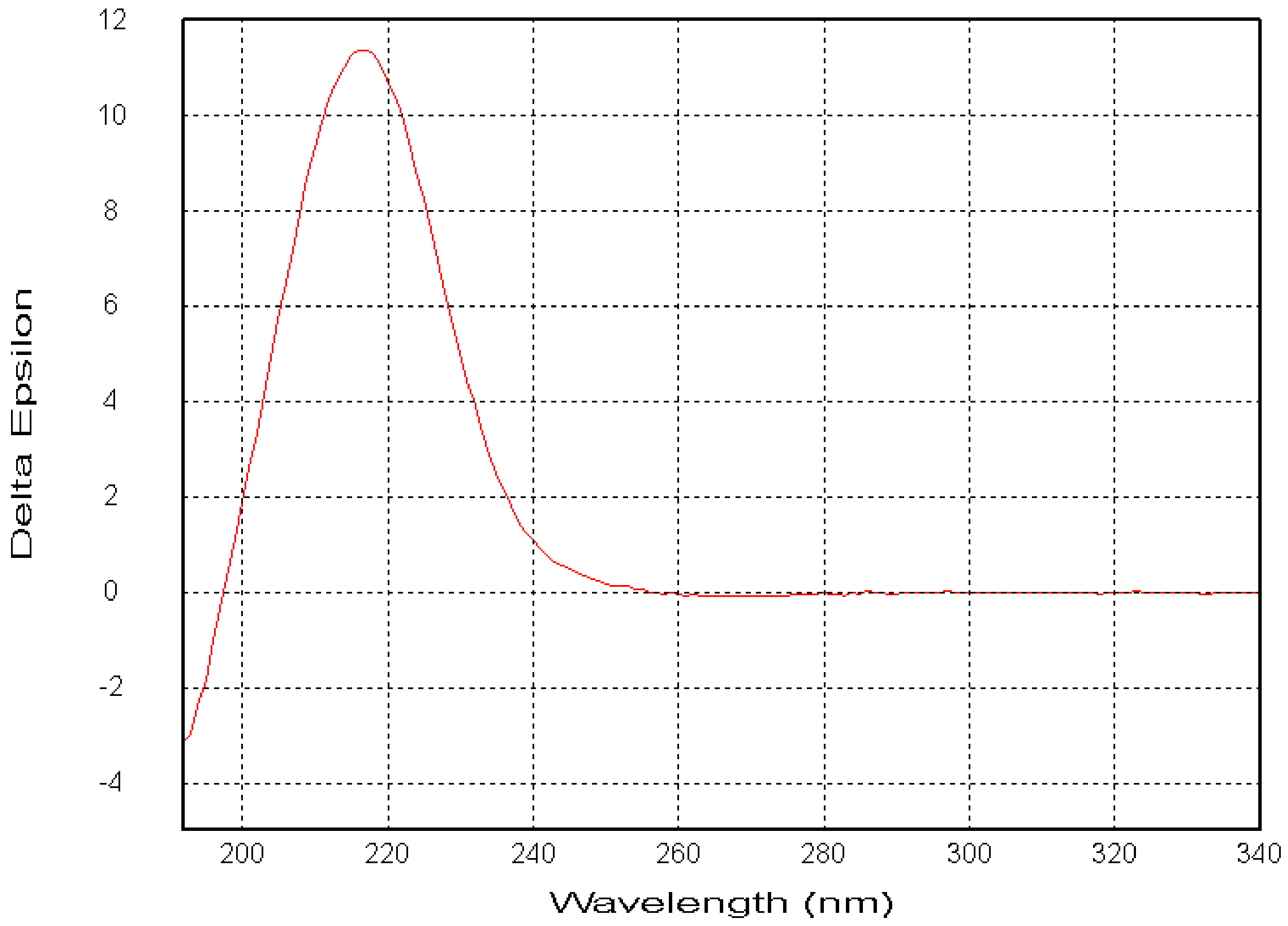

Figure S9. ^{1}H NMR (500 MHz, $CDCl_3$) spectrum of aspilactonol G (**2**)

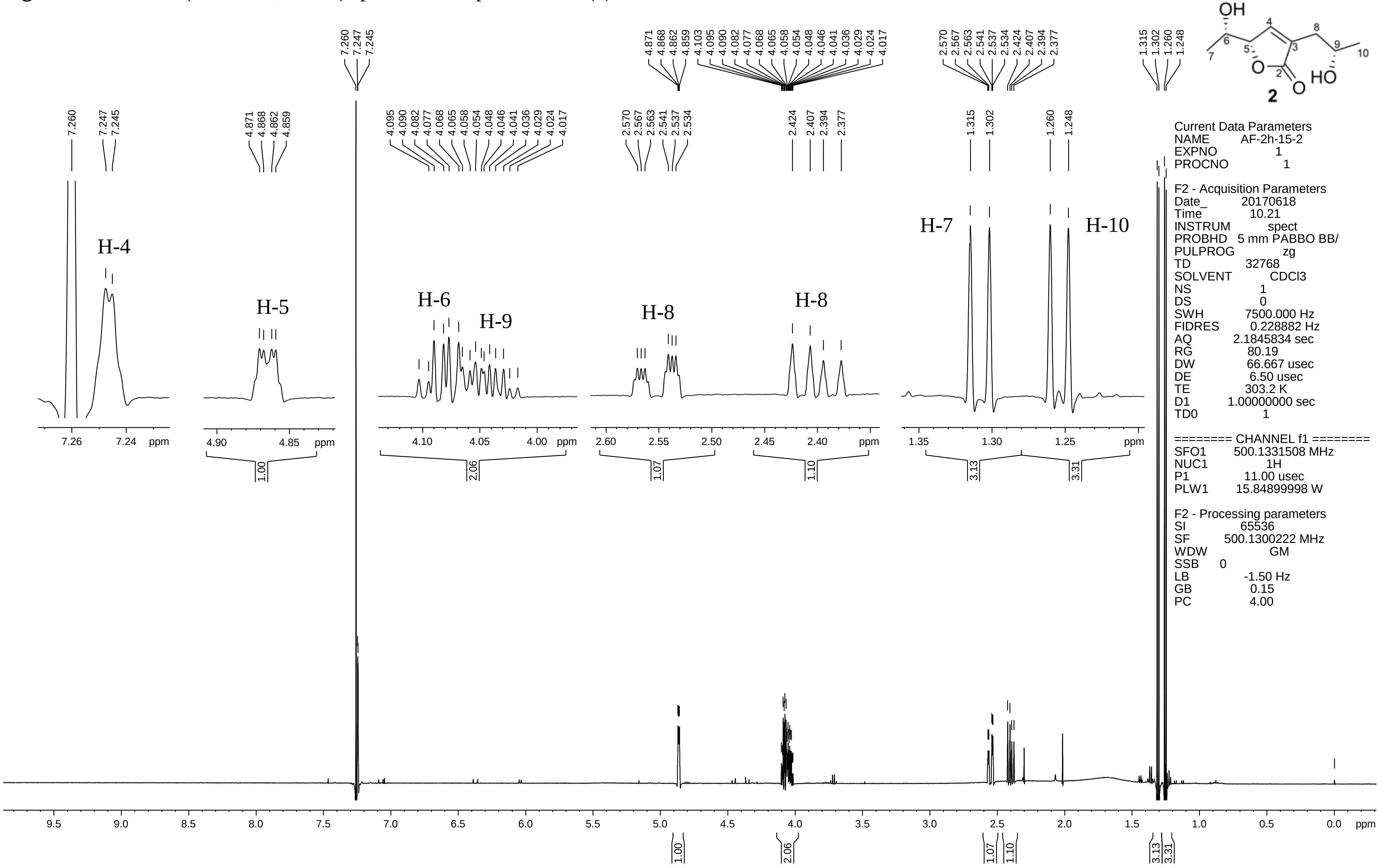

Figure S10. ^{13}C NMR (125 MHz, $CDCl_3$) spectrum of aspilactonol G (**2**)

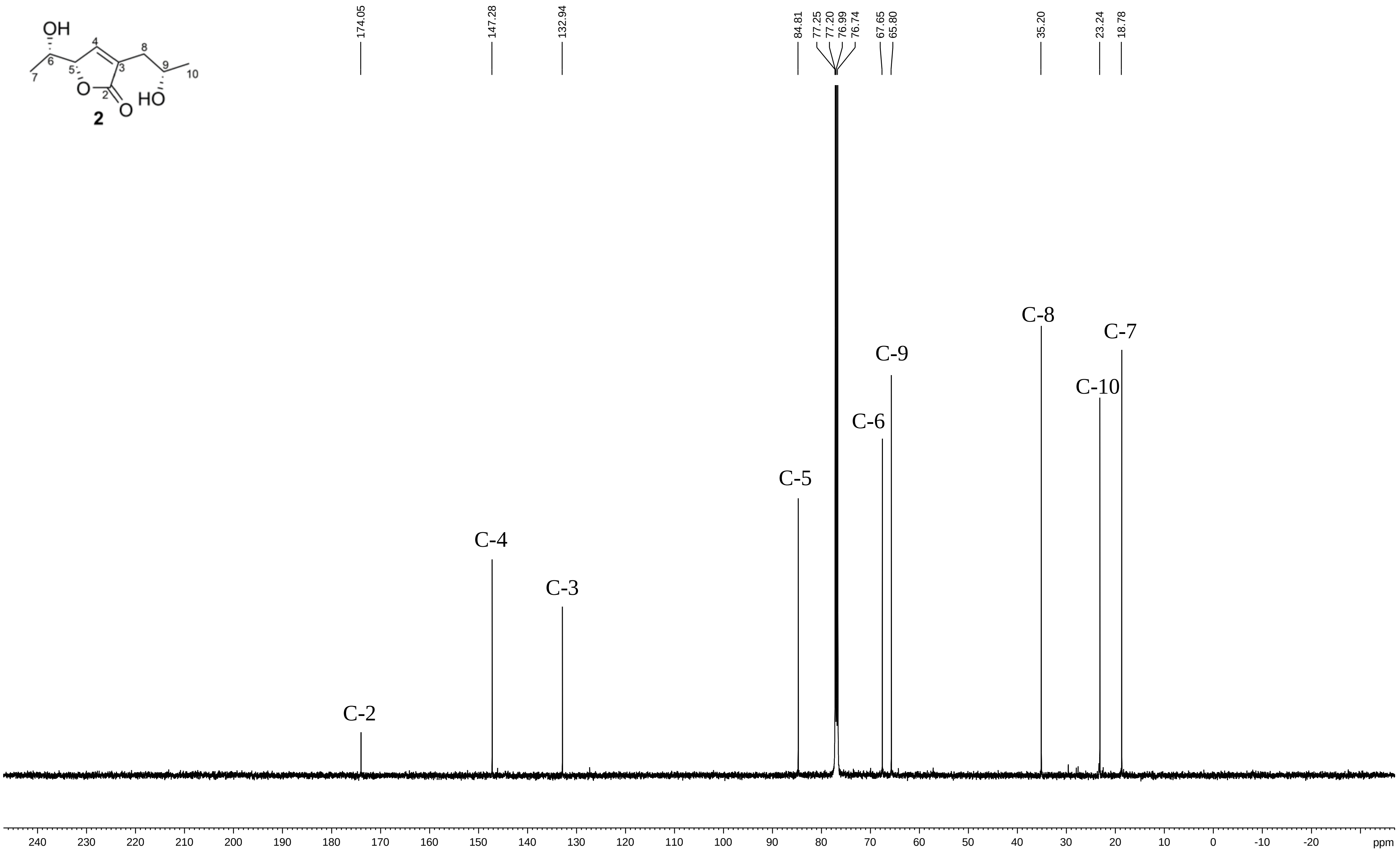

Figure S11. DEPT-135 (125 MHz, $CDCl_3$) spectrum of aspilactonol G (**2**)

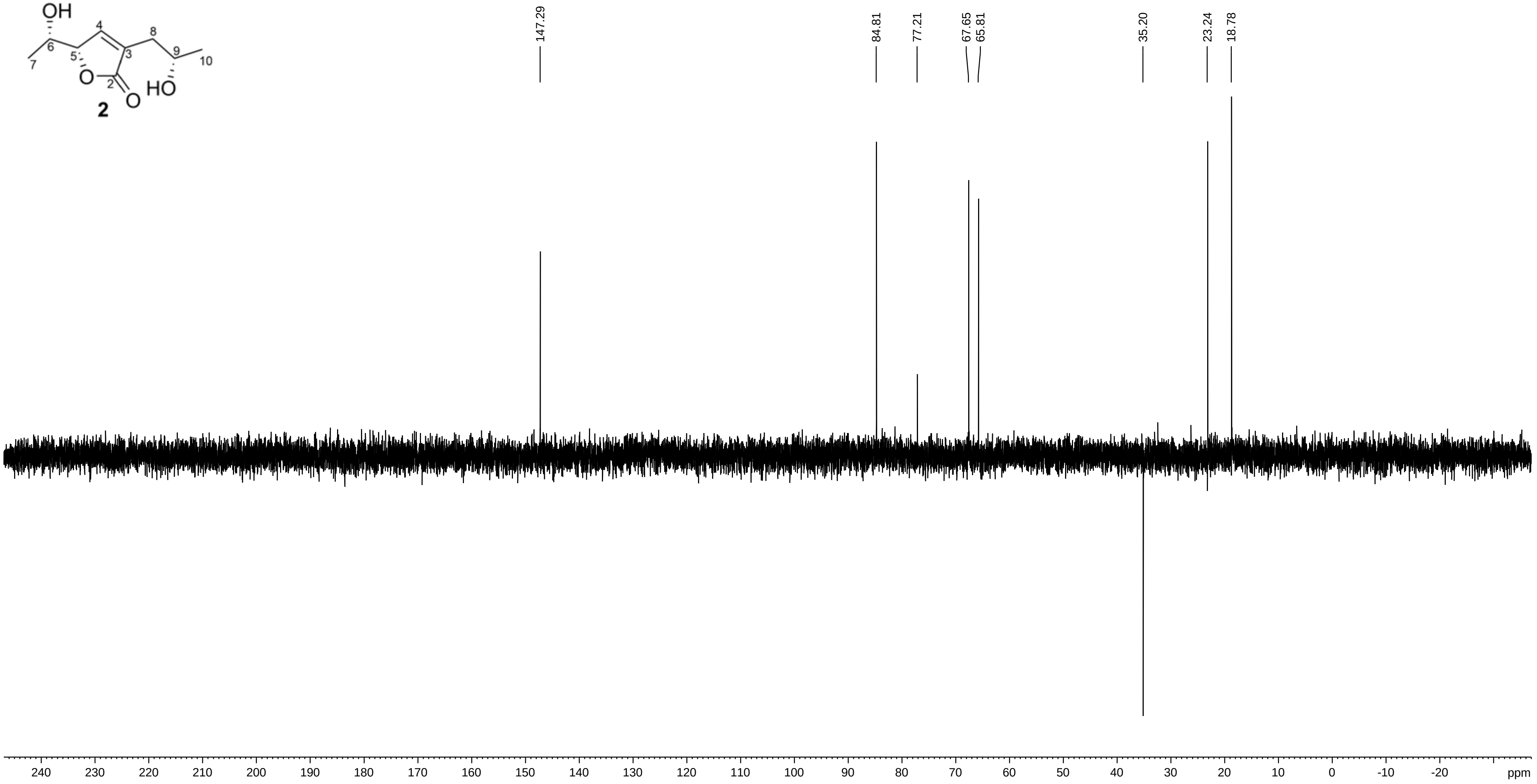

Figure S12. ^{1}H NMR (700 MHz, acetone-d_6) spectrum of aspilactonol G (**2**)

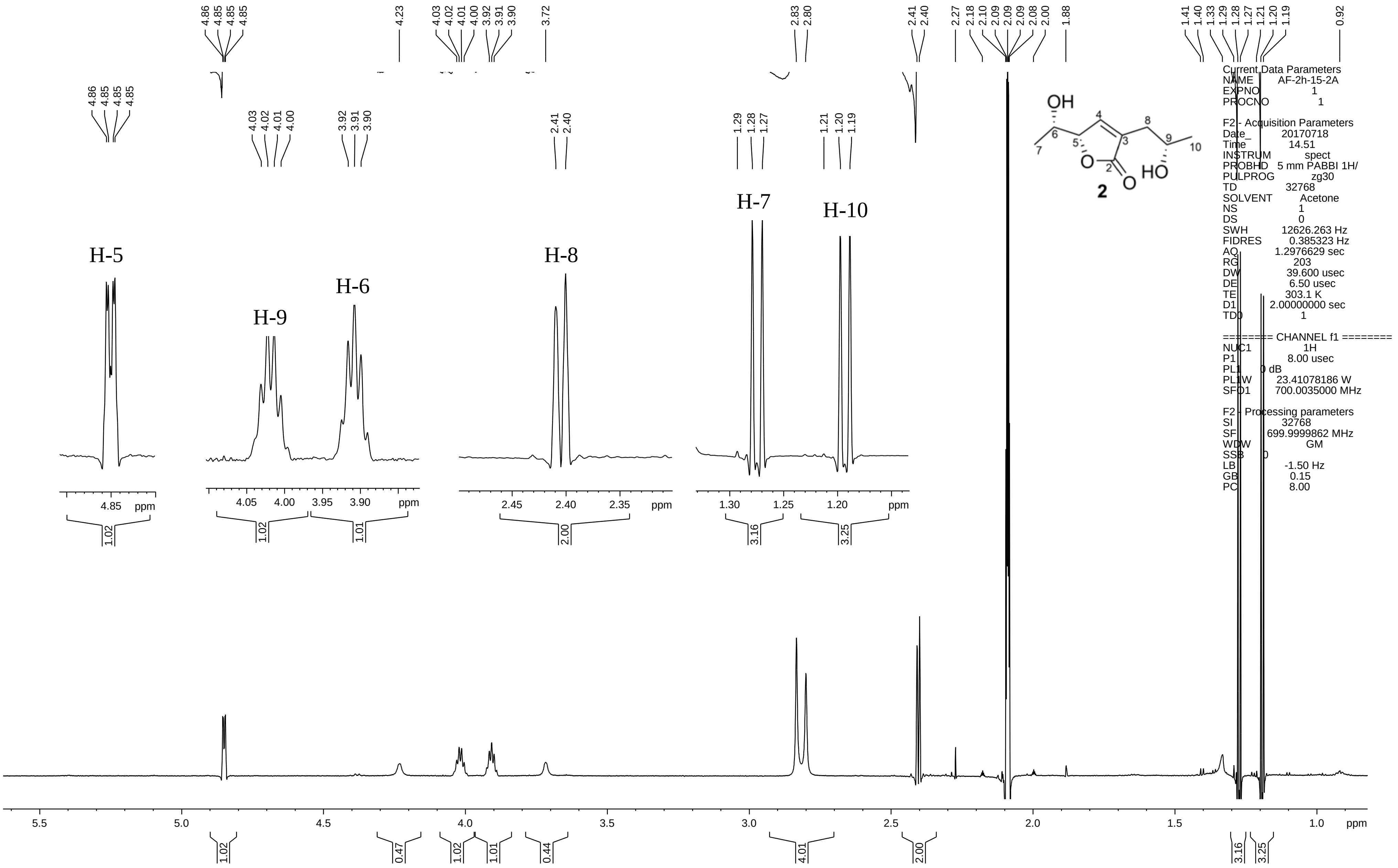

Figure S13. ^{13}C NMR (125 MHz, acetone-d$_6$) spectrum of aspilactonol G (**2**)

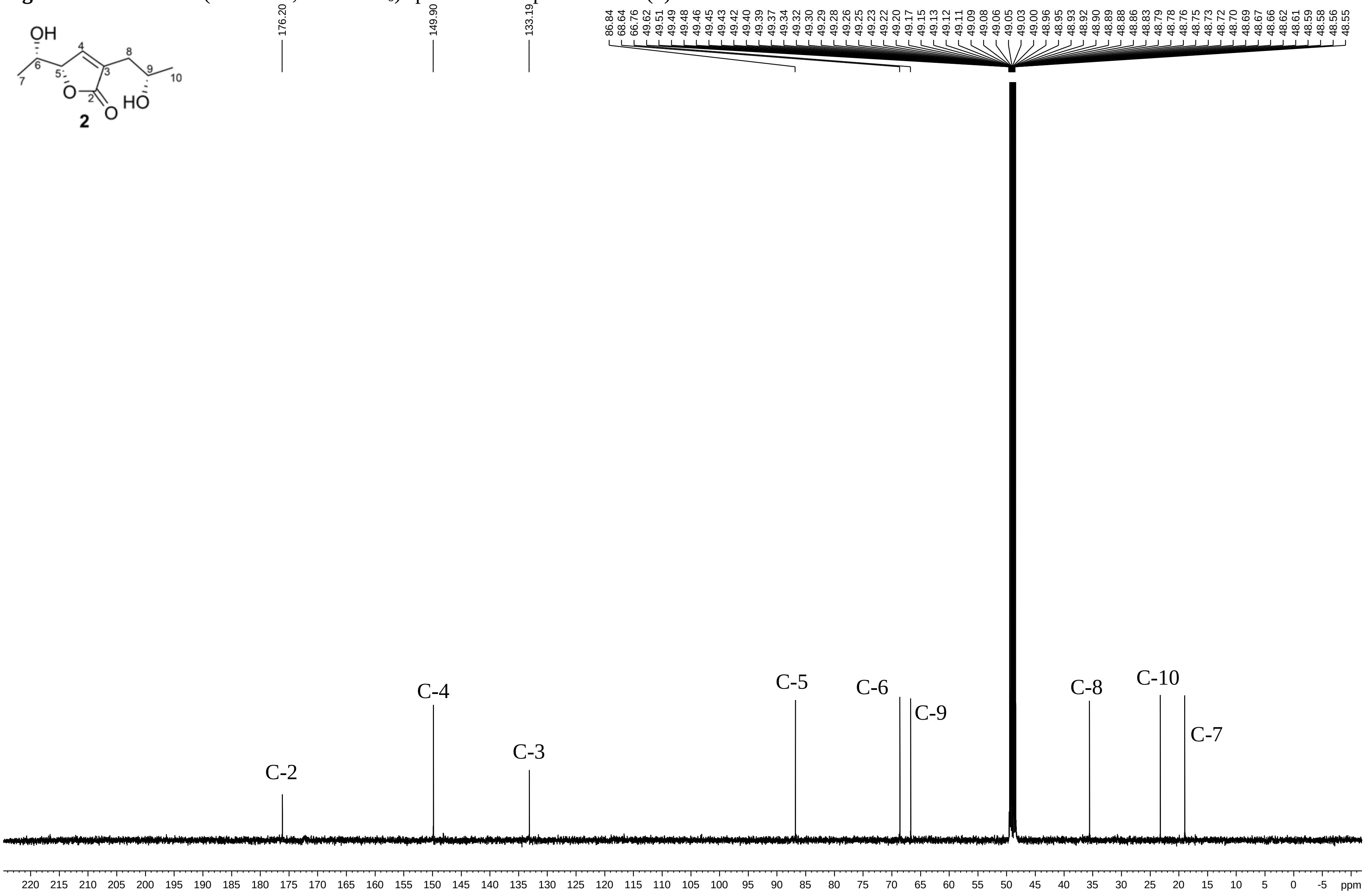

Figure S14. ^{1}H-^{1}H COSY (700 MHz, acetone-d_6) spectrum of aspilactonol G (**2**)

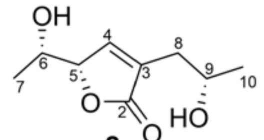

2

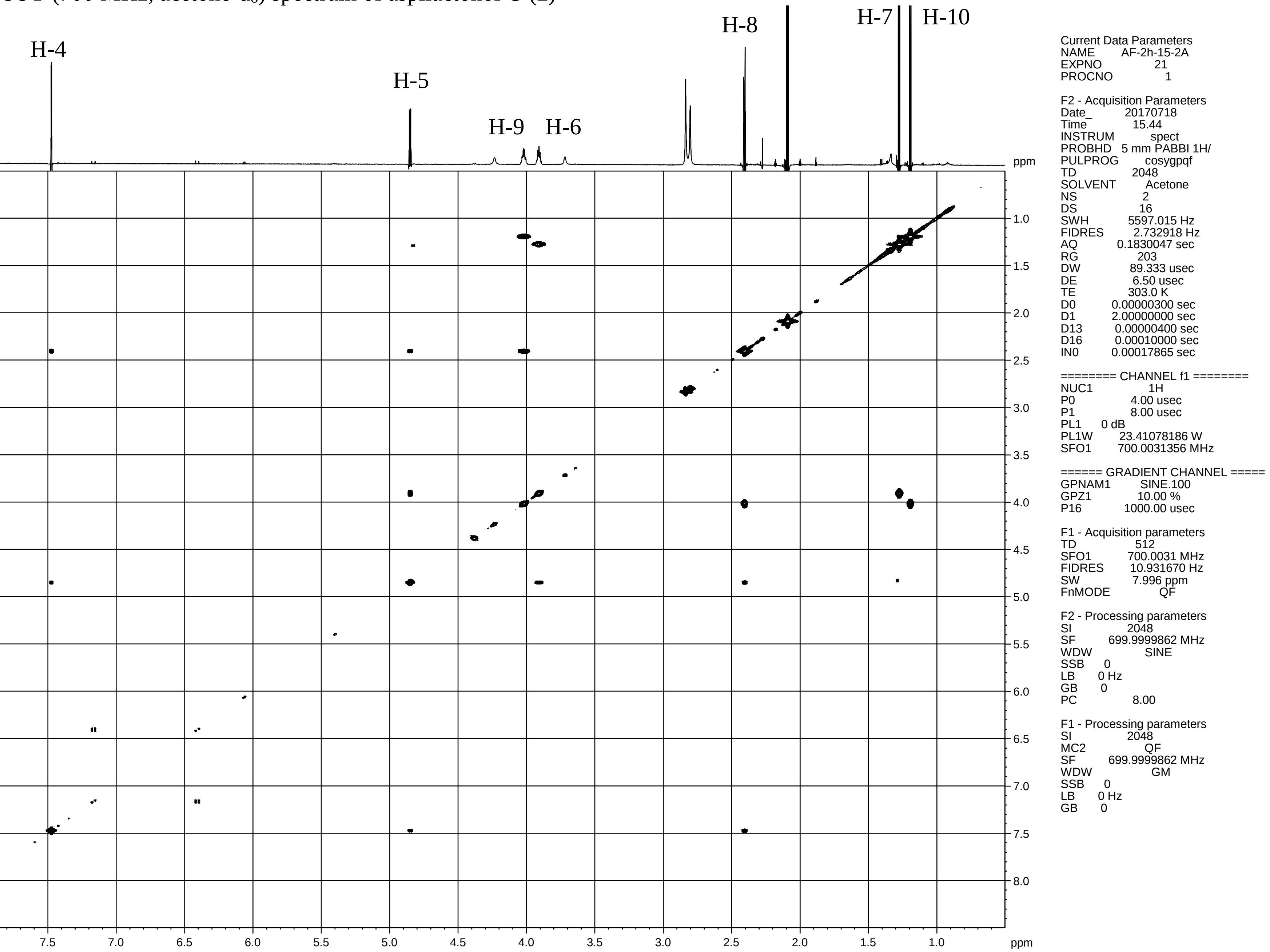

Figure S15. HSQC (700 MHz, acetone-d_6) spectrum of aspilactonol G (**2**)

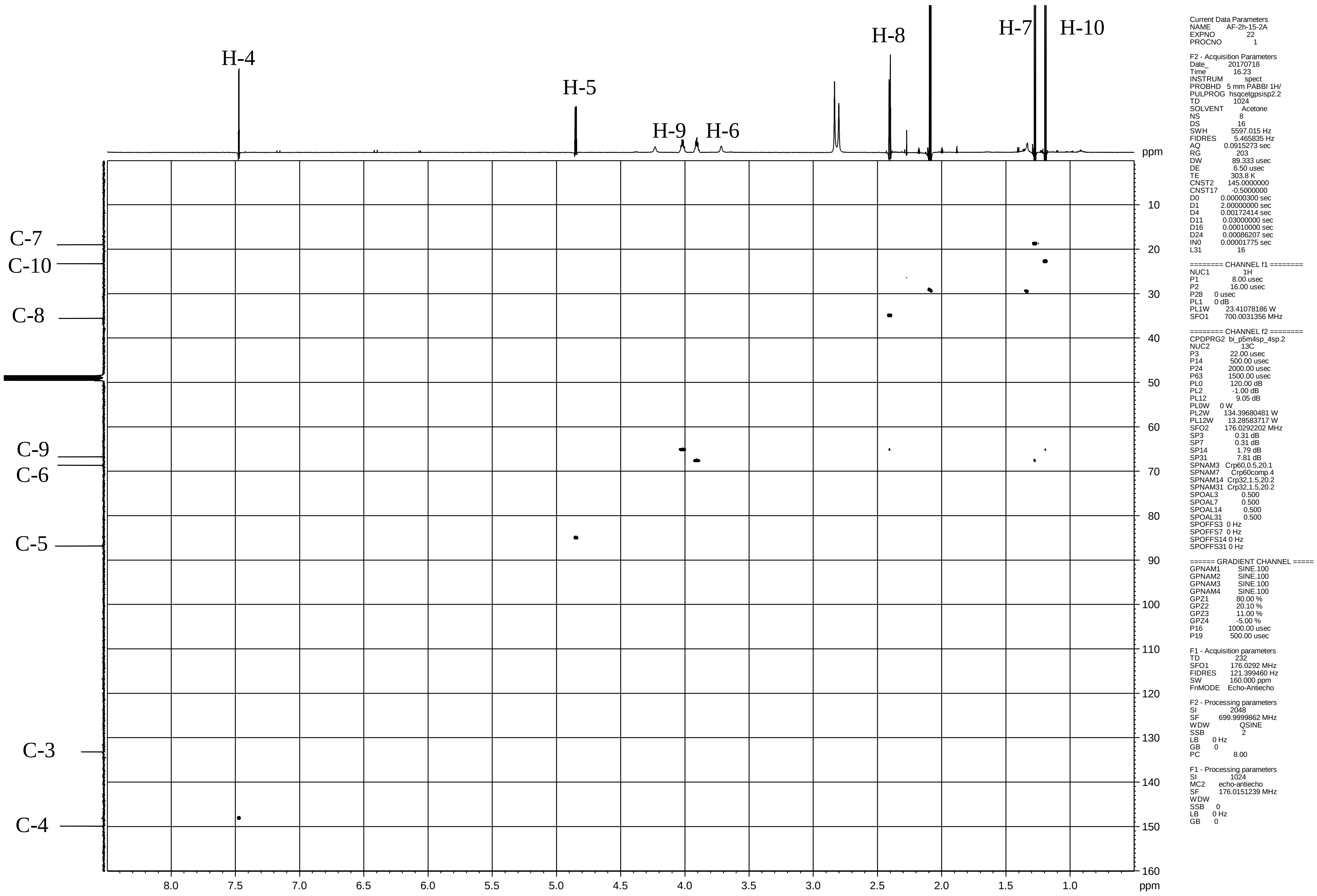

Figure S16. HMBC (700 MHz, acetone-d_6) spectrum of aspilactonol G (**2**)

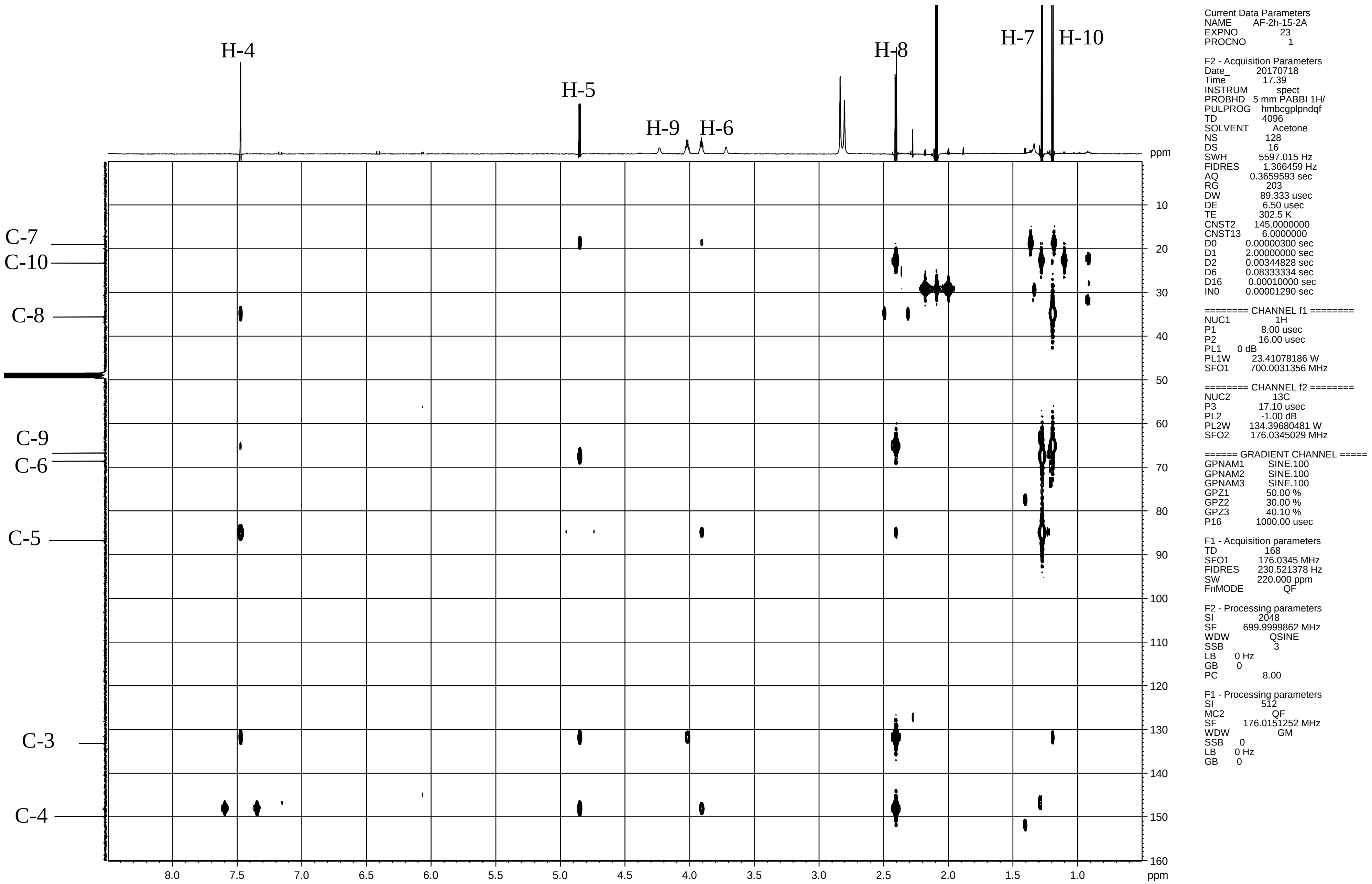

Figure S17. ECD spectrum of aspilactonol G (**2**) in methanol

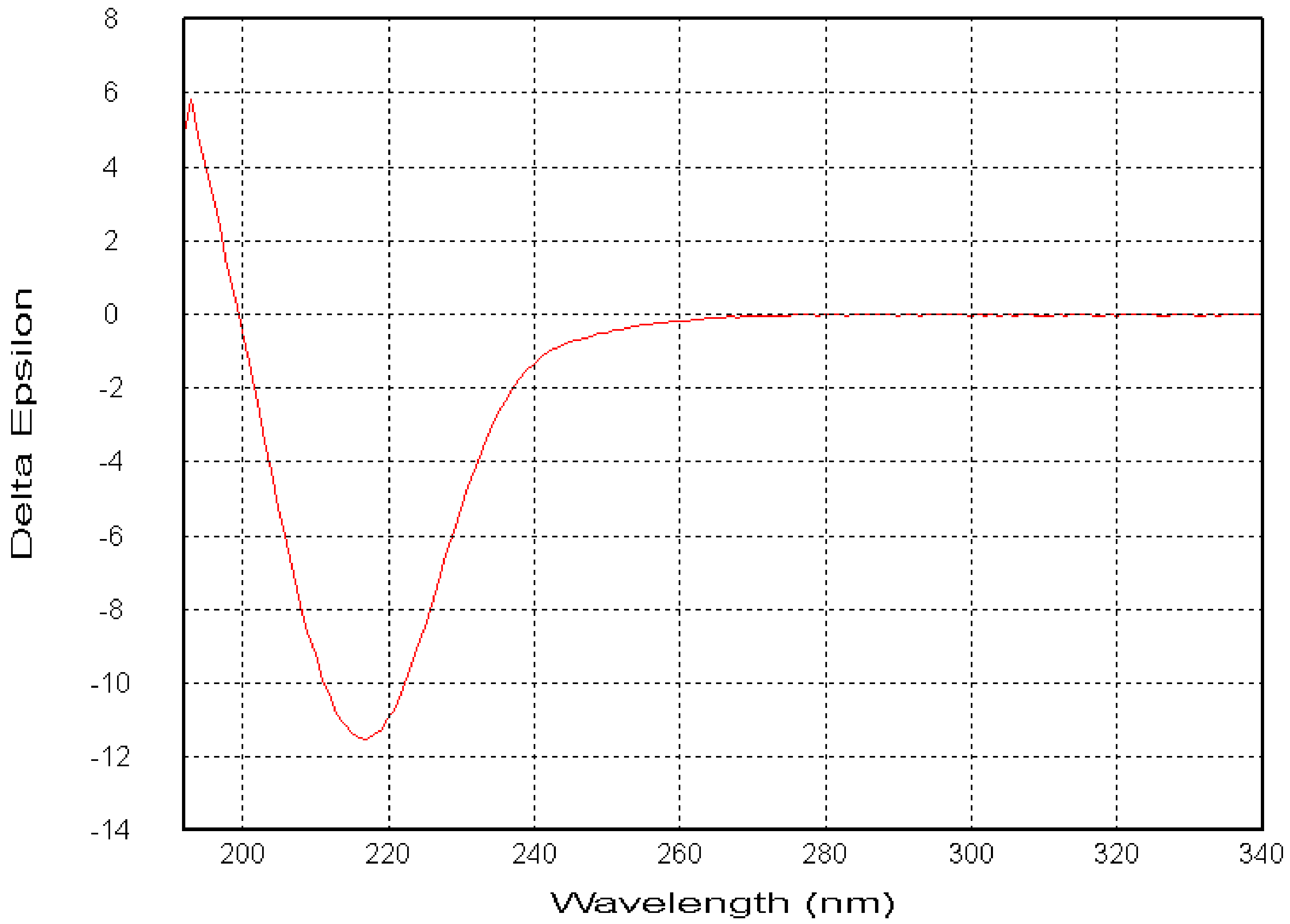

Figure S18. ^{1}H NMR (500 MHz, $CDCl_3$) spectrum of dihydroaspirone (**3**)

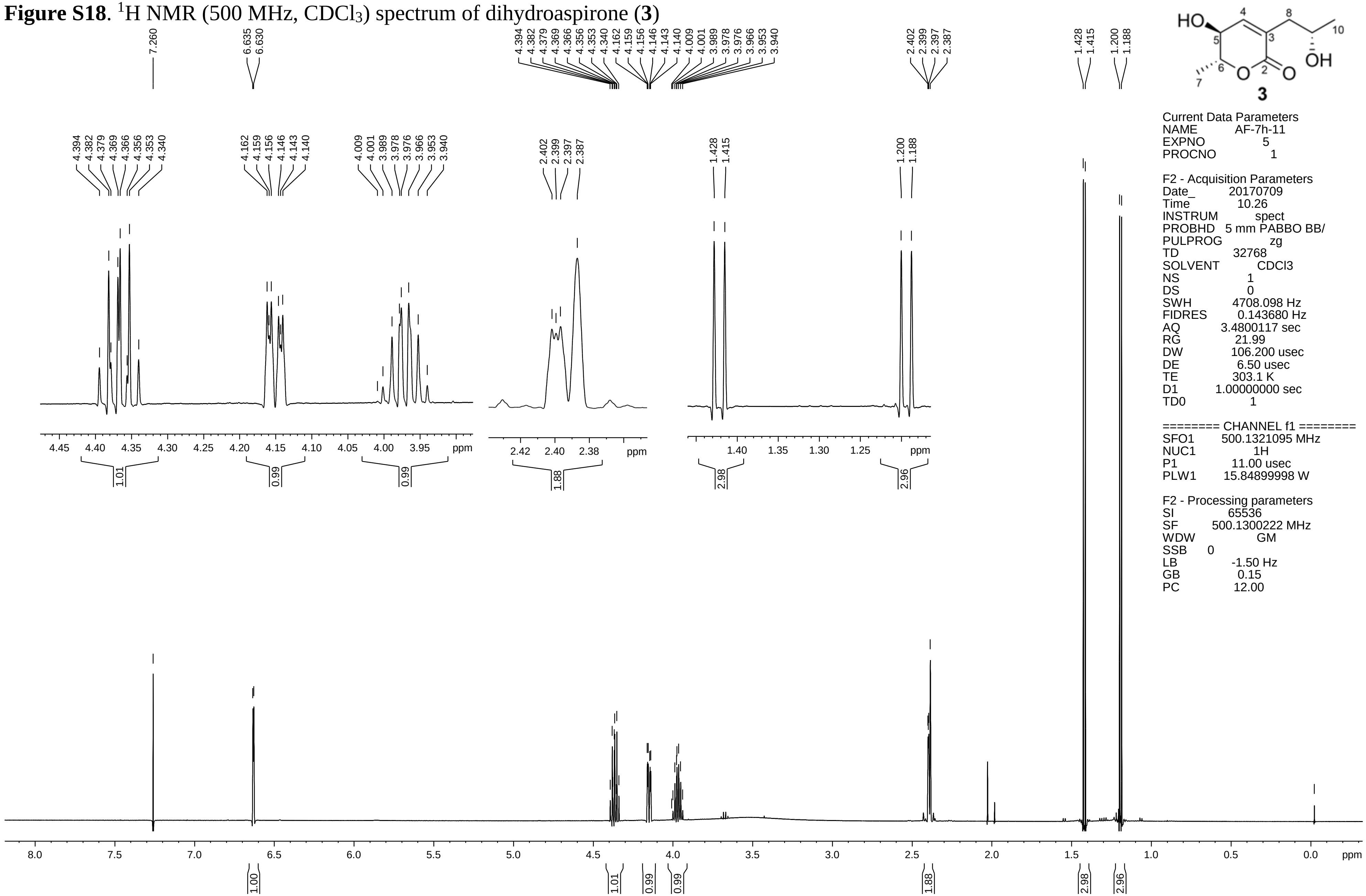

Figure S19. ^{13}C NMR (176 MHz, $CDCl_3$) spectrum of dihydroaspirone (**3**)

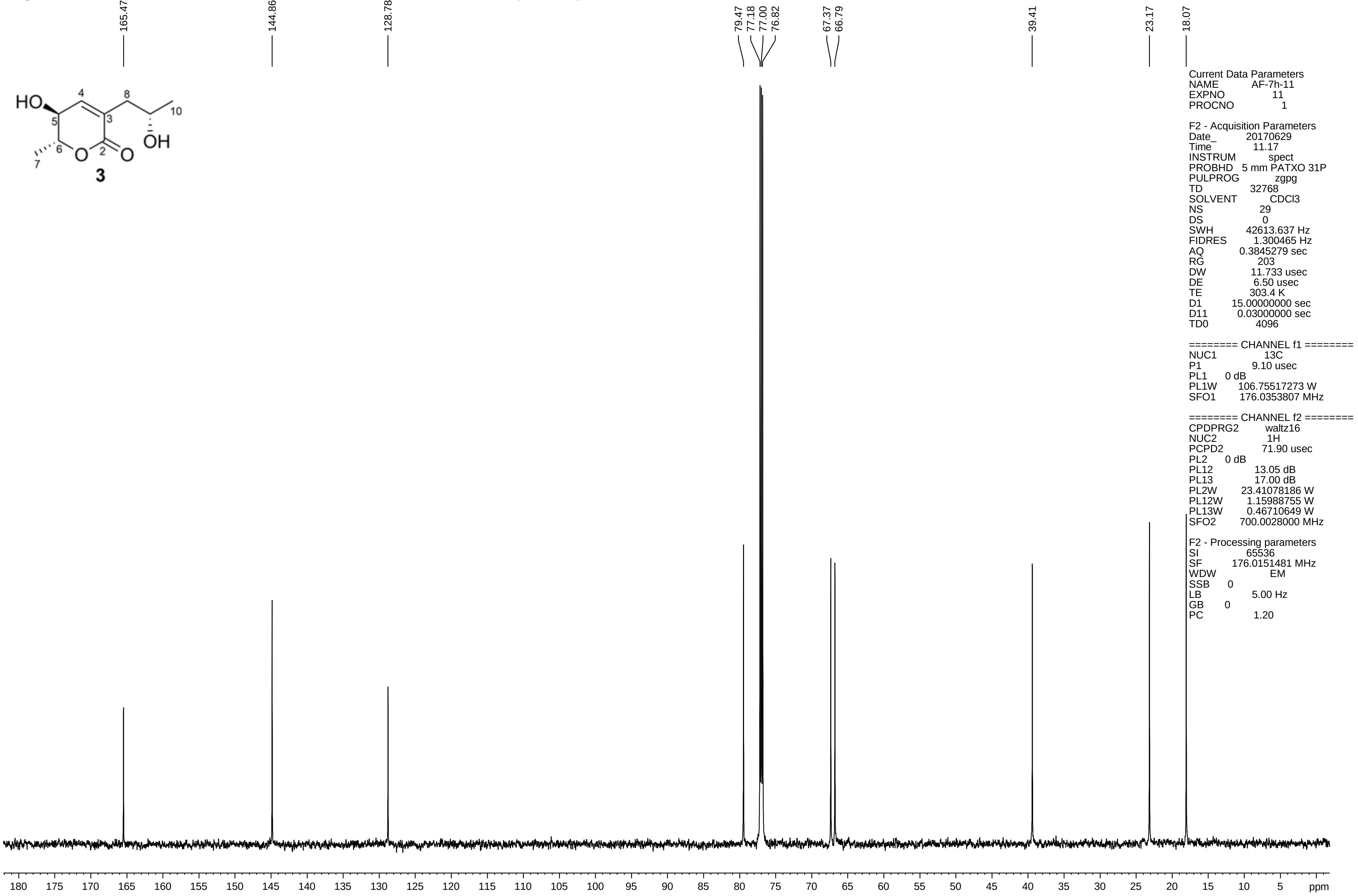

Figure S20. ^{1}H NMR (500 MHz, $CDCl_3$) spectrum of 12-*epi*-aspertetranone D (**4**)

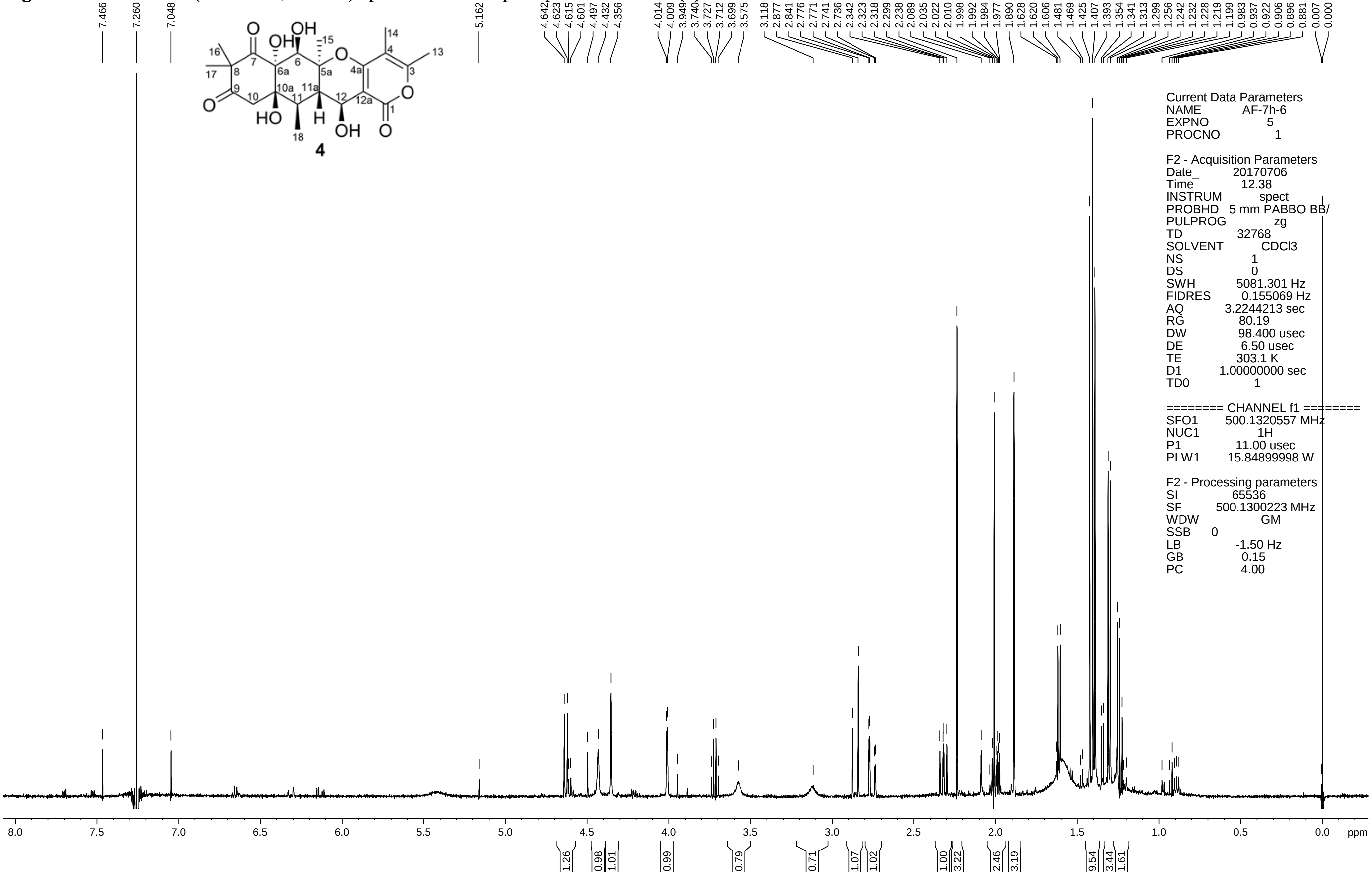

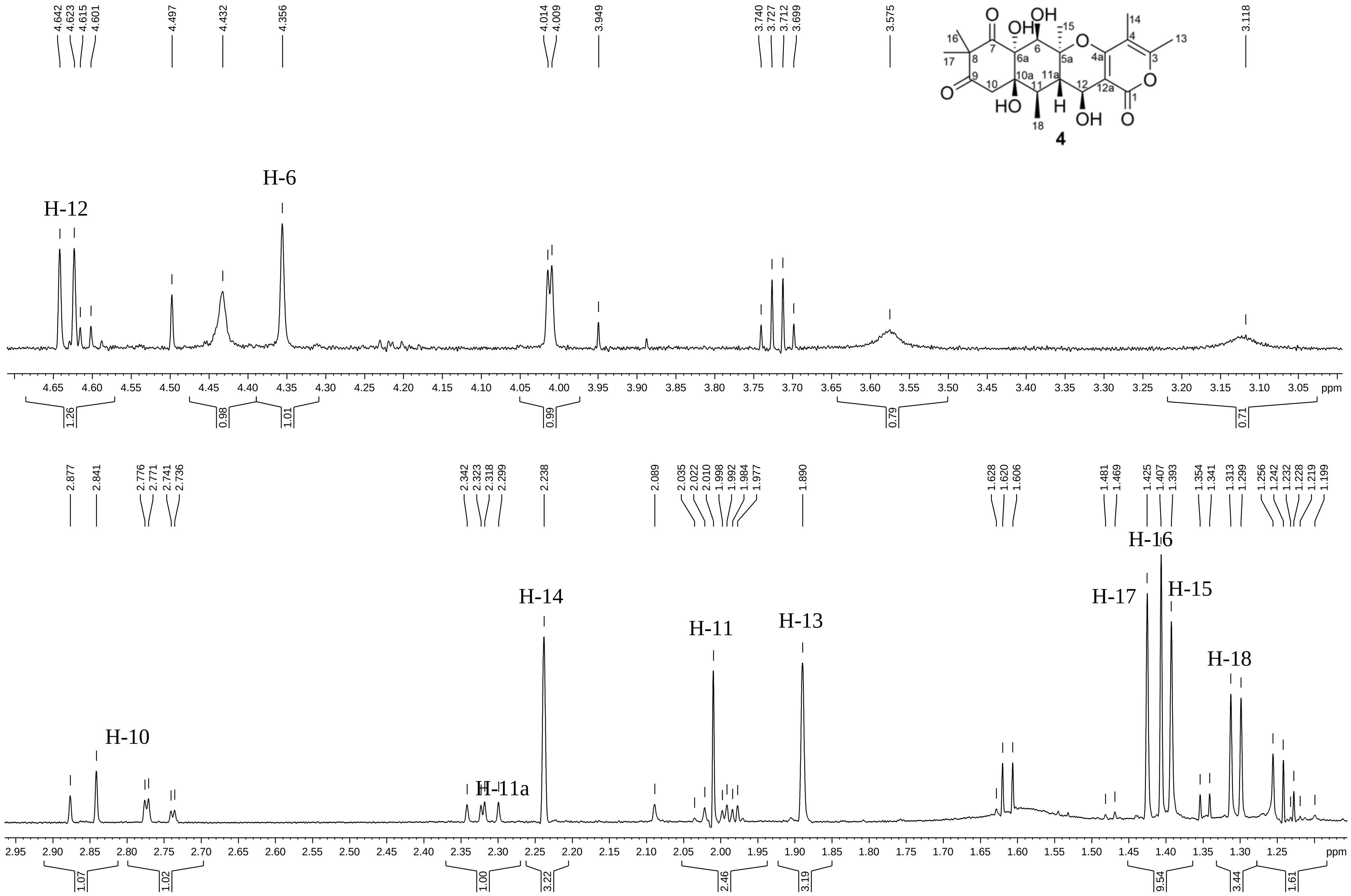
4.642 4.623 4.615 4.601 4.497 4.432 4.356 4.014 4.009 3.949 3.740 3.727 3.712 3.699 3.575 3.118
H-12
H-6
4
ppm
1.26 0.98 1.01 0.99 0.79 0.71
2.877 2.841 2.776 2.771 2.741 2.736 2.342 2.323 2.318 2.299 2.238 2.089 2.035 2.022 2.010 1.998 1.992 1.984 1.977 1.890 1.628 1.620 1.606 1.481 1.469 1.425 1.407 1.393 1.354 1.341 1.313 1.299 1.256 1.242 1.232 1.228 1.219 1.199
H-10
H-11a
H-14
H-11
H-13
H-17
H-16
H-15
H-18
ppm
1.07 1.02 1.00 3.22 2.46 3.19 9.54 3.44 1.61

Figure S21. ^{13}C NMR (176 MHz, $CDCl_3$) spectrum of 12-*epi*-aspertetranone D (**4**)

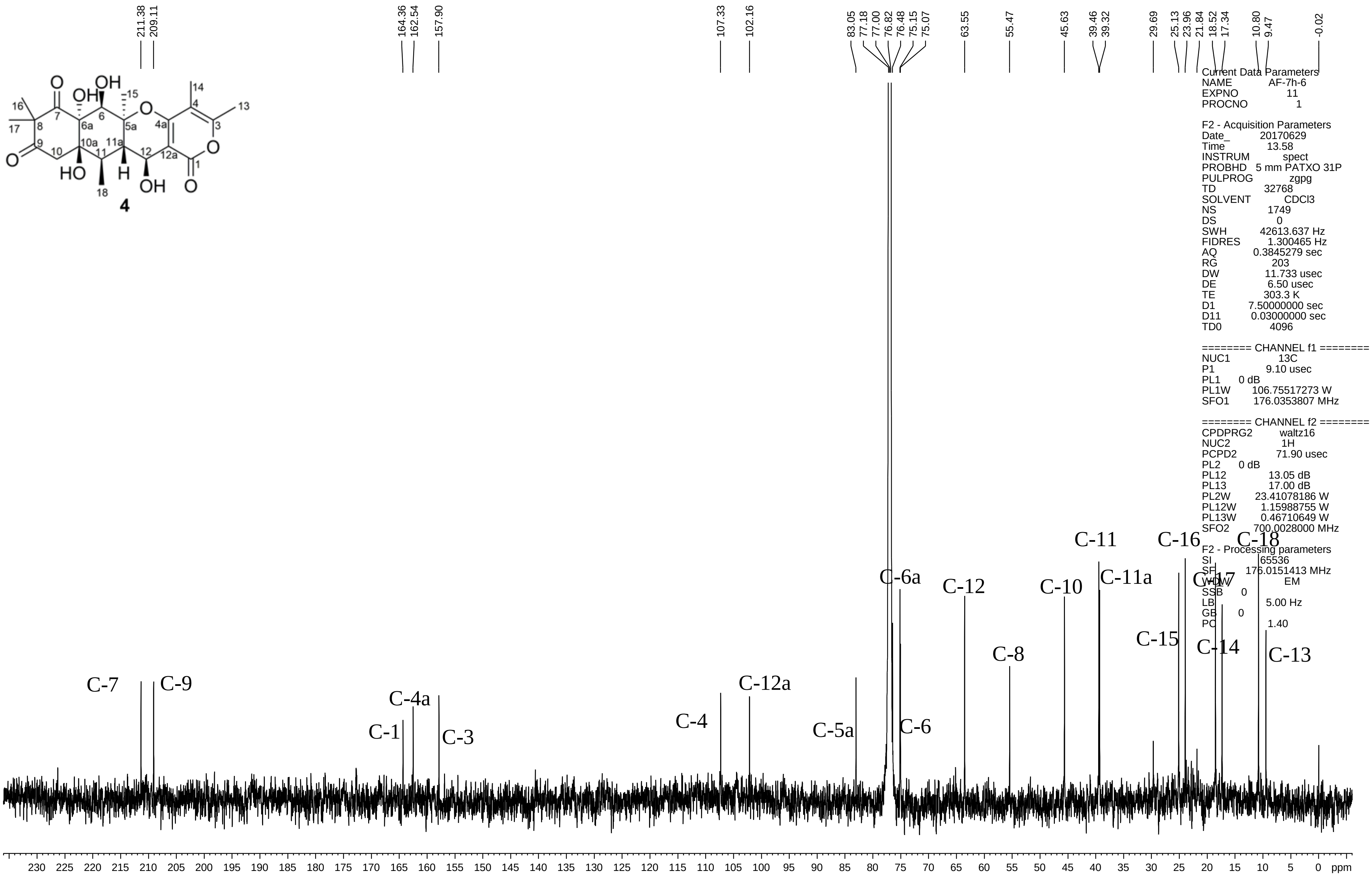

Figure S22.DEPT-135 (500 MHz, $CDCl_3$) spectrum of 12-*epi*-aspertetranone D (**4**)

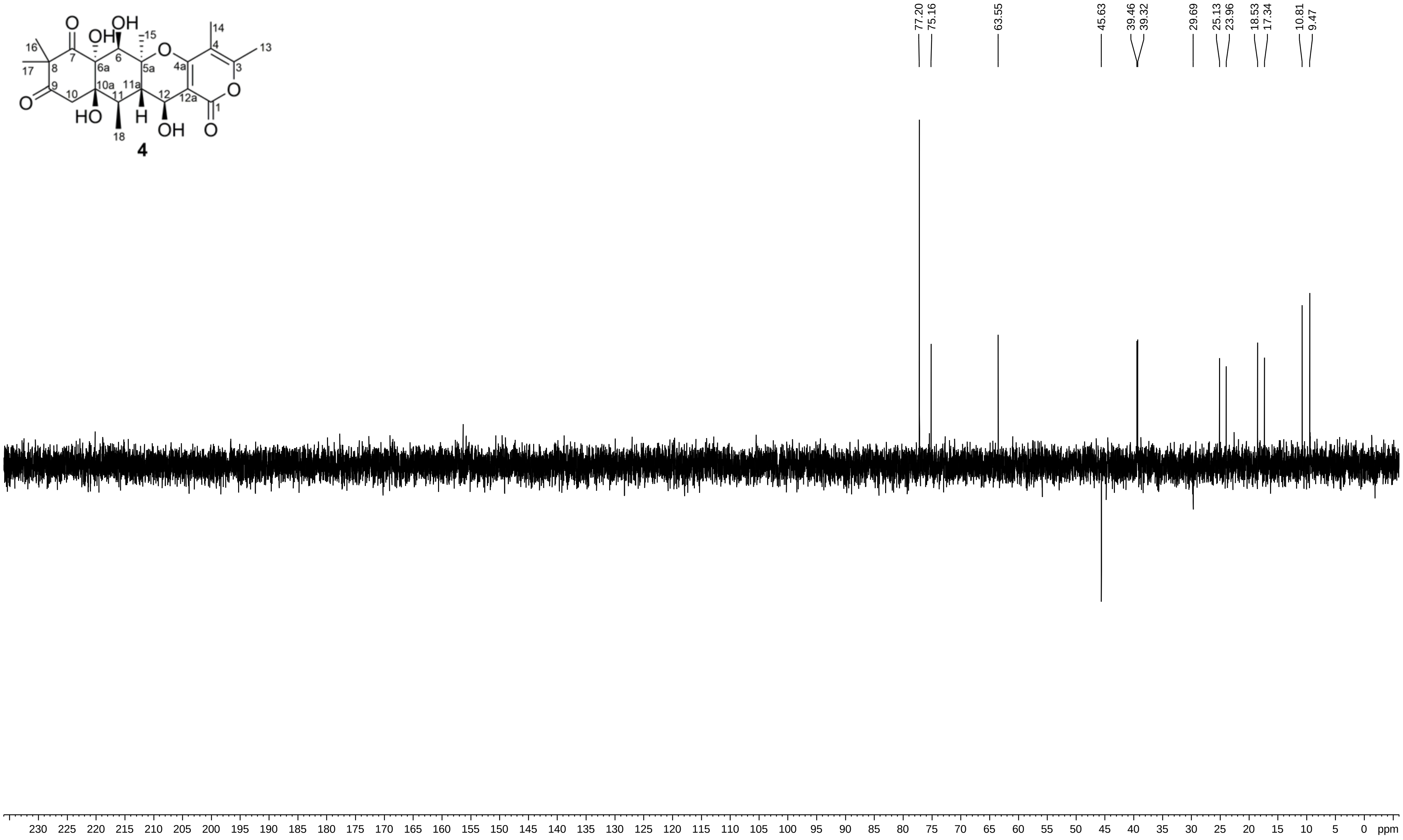

Figure S23. ^{1}H-^{1}H COSY (500 MHz, $CDCl_3$) spectrum of 12-*epi*-aspertetranone D (**4**)

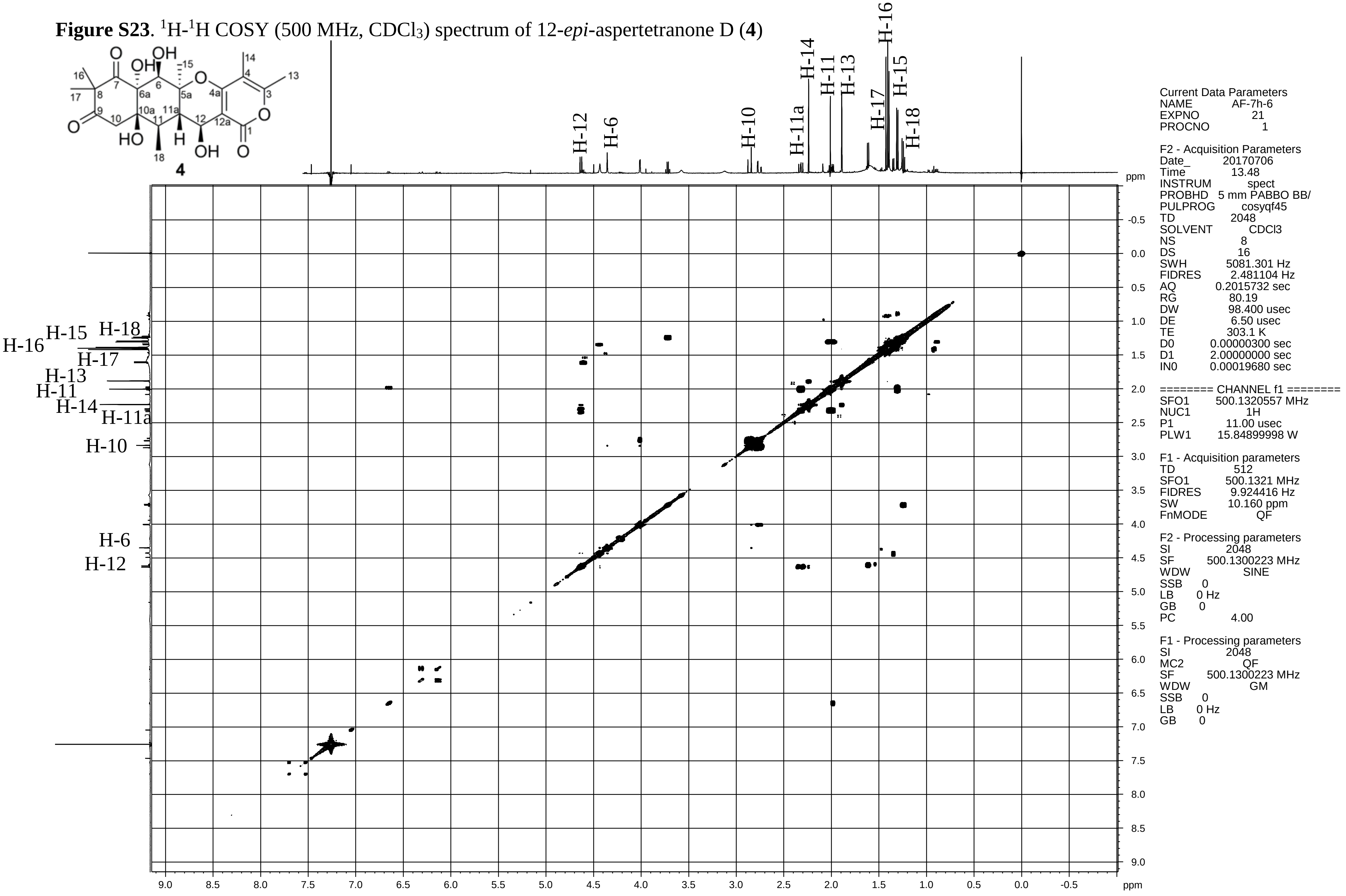

Figure S24. HSQC (500 MHz, $CDCl_3$) spectrum of 12-*epi*-aspertetranone D (**4**)

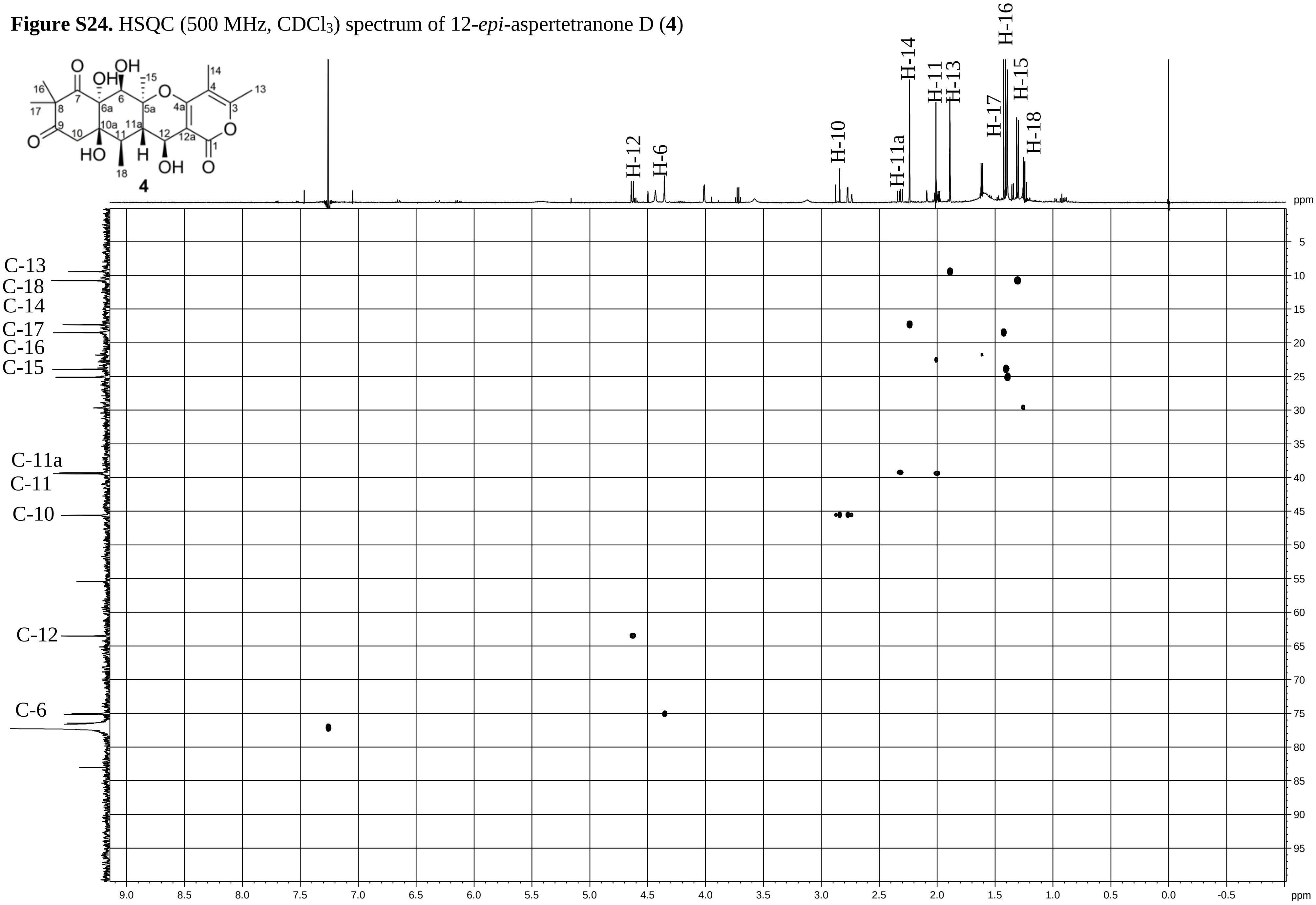

Figure S25. HMBC (500 MHz, $CDCl_3$) spectrum of 12-*epi*-aspertetranone D (**4**)

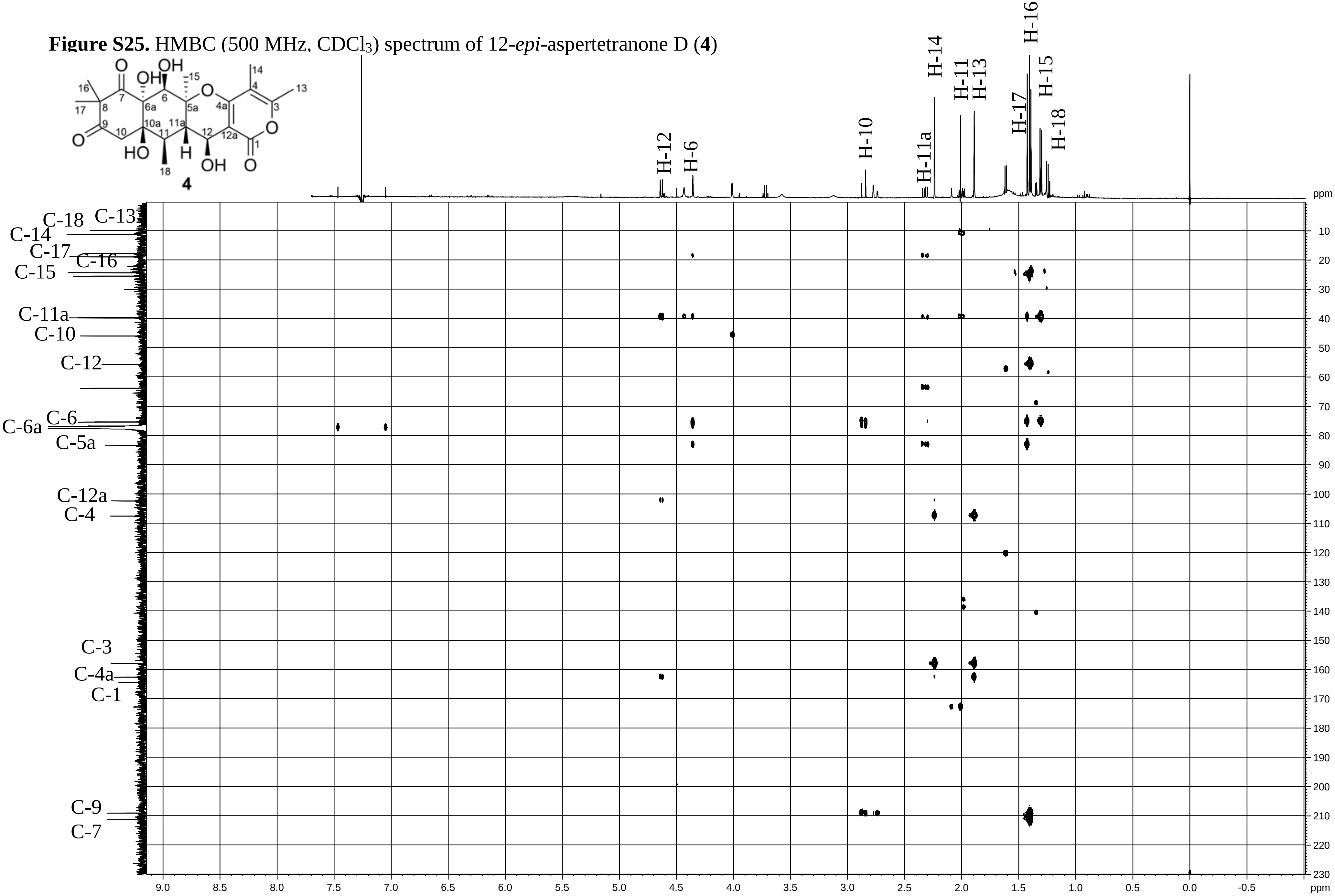

Figure S26. ROESY (500 MHz, $CDCl_3$) spectrum of 12-*epi*-aspertetranone D (**4**)

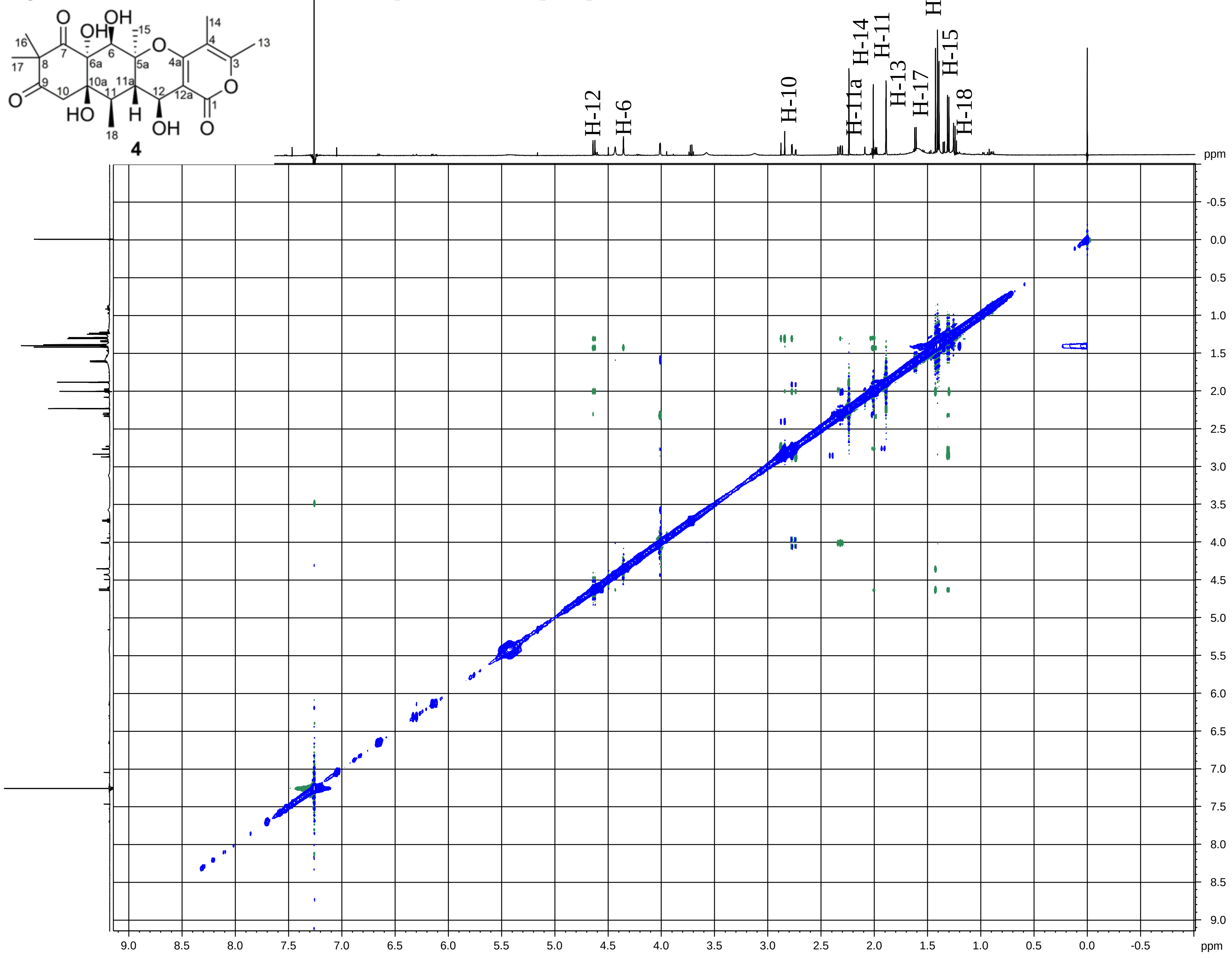

Current Data Parameters
NAME AF-7h-6
EXPNO 23
PROCNO 1

F2 - Acquisition Parameters
Date_ 20170706
Time 18.49
INSTRUM spect
PROBHD 5 mm PABBO BB/
PULPROG roesyph.2
TD 2048
SOLVENT CDCl3
NS 24
DS 16
SWH 5081.301 Hz
FIDRES 2.481104 Hz
AQ 0.2015732 sec
RG 4.19
DW 98.400 usec
DE 6.50 usec
TE 303.2 K
D0 0.00008740 sec
D1 2.00000000 sec
D12 0.00002000 sec
IN0 0.00019680 sec
L4 1350
P15 270000.00 usec

======== CHANNEL f1 ========
SFO1 500.1320557 MHz
NUC1 1H
P1 11.00 usec
P25 100.00 usec
PLW1 15.84899998 W
PLW27 0.76709002 W

F1 - Acquisition parameters
TD 256
SFO1 500.1321 MHz
FIDRES 19.848831 Hz
SW 10.160 ppm
FnMODE TPPI

F2 - Processing parameters
SI 2048
SF 500.1300224 MHz
WDW SINE
SSB 2
LB 0 Hz
GB 0
PC 4.00

F1 - Processing parameters
SI 512
MC2 TPPI
SF 500.1300185 MHz
WDW GM
SSB 2
LB 0 Hz
GB 0

Figure S27. ECD spectrum of 12-*epi*-aspertetranone D (**4**) in methanol

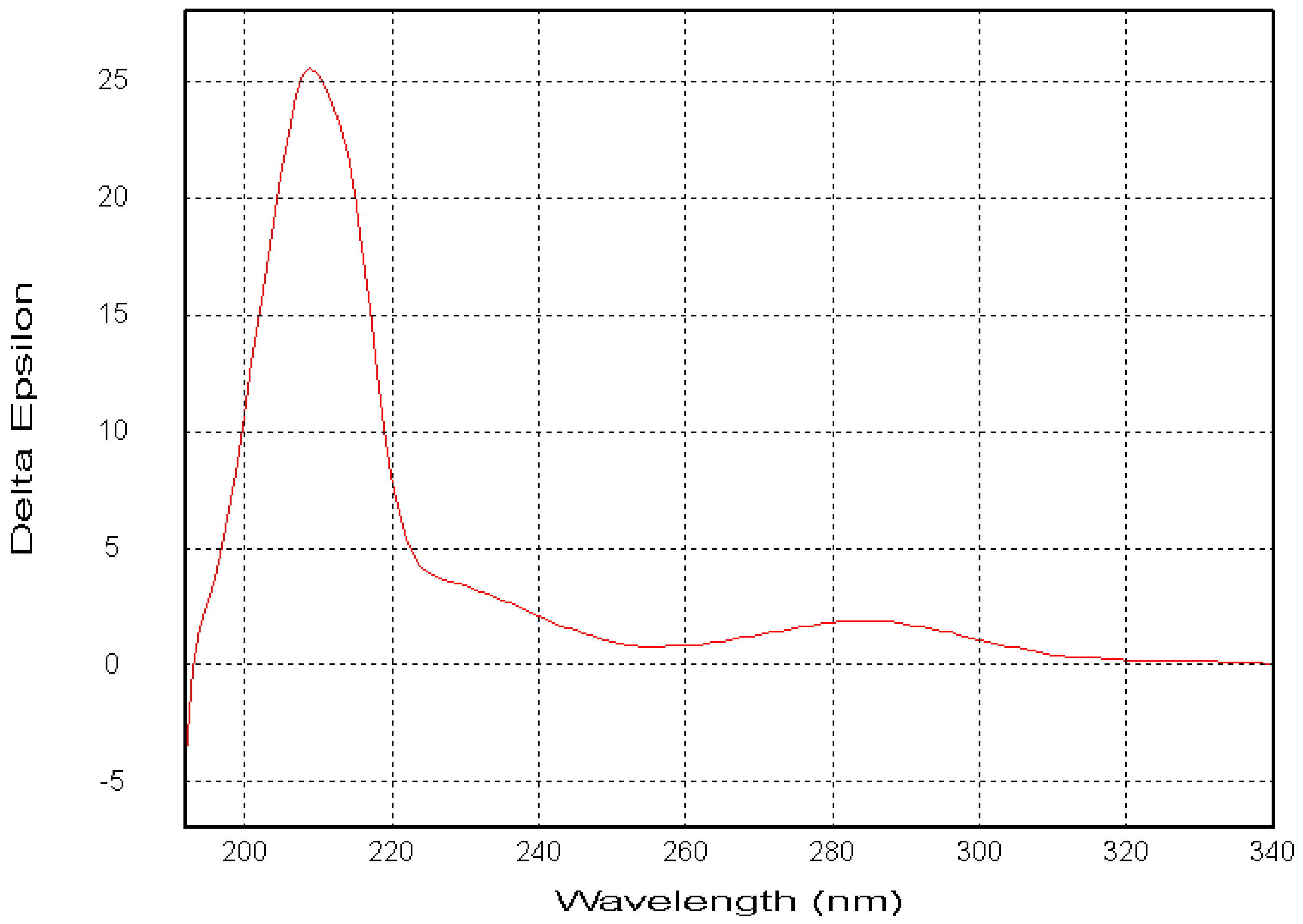

Figure S28. ^{1}H NMR (500 MHz, $CDCl_3$) spectrum of aspertetranone D (**5**)

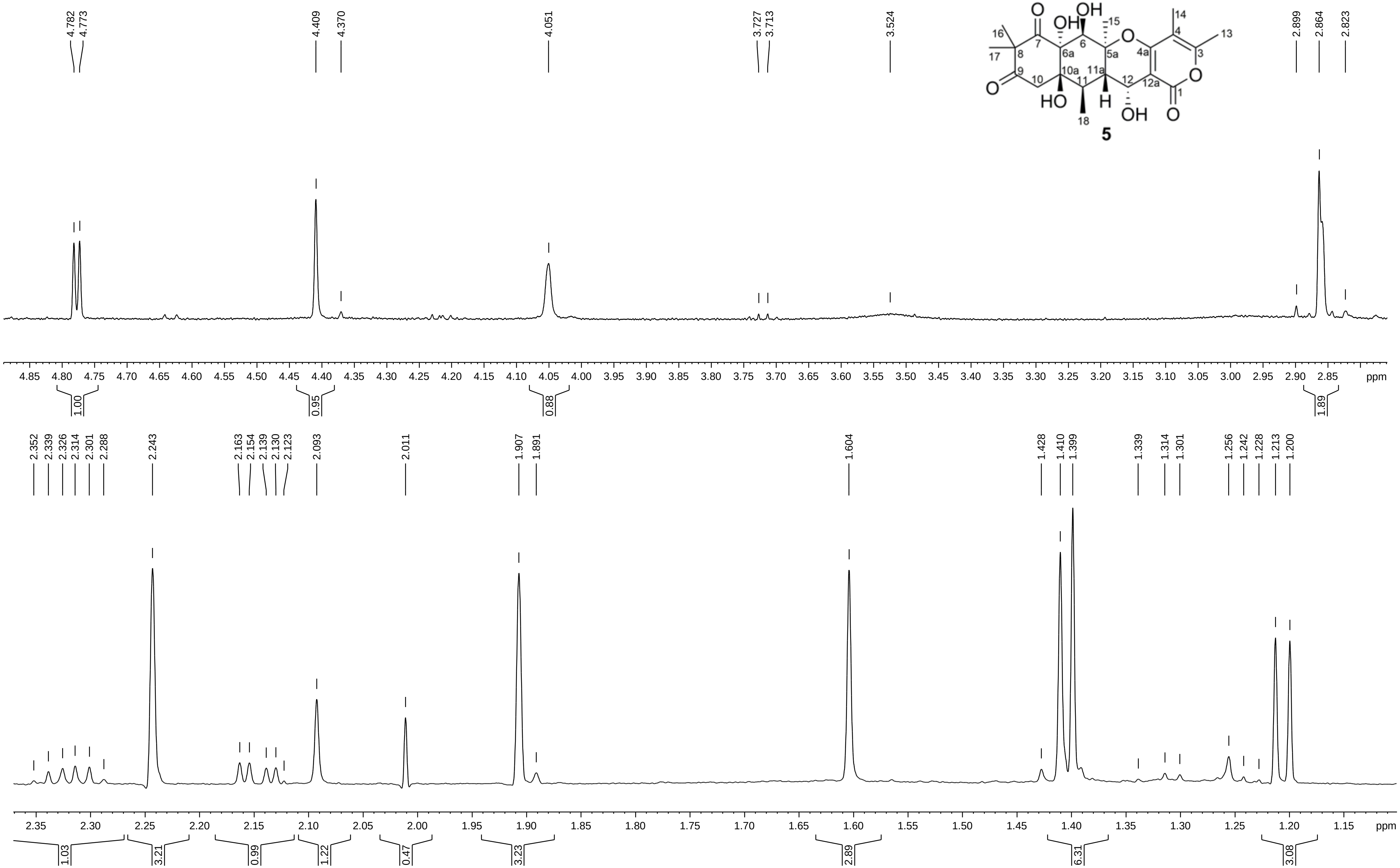

Figure S29. ^{13}C NMR (176 MHz, $CDCl_3$) spectrum of aspertetranone D (**5**)

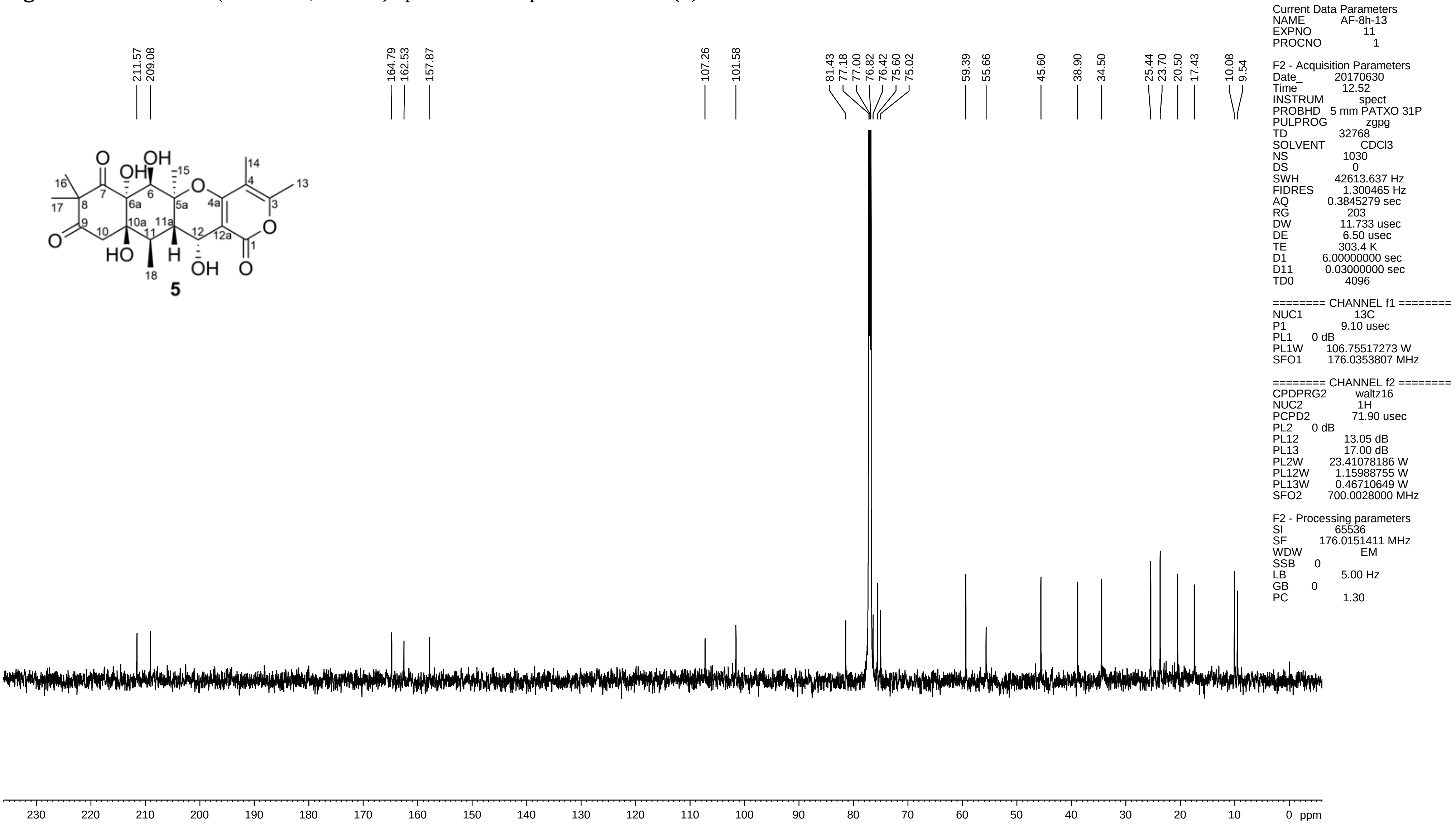

Figure S30. ^{1}H NMR (500 MHz, acetone-d_6) spectrum of aspertetranone A (**6**)

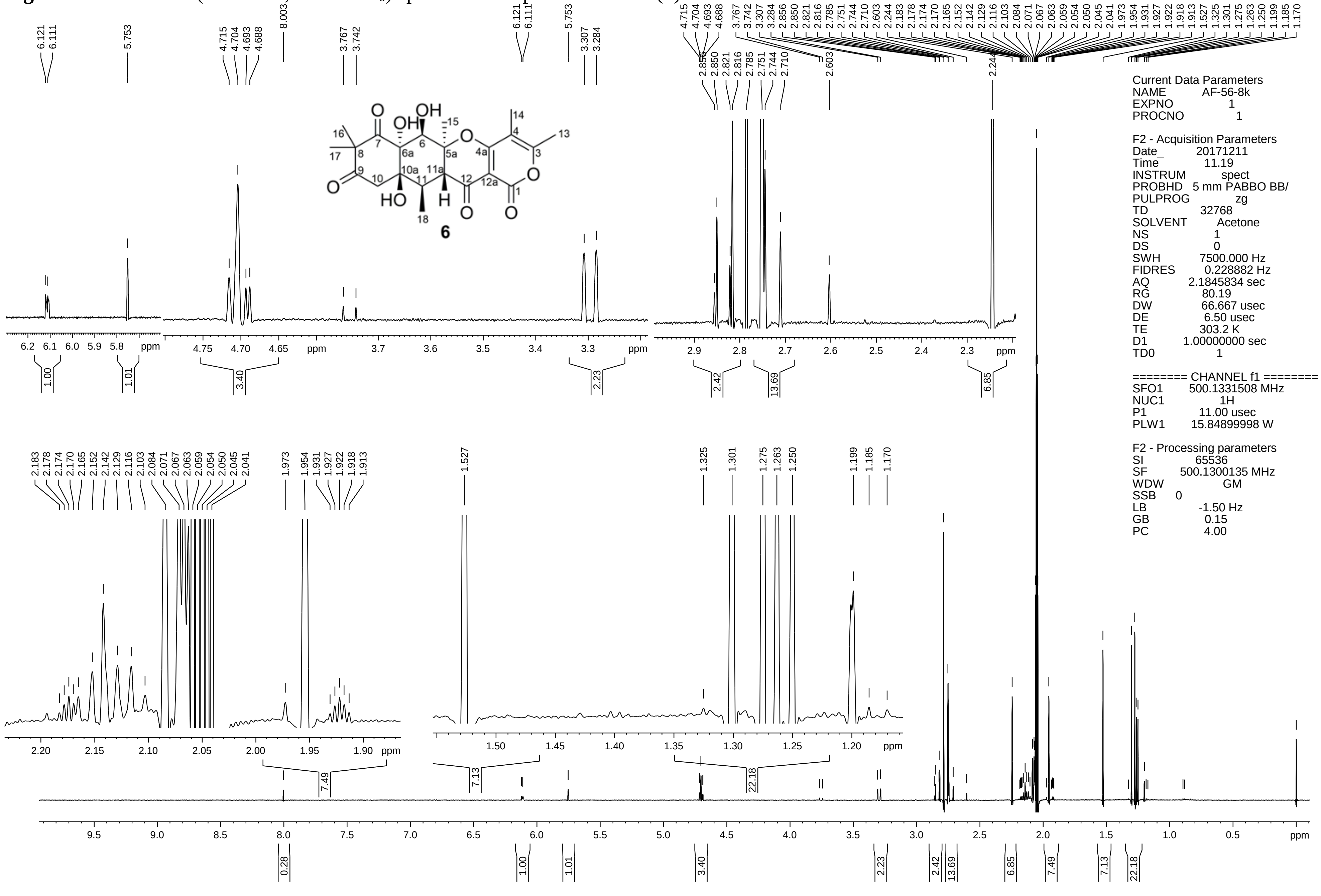

Figure S31. ^{13}C NMR (125 MHz, acetone-d$_6$) spectrum of aspertetranone A (**6**)

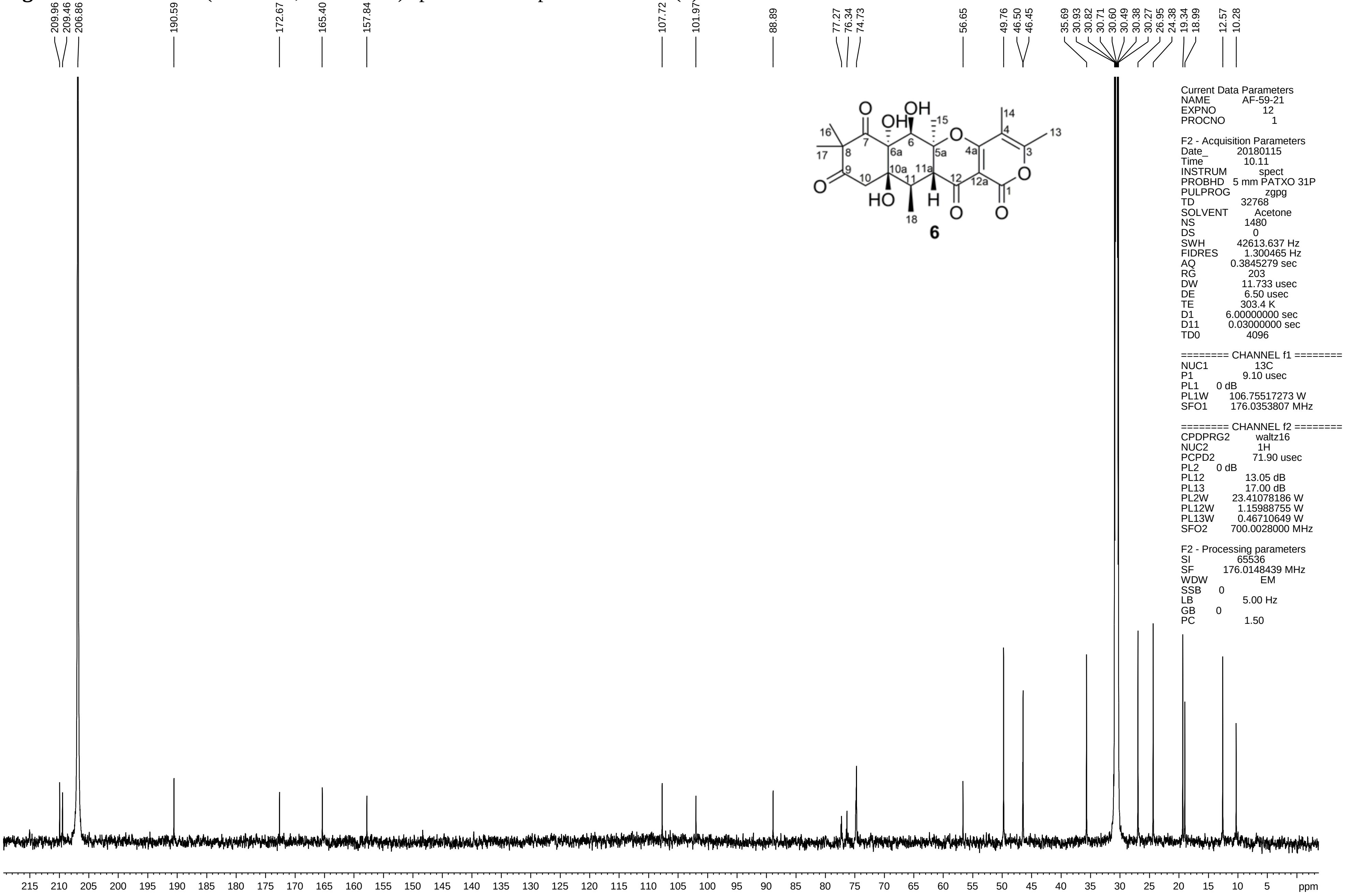

Figure S32. DEPT-135 (125 MHz, acetone-d_6) spectrum of aspertetranone A (**6**)

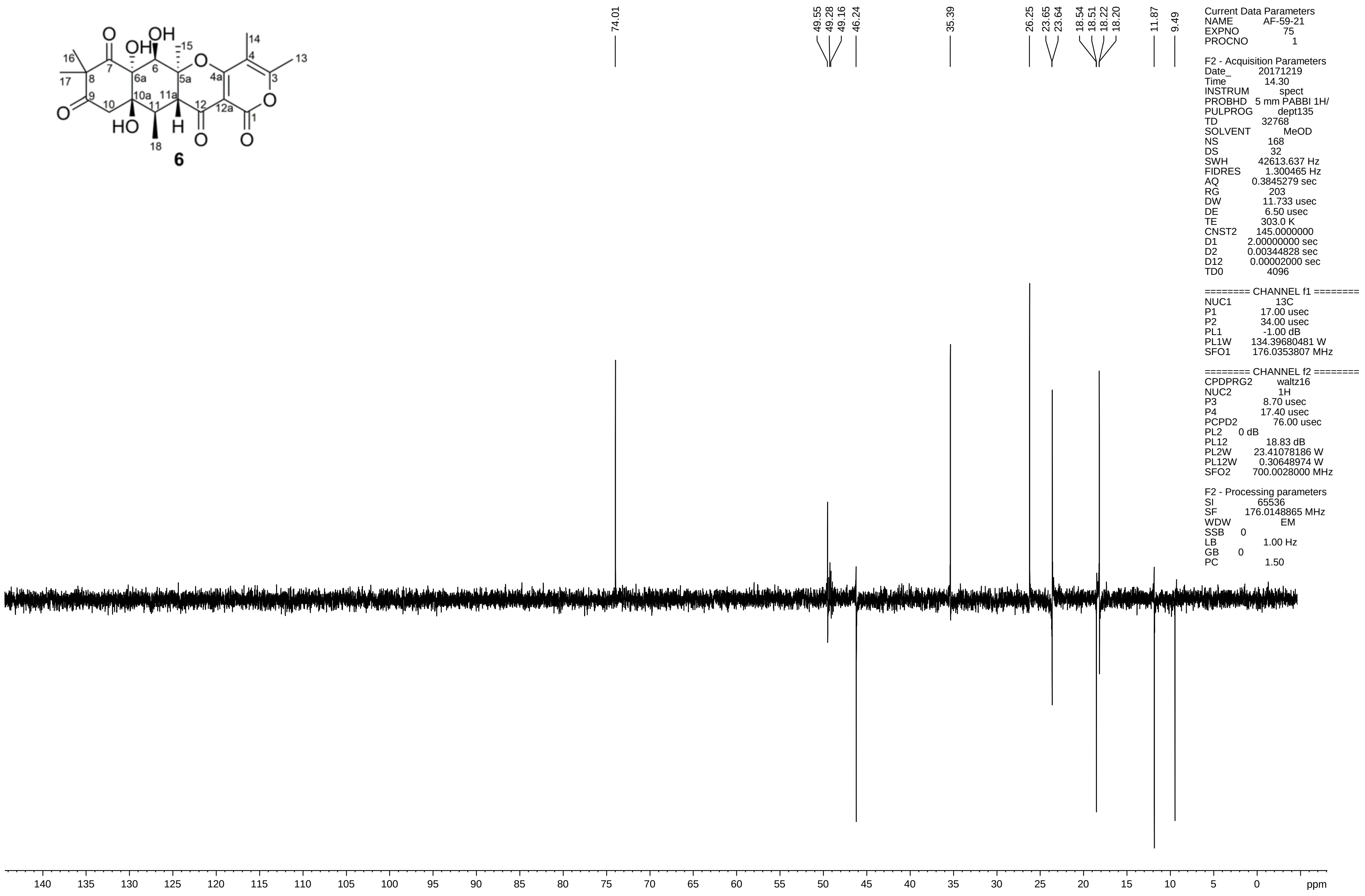

Figure S33. ^{1}H NMR (500 MHz, $CDCl_3$) spectrum of 6β,9α,14-trihydroxycinnamolide (**7**)

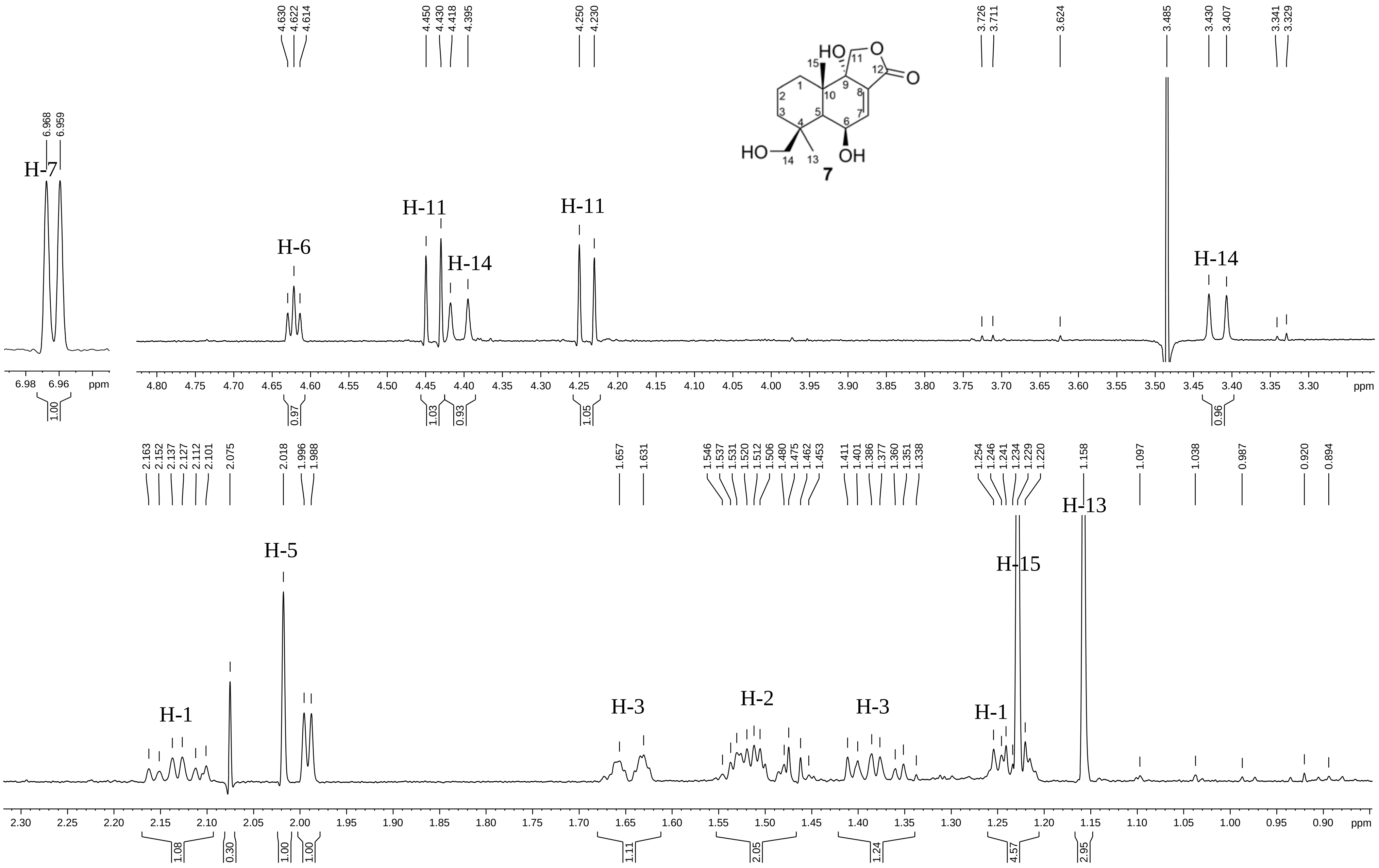

Figure S34. ^{13}C NMR (500 MHz, CDCl$_3$) spectrum of 6β,9α,14-trihydroxycinnamolide (**7**)

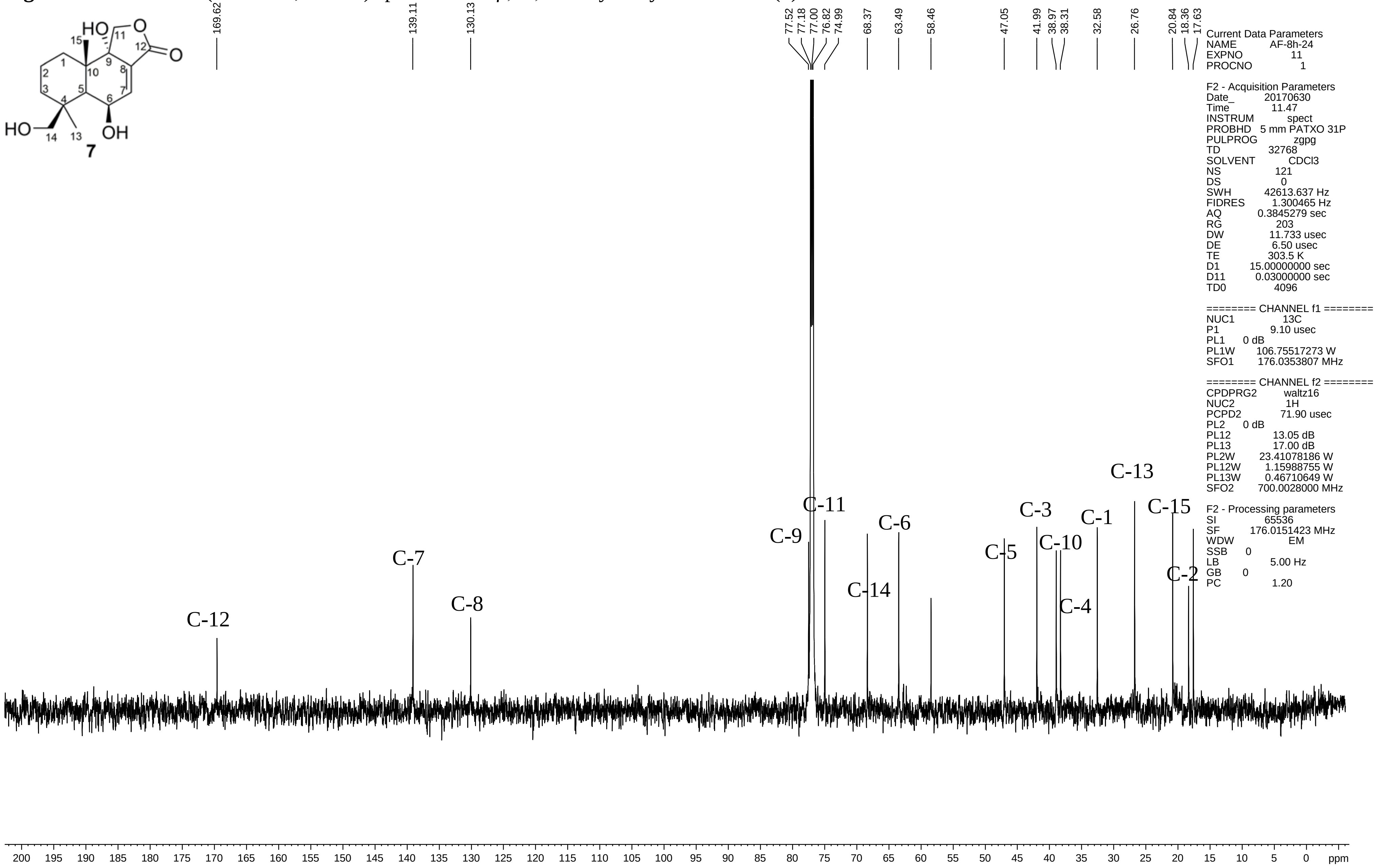

Figure S35. DEPT-135 (500 MHz, $CDCl_3$) spectrum of 6β,9α,14-trihydroxycinnamolide (**7**)

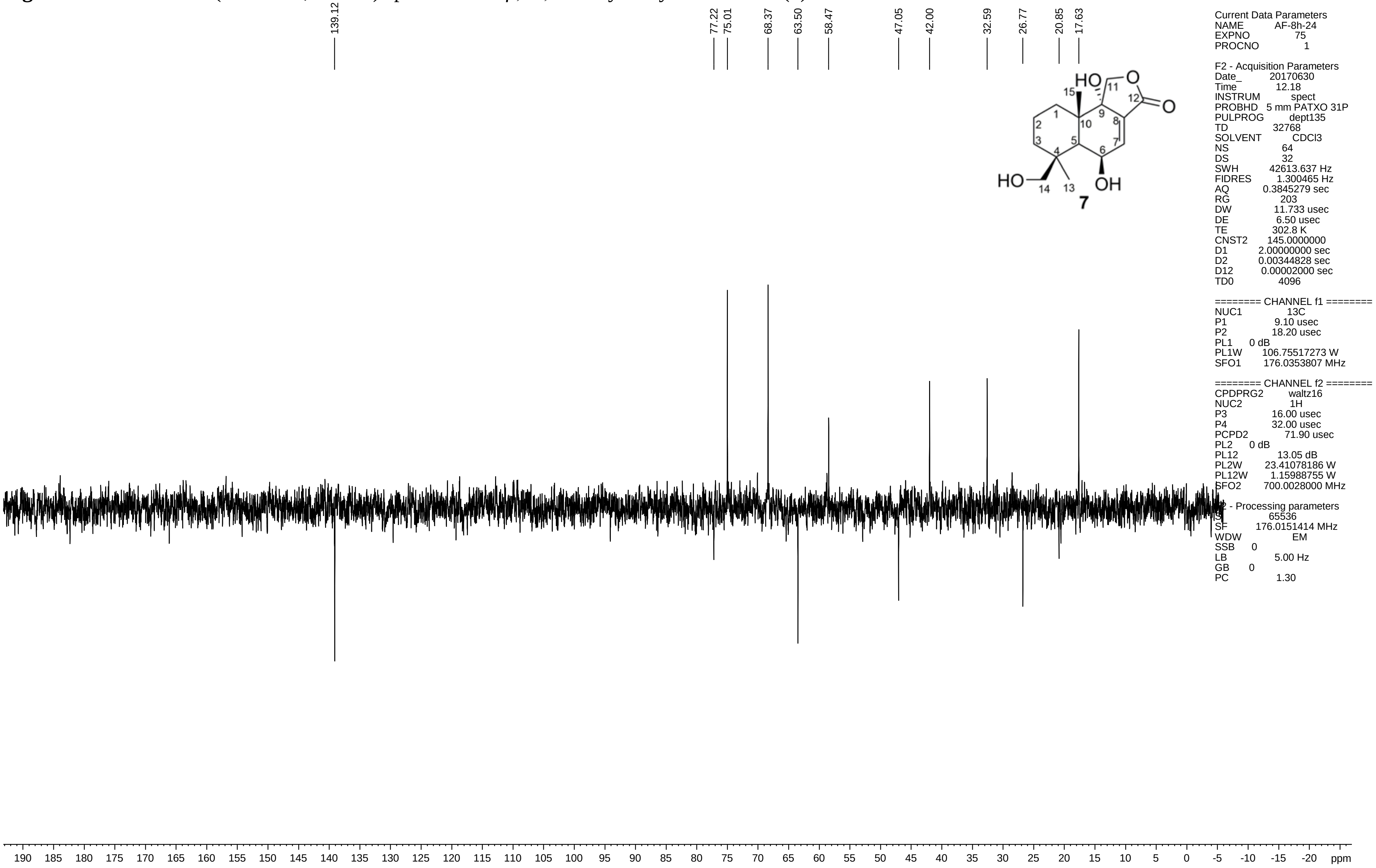

Figure S36. ^{1}H-^{1}H COSY (500 MHz, $CDCl_3$) spectrum of 6β,9α,14-trihydroxycinnamolide (**7**)

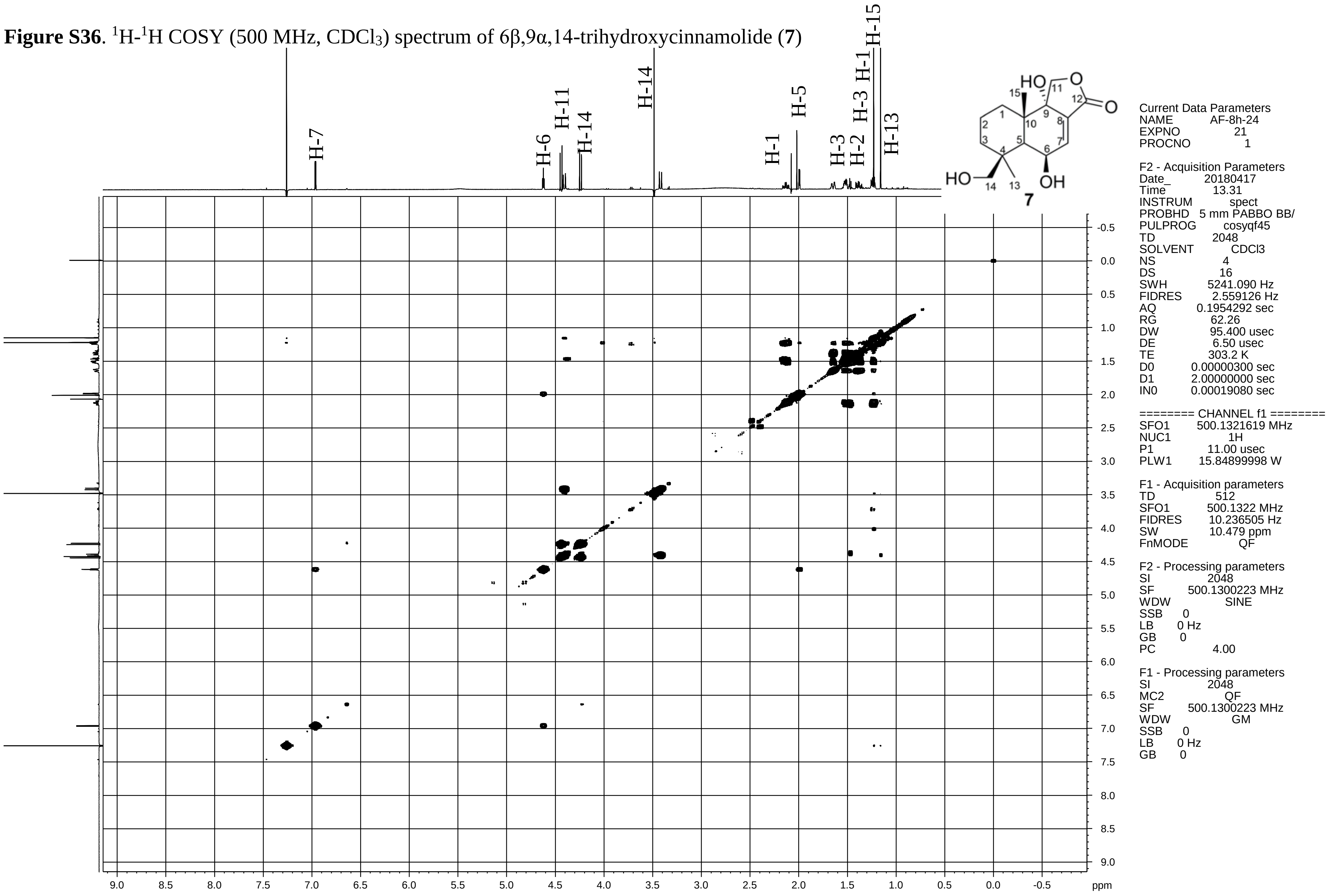

Figure S37. HSQC (500 MHz, $CDCl_3$) spectrum of 6β,9α,14-trihydroxycinnamolide (**7**)

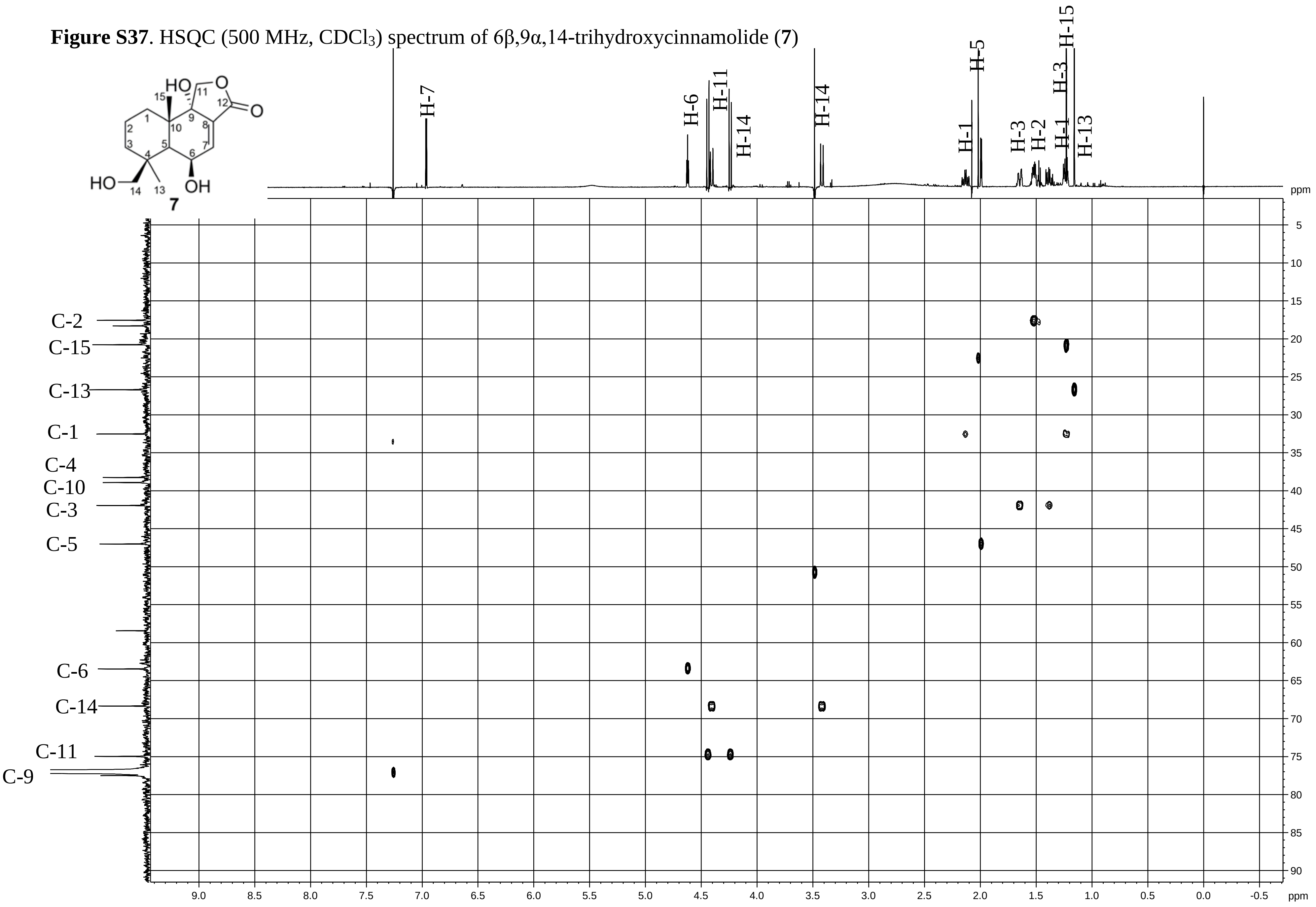

Figure S38. HMBC (500 MHz, $CDCl_3$) spectrum of 6β,9α,14-trihydroxycinnamolide (**7**)

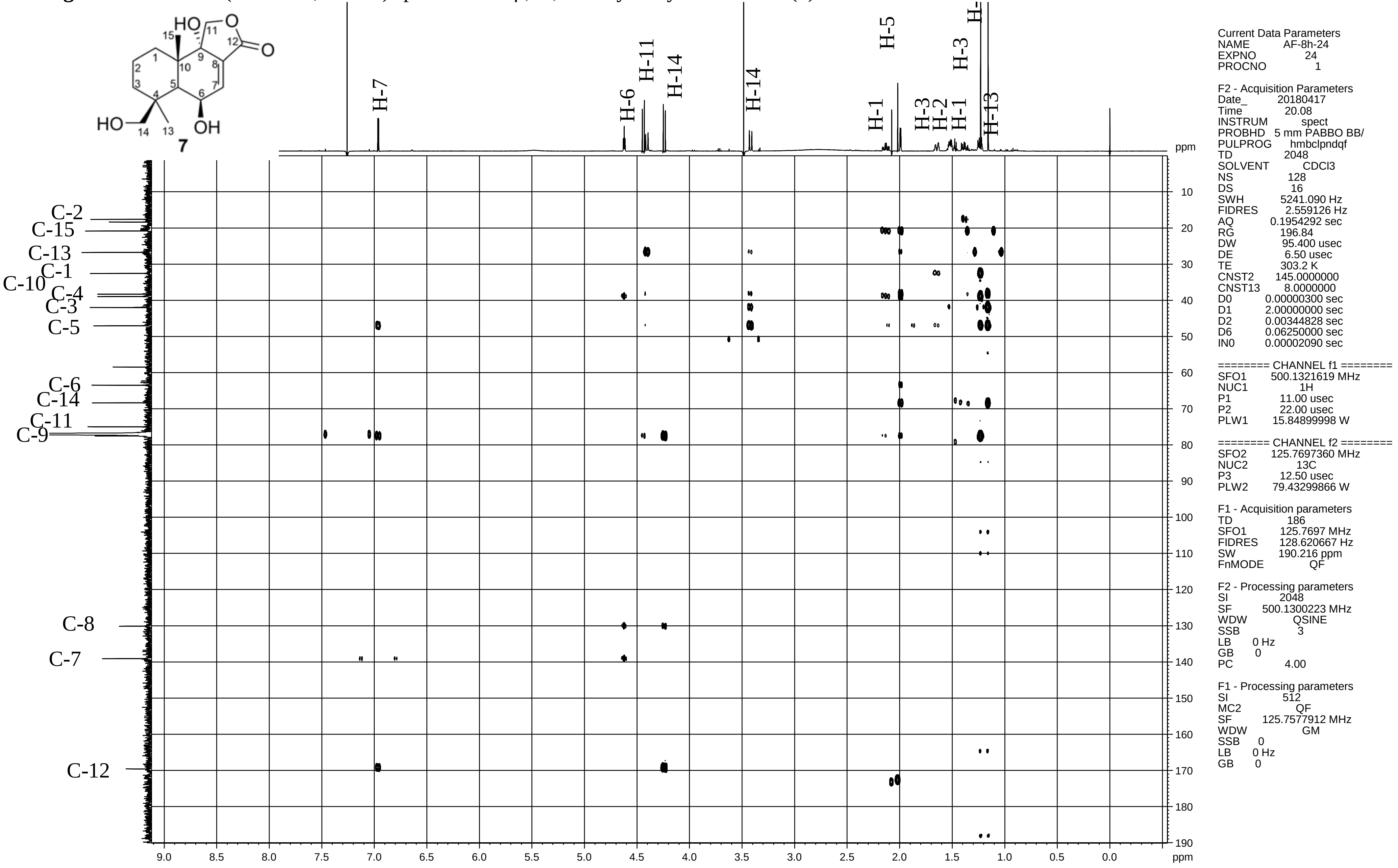

Figure S39. ROESY (500 MHz, $CDCl_3$) spectrum of 6β,9α,14-trihydroxycinnamolide (**7**)

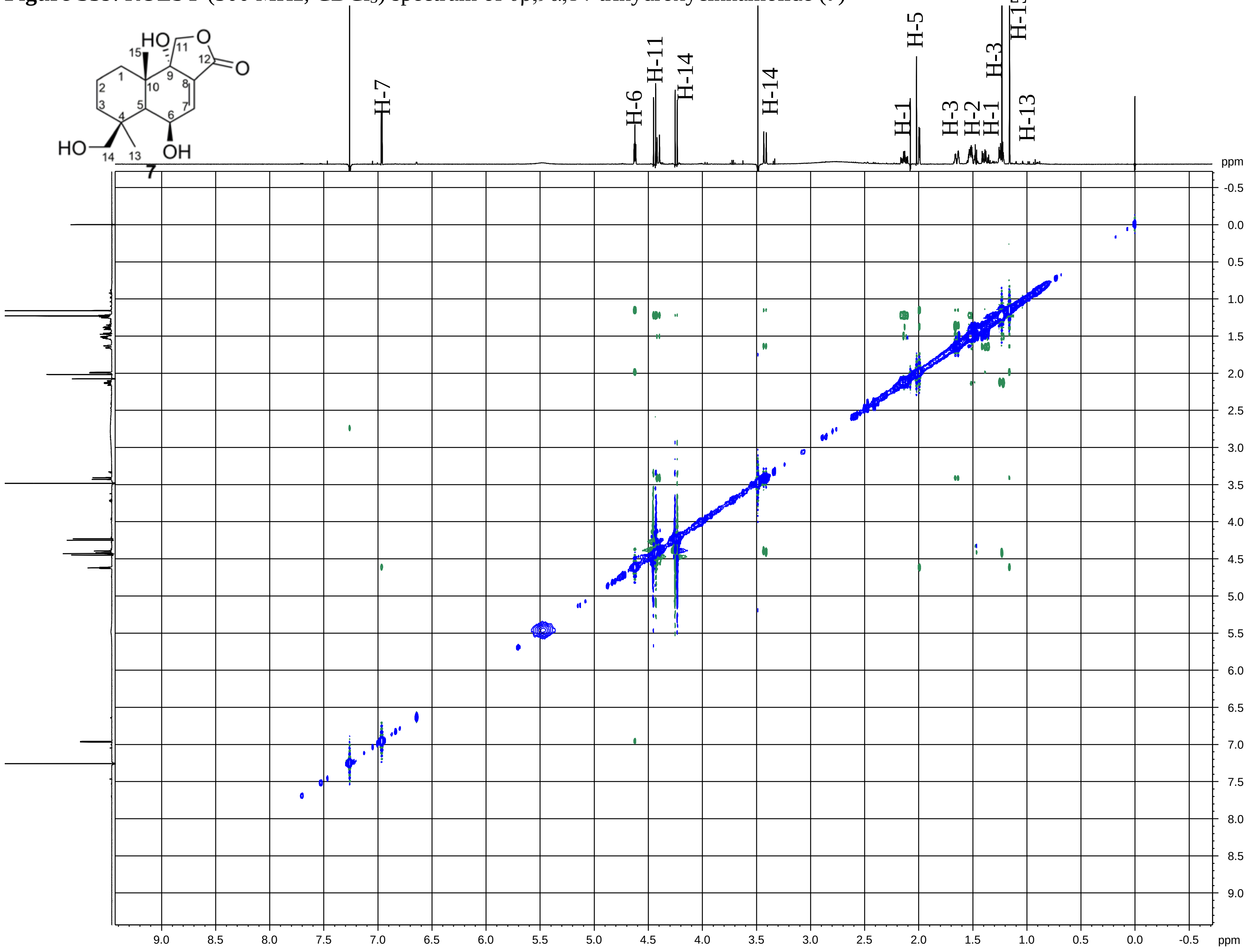

Current Data Parameters
NAME AF-8h-24
EXPNO 23
PROCNO 1

F2 - Acquisition Parameters
Date_ 20180417
Time 17.14
INSTRUM spect
PROBHD 5 mm PABBO BB/
PULPROG roesyph.2
TD 2048
SOLVENT CDCl3
NS 16
DS 16
SWH 5241.090 Hz
FIDRES 2.559126 Hz
AQ 0.1954292 sec
RG 1.04
DW 95.400 usec
DE 6.50 usec
TE 303.1 K
D0 0.00008440 sec
D1 2.00000000 sec
D12 0.00002000 sec
IN0 0.00019080 sec
L4 1350
P15 270000.00 usec

======== CHANNEL f1 ========
SFO1 500.1321619 MHz
NUC1 1H
P1 11.00 usec
P25 100.00 usec
PLW1 15.84899998 W
PLW27 0.76709002 W

F1 - Acquisition parameters
TD 256
SFO1 500.1322 MHz
FIDRES 20.473009 Hz
SW 10.479 ppm
FnMODE TPPI

F2 - Processing parameters
SI 2048
SF 500.1300223 MHz
WDW SINE
SSB 2
LB 0 Hz
GB 0
PC 4.00

F1 - Processing parameters
SI 512
MC2 TPPI
SF 500.1300223 MHz
WDW GM
SSB 2
LB 0 Hz
GB 0

Figure S40. ECD spectrum of 6β,9α,14-trihydroxycinnamolide (**7**) in methanol

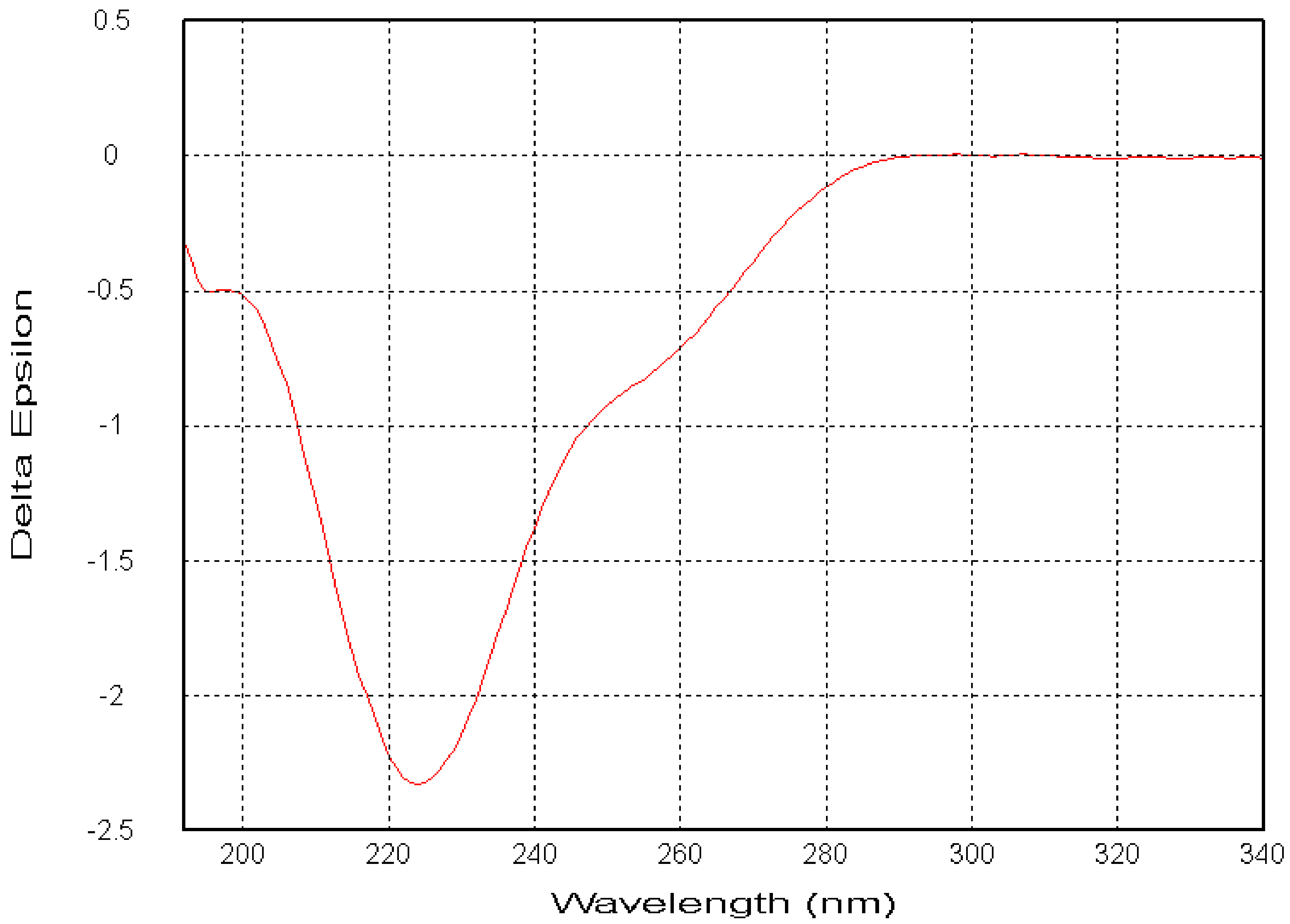

Figure S41. ^{1}H NMR (500 MHz, acetone-d_6) spectrum of insulicolide A (**8**)

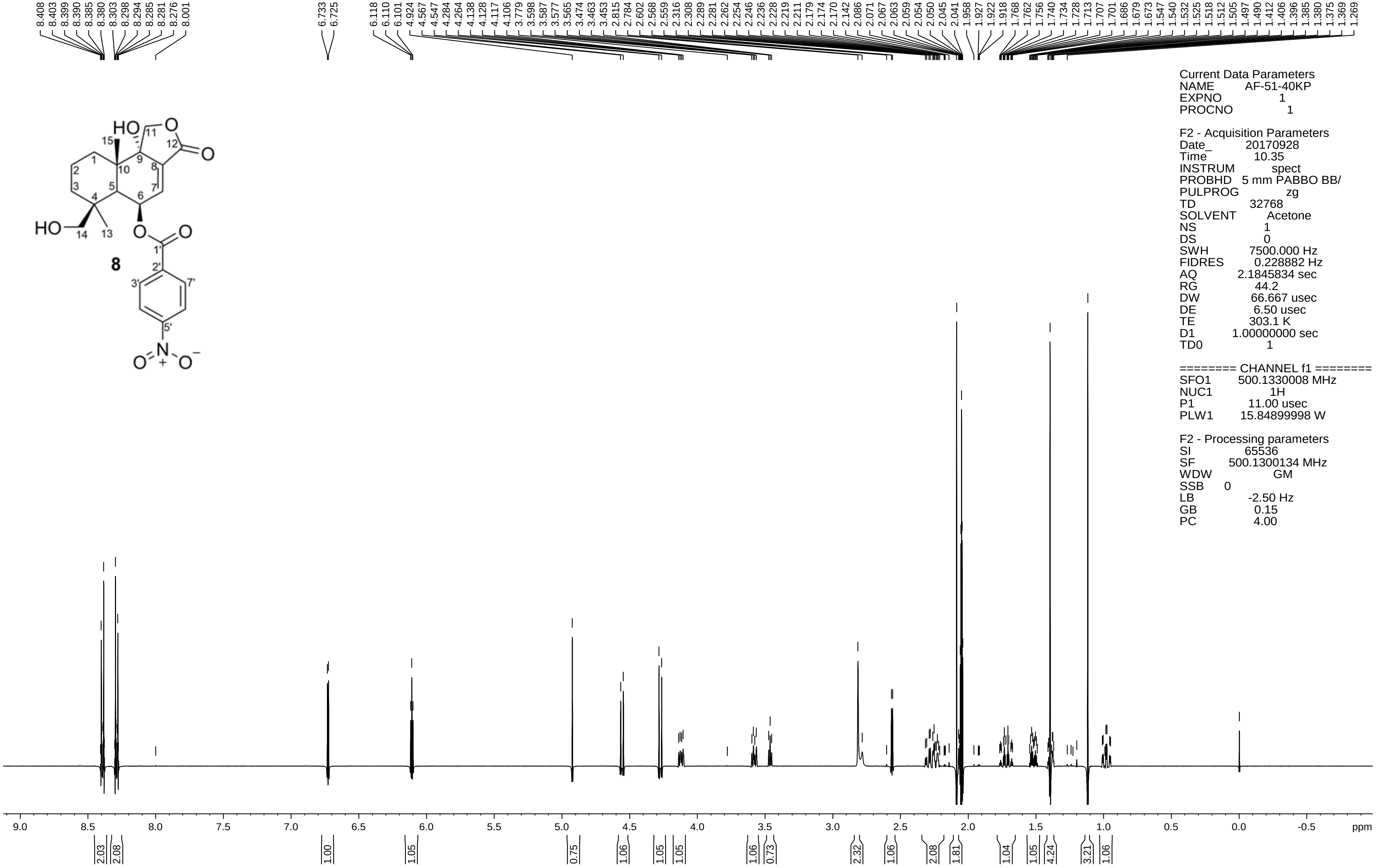

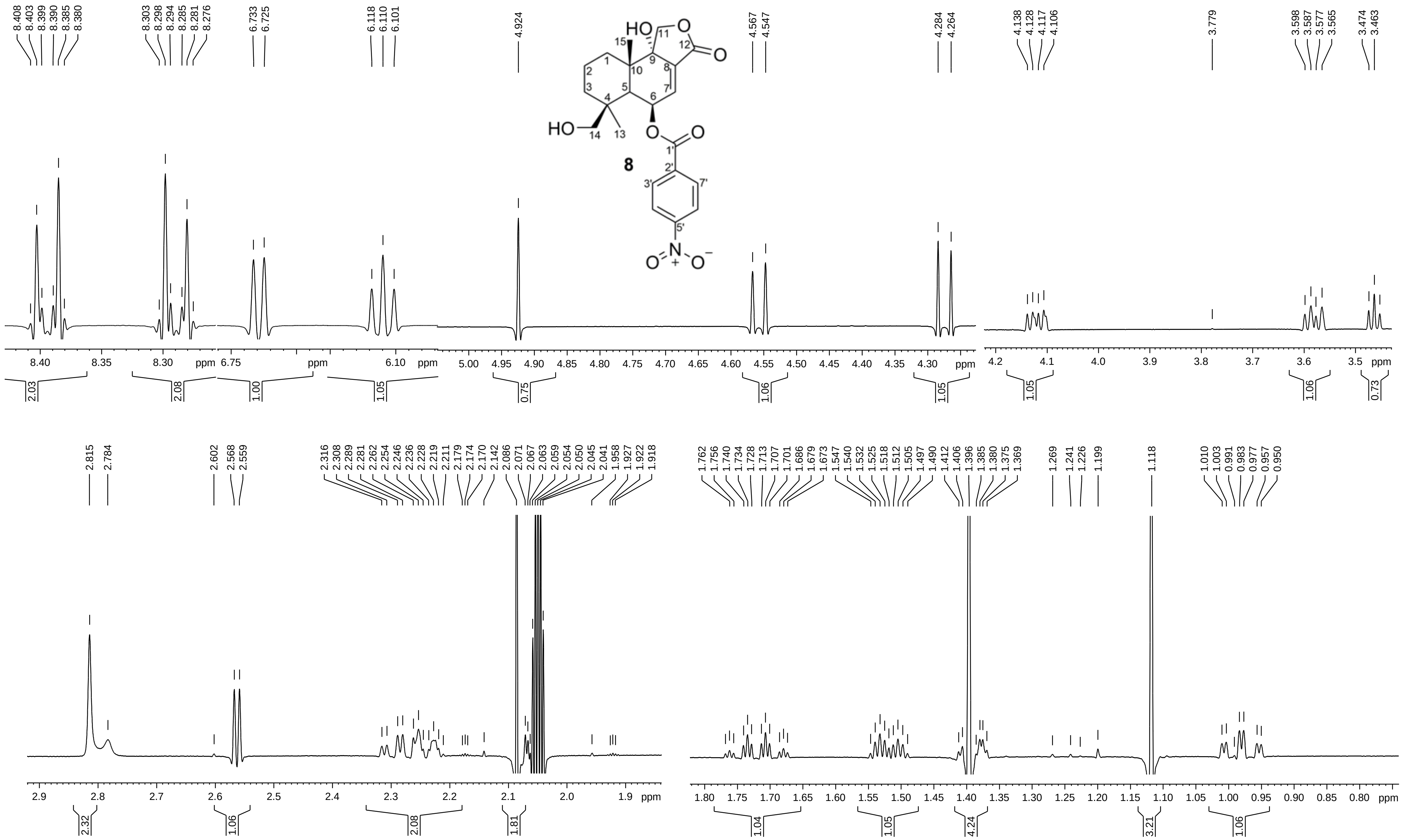
8.408 8.403 8.399 8.390 8.385 8.380
8.303 8.298 8.294 8.285 8.281 8.276
6.733 6.725
6.118 6.110 6.101
4.924
4.567 4.547
4.284 4.264
4.138 4.128 4.117 4.106
3.779
3.598 3.587 3.577 3.565
3.474 3.463
ppm
2.03 2.08 1.00 1.05 0.75 1.06 1.05 1.05 1.06 0.73
8
2.815 2.784
2.602 2.568 2.559
2.316 2.308 2.289 2.281 2.262 2.254 2.246 2.236 2.228 2.219 2.211 2.179 2.174 2.170 2.142 2.086 2.071 2.067 2.063 2.059 2.054 2.050 2.045 2.041 1.958 1.927 1.922 1.918
1.762 1.756 1.740 1.734 1.728 1.713 1.707 1.701 1.686 1.679 1.673 1.547 1.540 1.532 1.525 1.518 1.512 1.505 1.497 1.490 1.412 1.406 1.396 1.385 1.380 1.375 1.369
1.269 1.241 1.226 1.199
1.118
1.010 1.003 0.991 0.983 0.977 0.957 0.950
2.32 1.06 2.08 1.81 1.04 1.05 4.24 3.21 1.06

Figure S42. ^{13}C NMR (125 MHz, acetone-d_6) spectrum of insulicolide A (**8**)

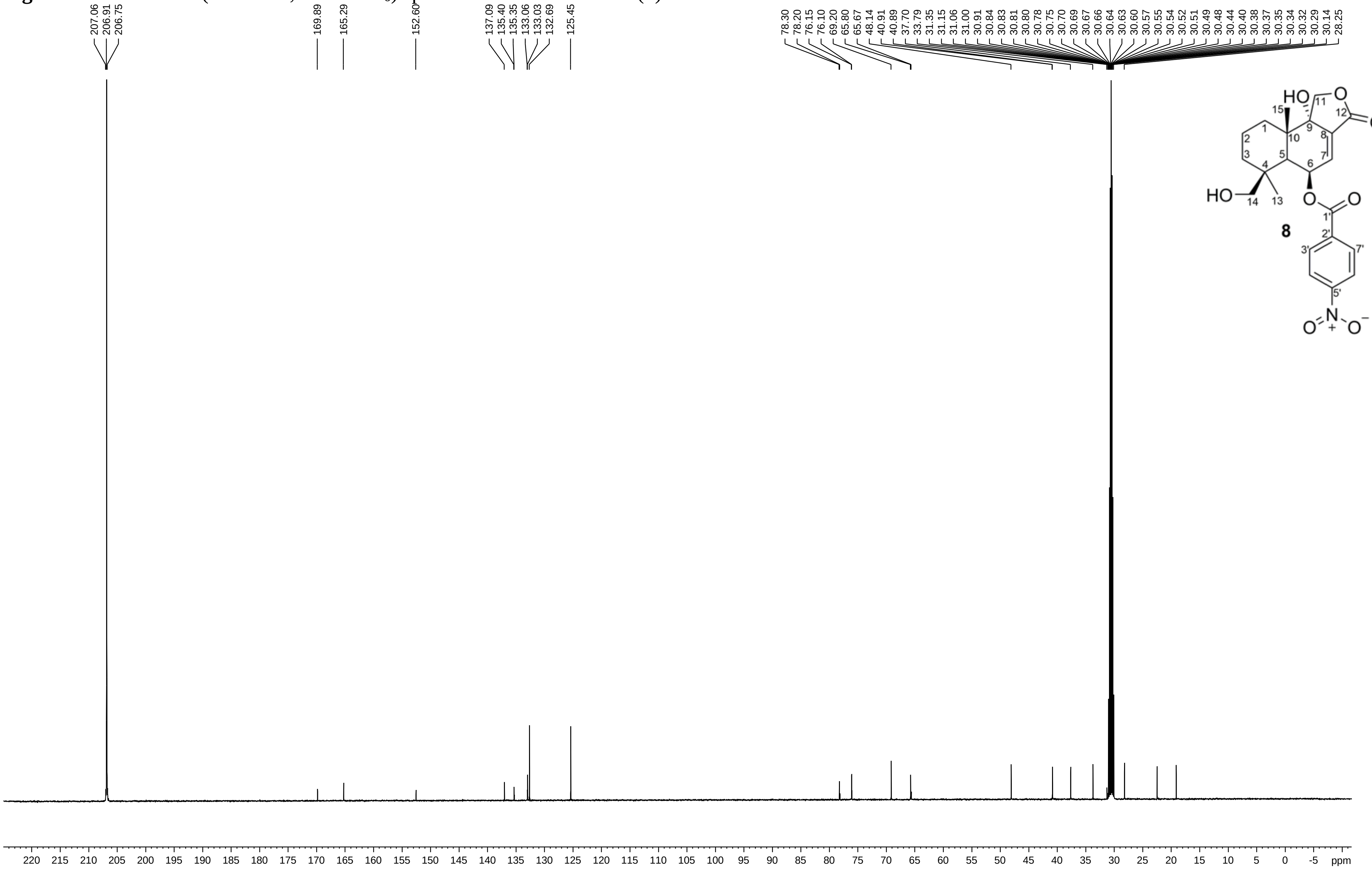

Figure S43. DEPT-135 (125 MHz, acetone-d_6) spectrum of insulicolide A (**8**)

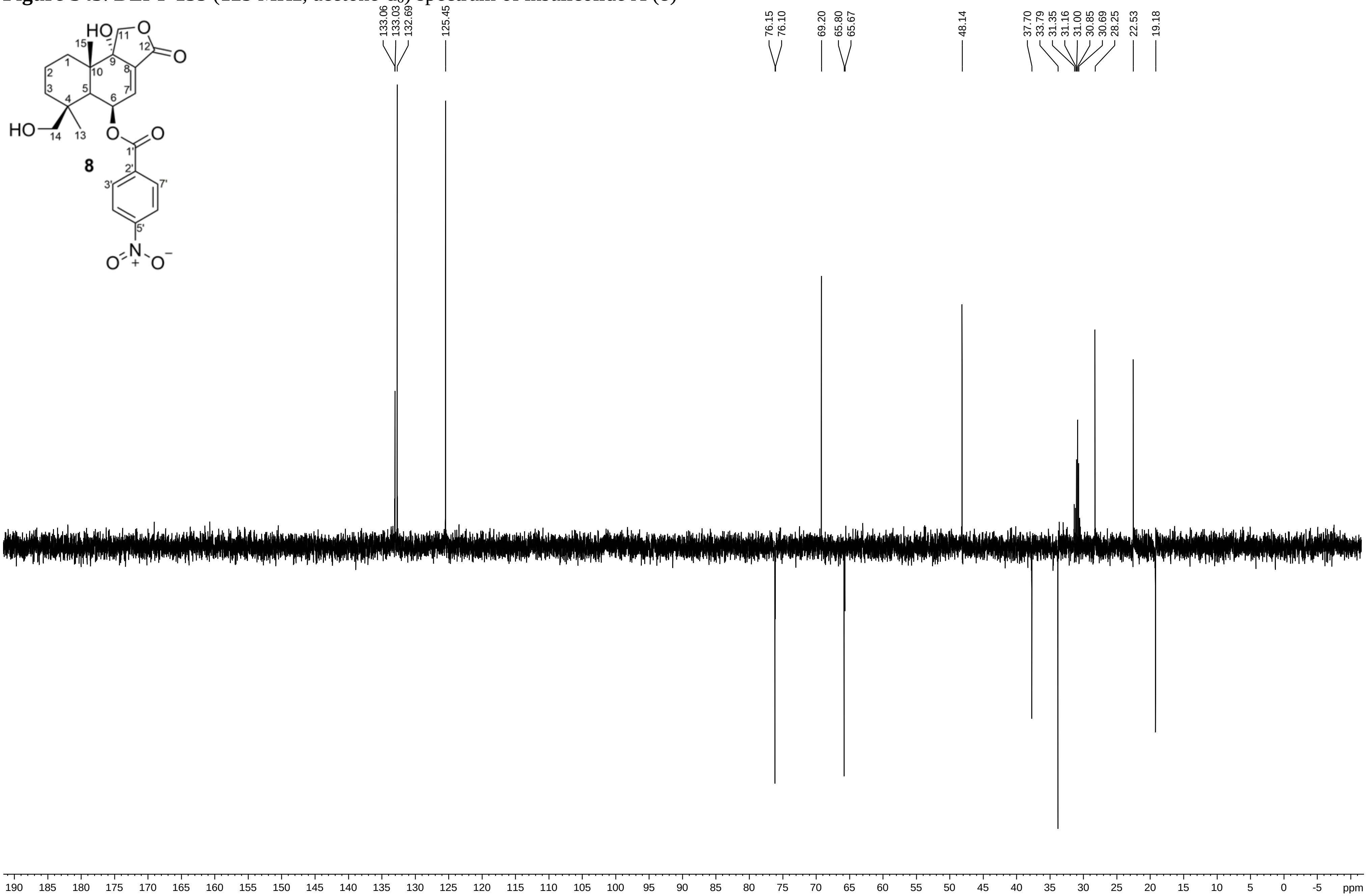

Figure S44. ECD spectrum of insulicolide A (**8**) in methanol

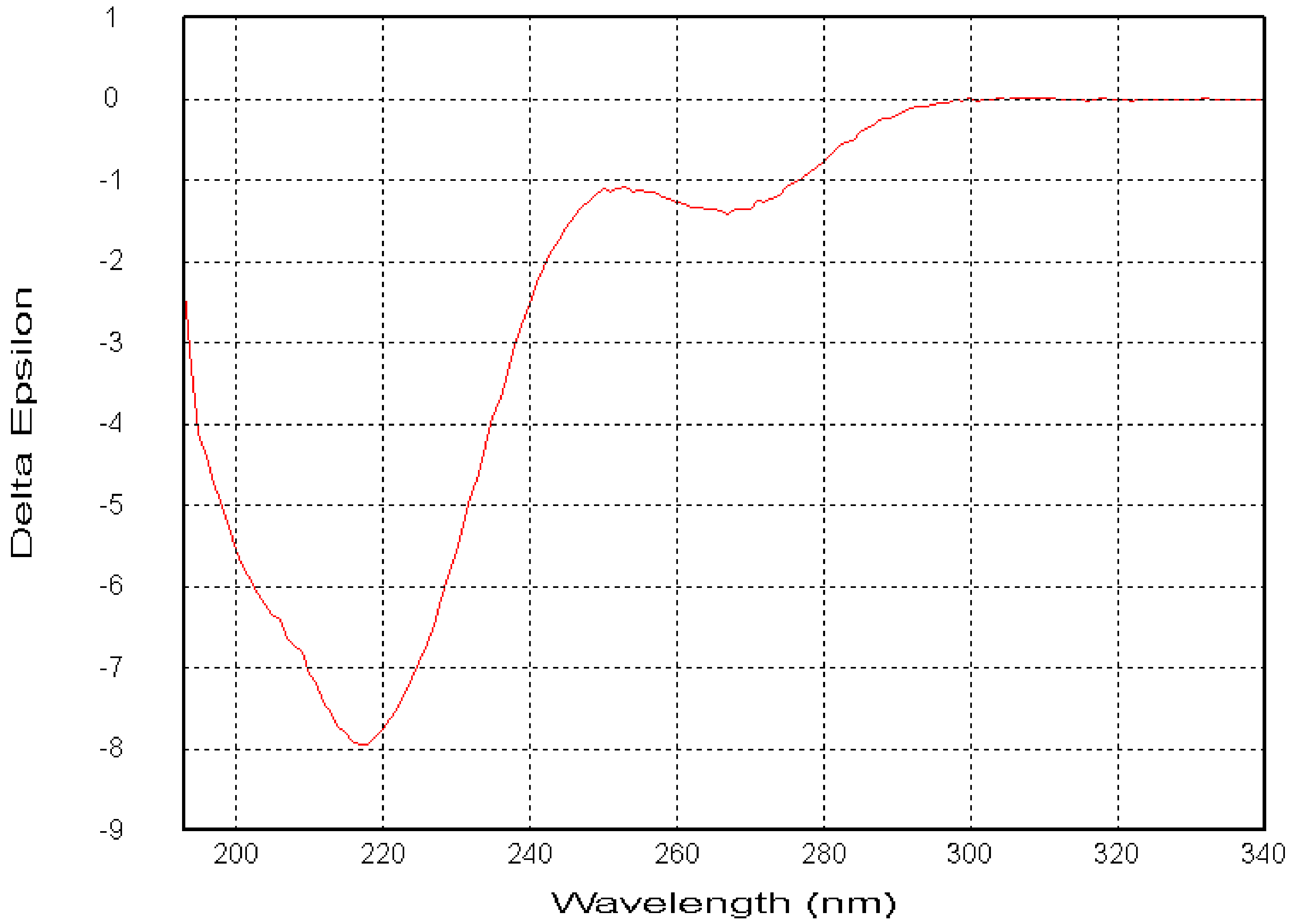

Figure S45. ^{1}H NMR (700 MHz, DMSO-d$_6$) spectrum of 6β,7α,14-trihydroxyconfertifolin (**9**)

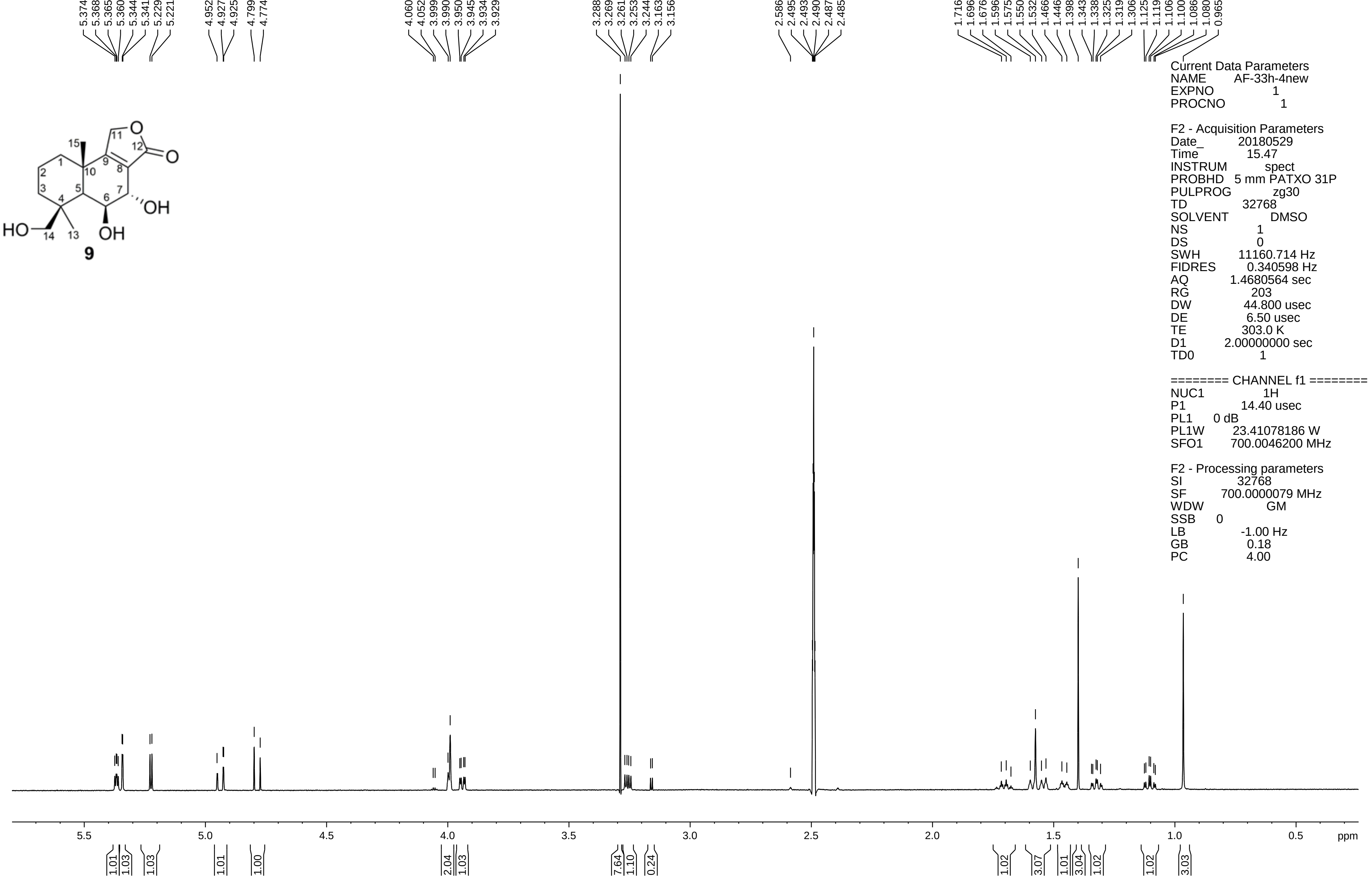

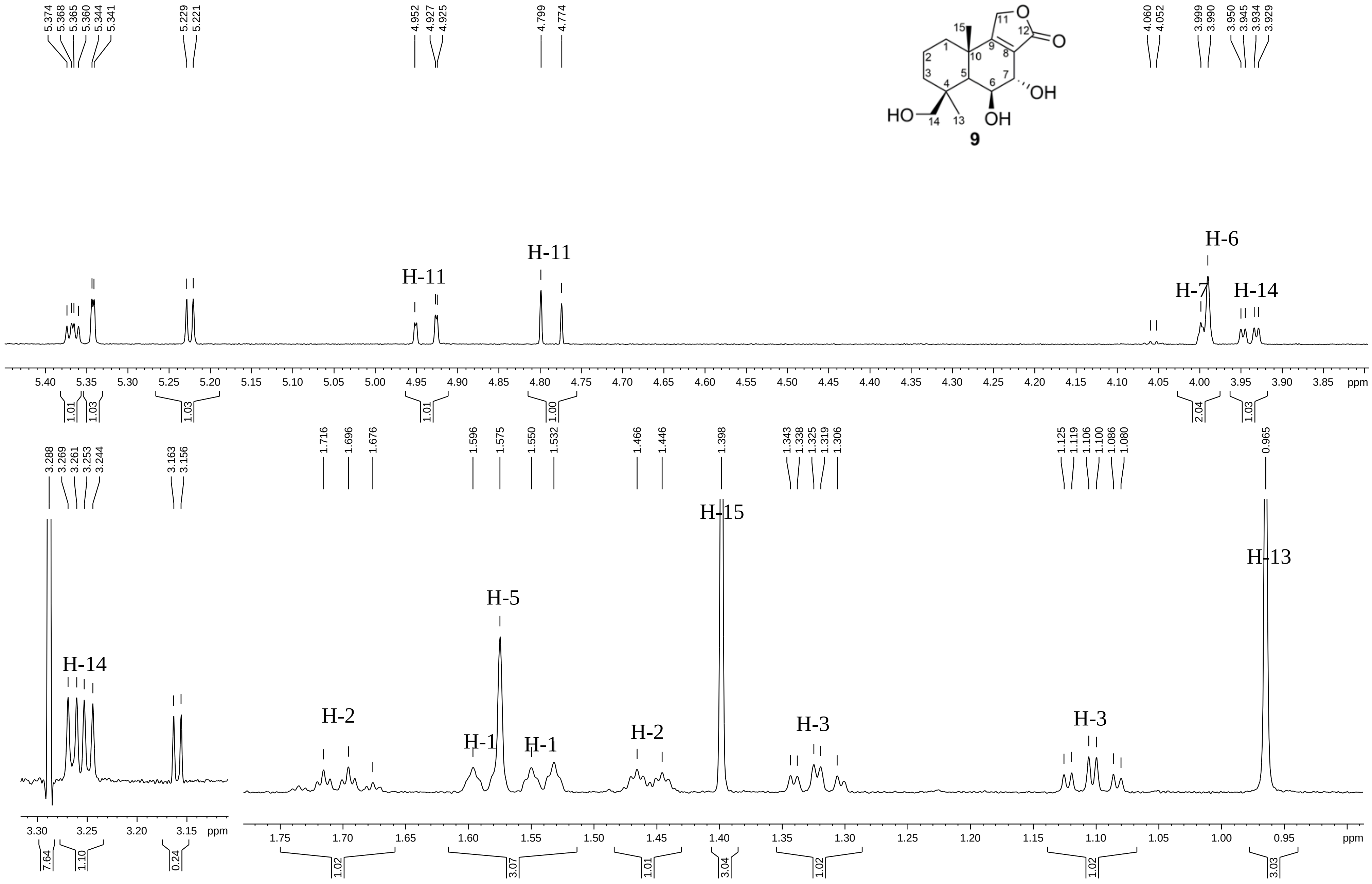

5.374 5.368 5.365 5.360 5.344 5.341
5.229 5.221
4.952 4.927 4.925
4.799 4.774
4.060 4.052
3.999 3.990
3.950 3.945 3.934 3.929
9
H-11
H-11
H-7
H-6
H-14
ppm
3.288 3.269 3.261 3.253 3.244
3.163 3.156
1.716 1.696 1.676
1.596 1.575 1.550 1.532
1.466 1.446
1.398
1.343 1.338 1.325 1.319 1.306
1.125 1.119 1.106 1.100 1.086 1.080
0.965
H-14
H-2
H-1
H-5
H-1
H-2
H-15
H-3
H-3
H-13
ppm
ppm

Figure S46. ^{13}C NMR (125 MHz, DMSO-d_6) spectrum of 6β,7α,14-trihydroxyconfertifolin (**9**)

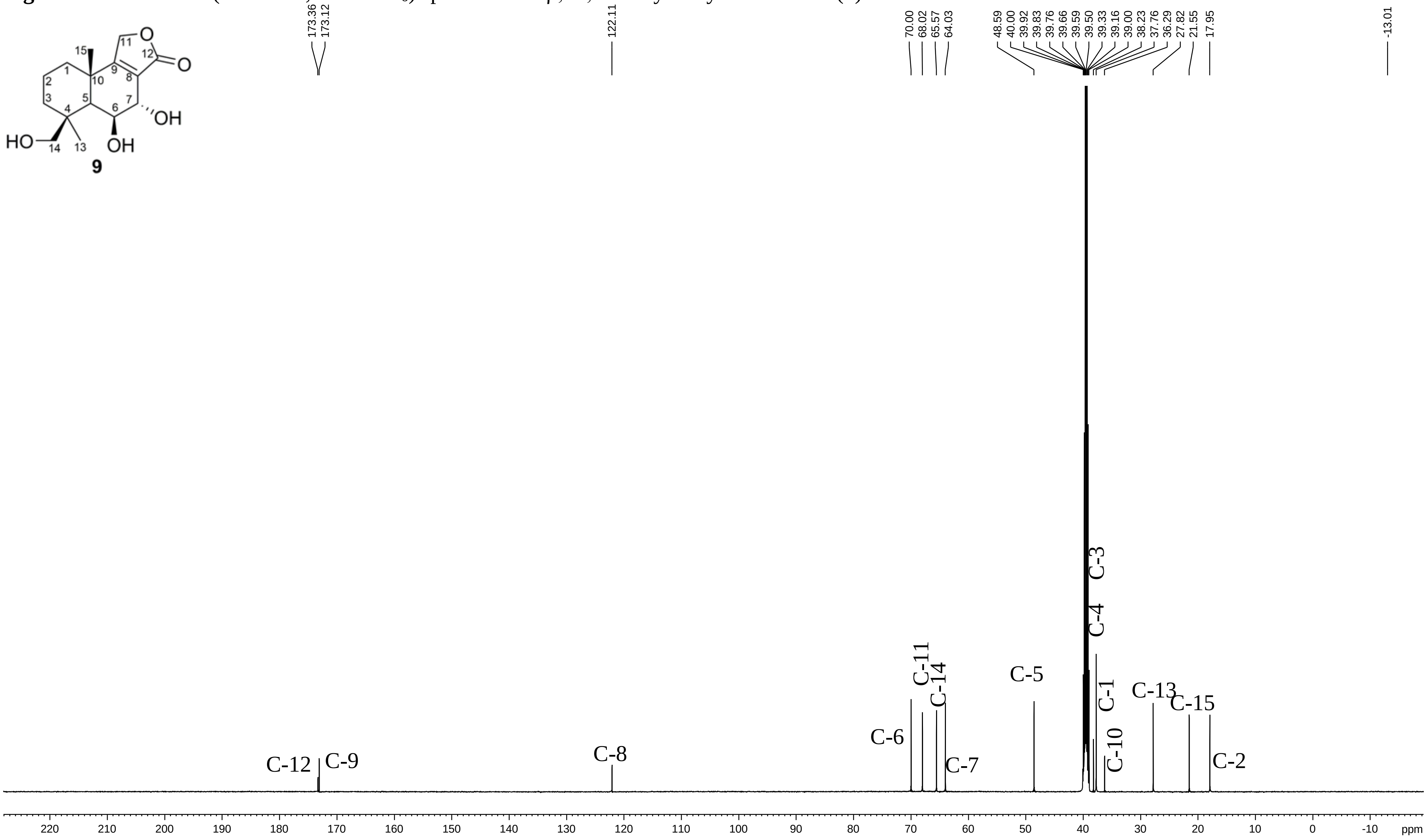

Figure S47. HSQC (700 MHz, DMSO-d$_6$) spectrum of 6β,7α,14-trihydroxyconfertifolin (**9**)

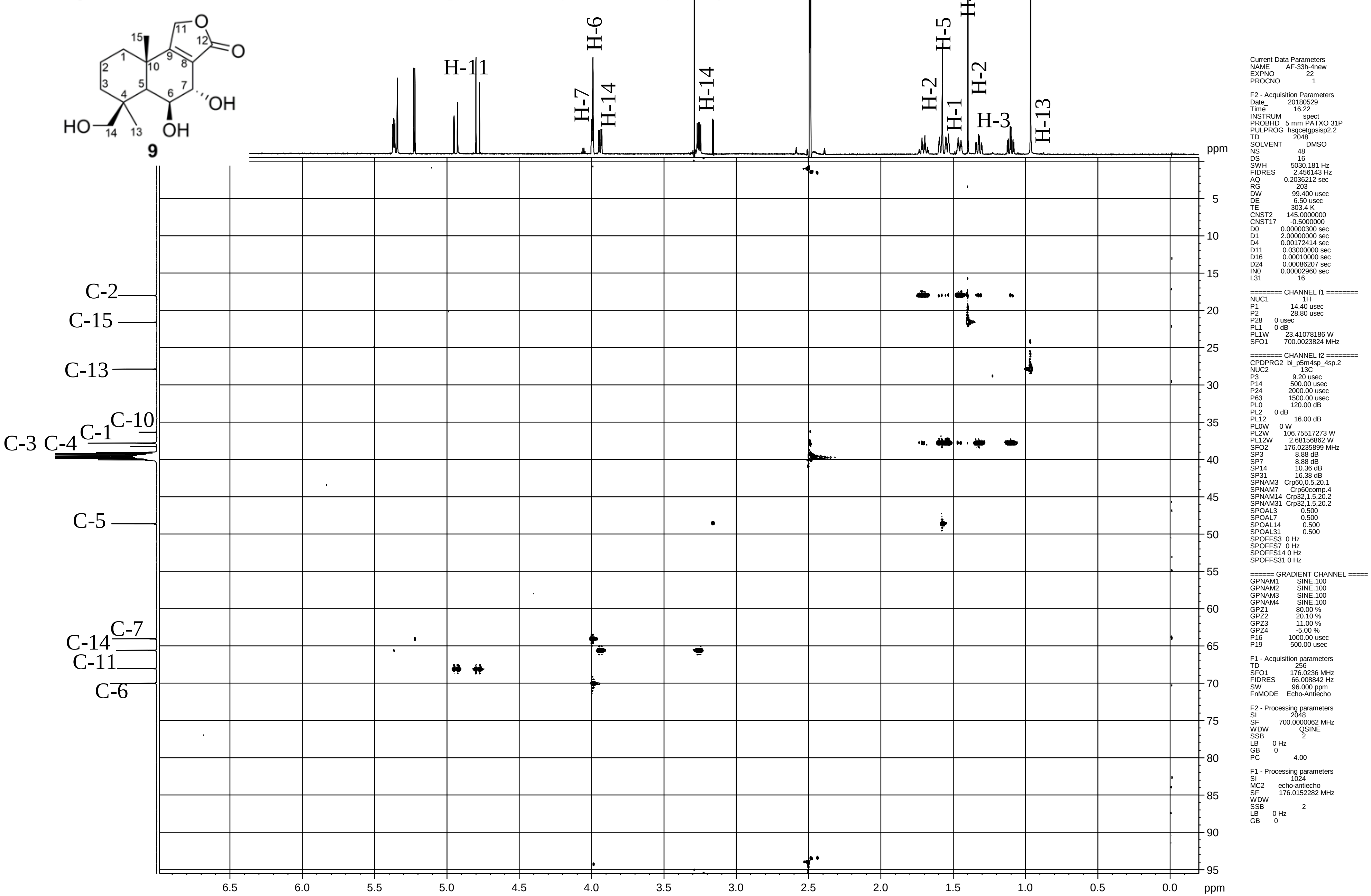

Figure S48. HMBC (500 MHz, DMSO-d_6) spectrum of 6β,7α,14-trihydroxyconfertifolin (**9**)

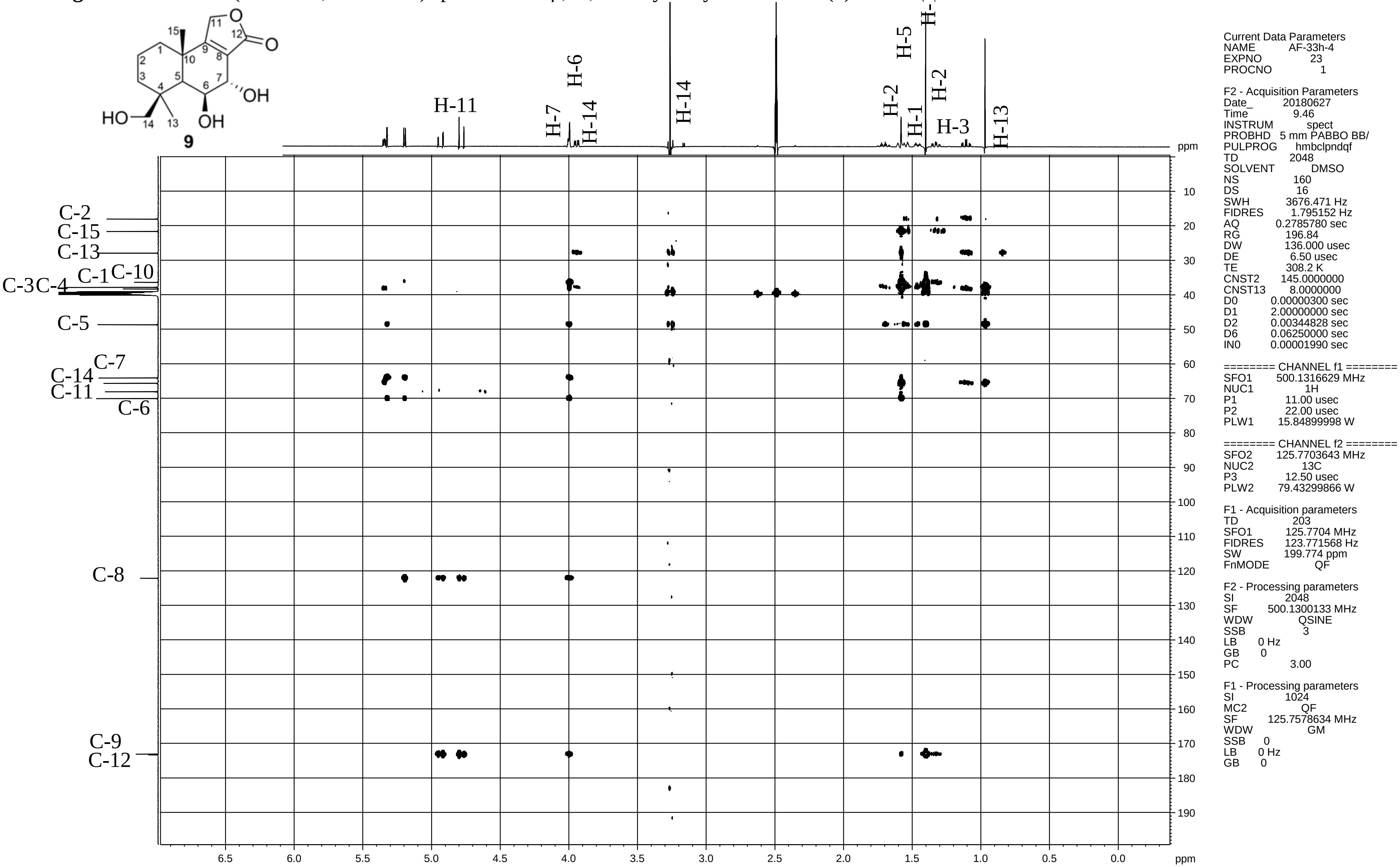

Figure S49. ROESY (700 MHz, DMSO-d_6) spectrum of 6β,7α,14-trihydroxyconfertifolin (**9**)

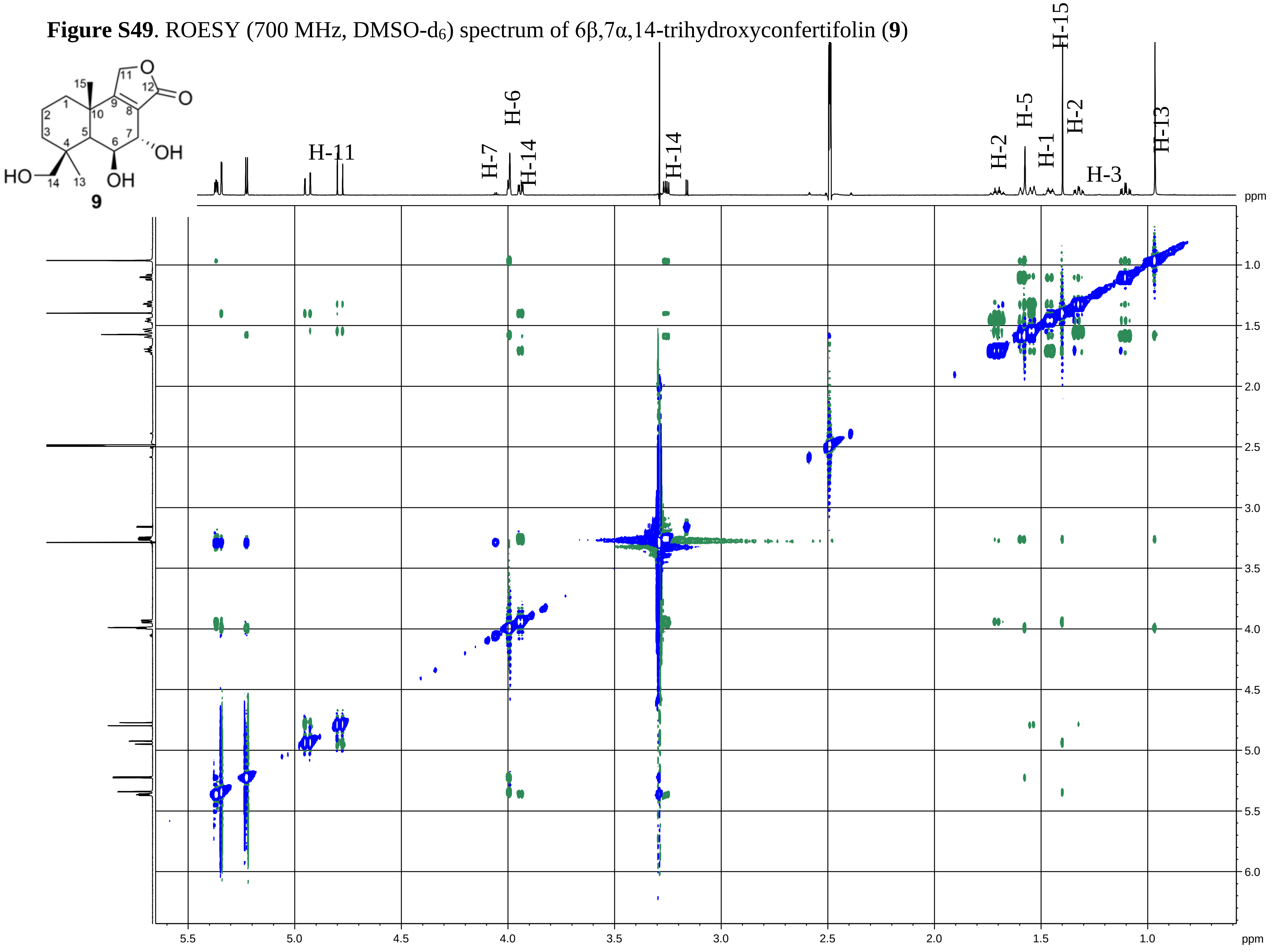

Current Data Parameters
NAME AF-33h-4new
EXPNO 23
PROCNO 1

F2 - Acquisition Parameters
Date_ 20180529
Time 23.59
INSTRUM spect
PROBHD 5 mm PATXO 31P
PULPROG roesyph.2
TD 2048
SOLVENT DMSO
NS 48
DS 16
SWH 5030.181 Hz
FIDRES 2.456143 Hz
AQ 0.2036212 sec
RG 1.12
DW 99.400 usec
DE 6.50 usec
TE 302.7 K
D0 0.00008623 sec
D1 2.00000000 sec
D12 0.00002000 sec
IN0 0.00019880 sec
L4 613
P15 270000.00 usec

======== CHANNEL f1 ========
NUC1 1H
P1 14.40 usec
P25 220.00 usec
PL1 0 dB
PL27 17.66 dB
PL1W 23.41078186 W
PL27W 0.40125081 W
SFO1 700.0023824 MHz

F1 - Acquisition parameters
TD 256
SFO1 700.0024 MHz
FIDRES 19.649145 Hz
SW 7.186 ppm
FnMODE TPPI

F2 - Processing parameters
SI 2048
SF 700.0000067 MHz
WDW SINE
SSB 2
LB 0 Hz
GB 0
PC 4.00

F1 - Processing parameters
SI 512
MC2 TPPI
SF 700.0000026 MHz
WDW
SSB 2
LB 0 Hz
GB 0

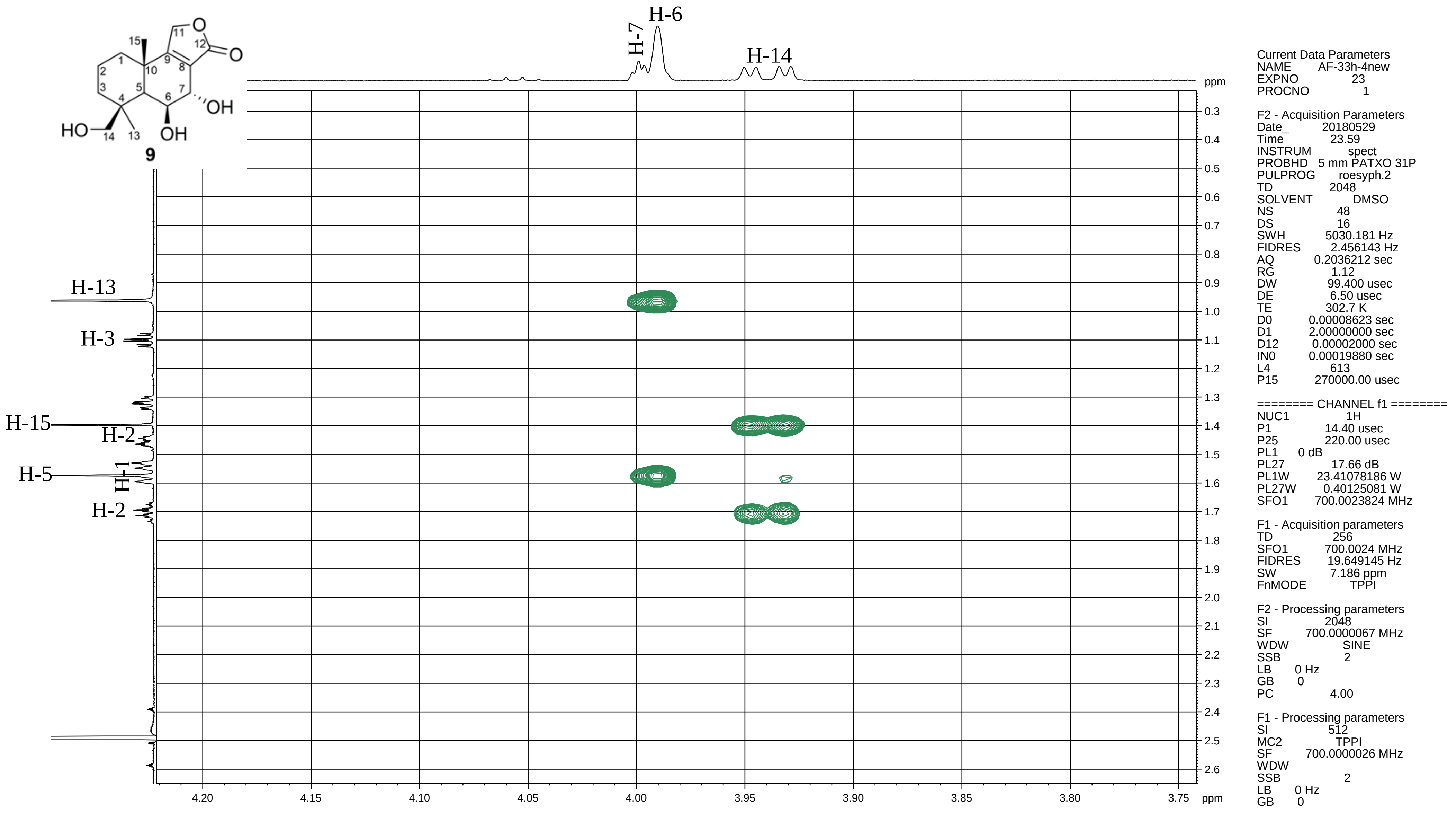
9
H-7
H-6
H-14
H-13
H-3
H-15
H-2
H-5
H-1
H-2
ppm
Current Data Parameters
NAME AF-33h-4new
EXPNO 23
PROCNO 1
F2 - Acquisition Parameters
Date_ 20180529
Time 23.59
INSTRUM spect
PROBHD 5 mm PATXO 31P
PULPROG roesyph.2
TD 2048
SOLVENT DMSO
NS 48
DS 16
SWH 5030.181 Hz
FIDRES 2.456143 Hz
AQ 0.2036212 sec
RG 1.12
DW 99.400 usec
DE 6.50 usec
TE 302.7 K
D0 0.00008623 sec
D1 2.00000000 sec
D12 0.00002000 sec
IN0 0.00019880 sec
L4 613
P15 270000.00 usec
======== CHANNEL f1 ========
NUC1 1H
P1 14.40 usec
P25 220.00 usec
PL1 0 dB
PL27 17.66 dB
PL1W 23.41078186 W
PL27W 0.40125081 W
SFO1 700.0023824 MHz
F1 - Acquisition parameters
TD 256
SFO1 700.0024 MHz
FIDRES 19.649145 Hz
SW 7.186 ppm
FnMODE TPPI
F2 - Processing parameters
SI 2048
SF 700.0000067 MHz
WDW SINE
SSB 2
LB 0 Hz
GB 0
PC 4.00
F1 - Processing parameters
SI 512
MC2 TPPI
SF 700.0000026 MHz
WDW
SSB 2
LB 0 Hz
GB 0

Figure S50. ^{1}H NMR (500 MHz, MeOD) spectrum of 6β,7α,14-trihydroxyconfertifolin (**9**)

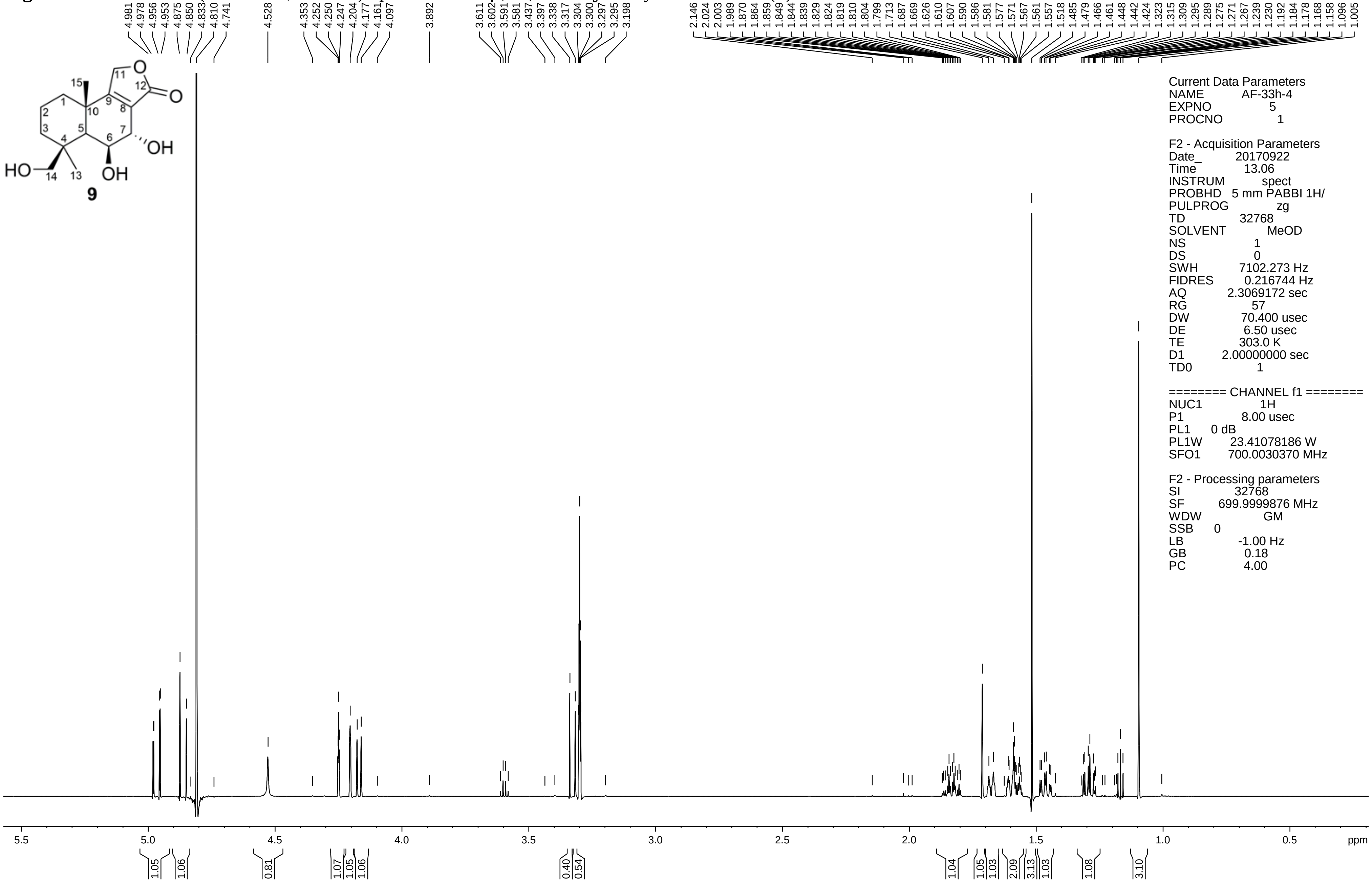

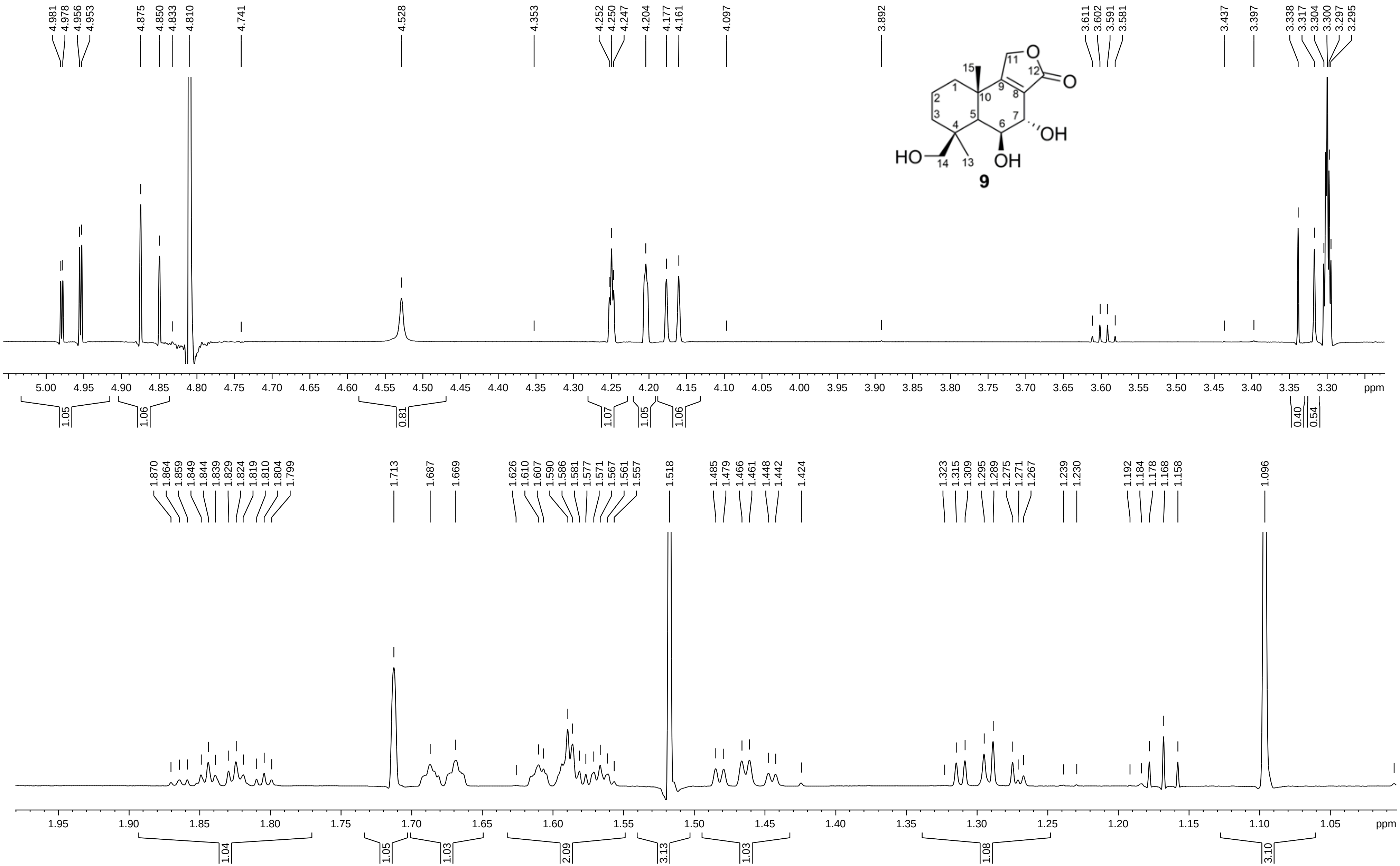
4.981 4.978 4.956 4.953 4.875 4.850 4.833 4.810 4.741 4.528 4.353 4.252 4.250 4.247 4.204 4.177 4.161 4.097 3.892 3.611 3.602 3.591 3.581 3.437 3.397 3.338 3.317 3.304 3.300 3.297 3.295
9
HO
OH
OH
ppm
1.05 1.06 0.81 1.07 1.05 1.06 0.40 0.54
1.870 1.864 1.859 1.849 1.844 1.839 1.829 1.824 1.819 1.810 1.804 1.799 1.713 1.687 1.669 1.626 1.610 1.607 1.590 1.586 1.581 1.577 1.571 1.567 1.561 1.557 1.518 1.485 1.479 1.466 1.461 1.448 1.442 1.424 1.323 1.315 1.309 1.295 1.289 1.275 1.271 1.267 1.239 1.230 1.192 1.184 1.178 1.168 1.158 1.096
ppm
1.04 1.05 1.03 2.09 3.13 1.03 1.08 3.10

Figure S51. ^{13}C NMR (125 MHz, MeOD) spectrum of 6β,7α,14-trihydroxyconfertifolin (**9**)

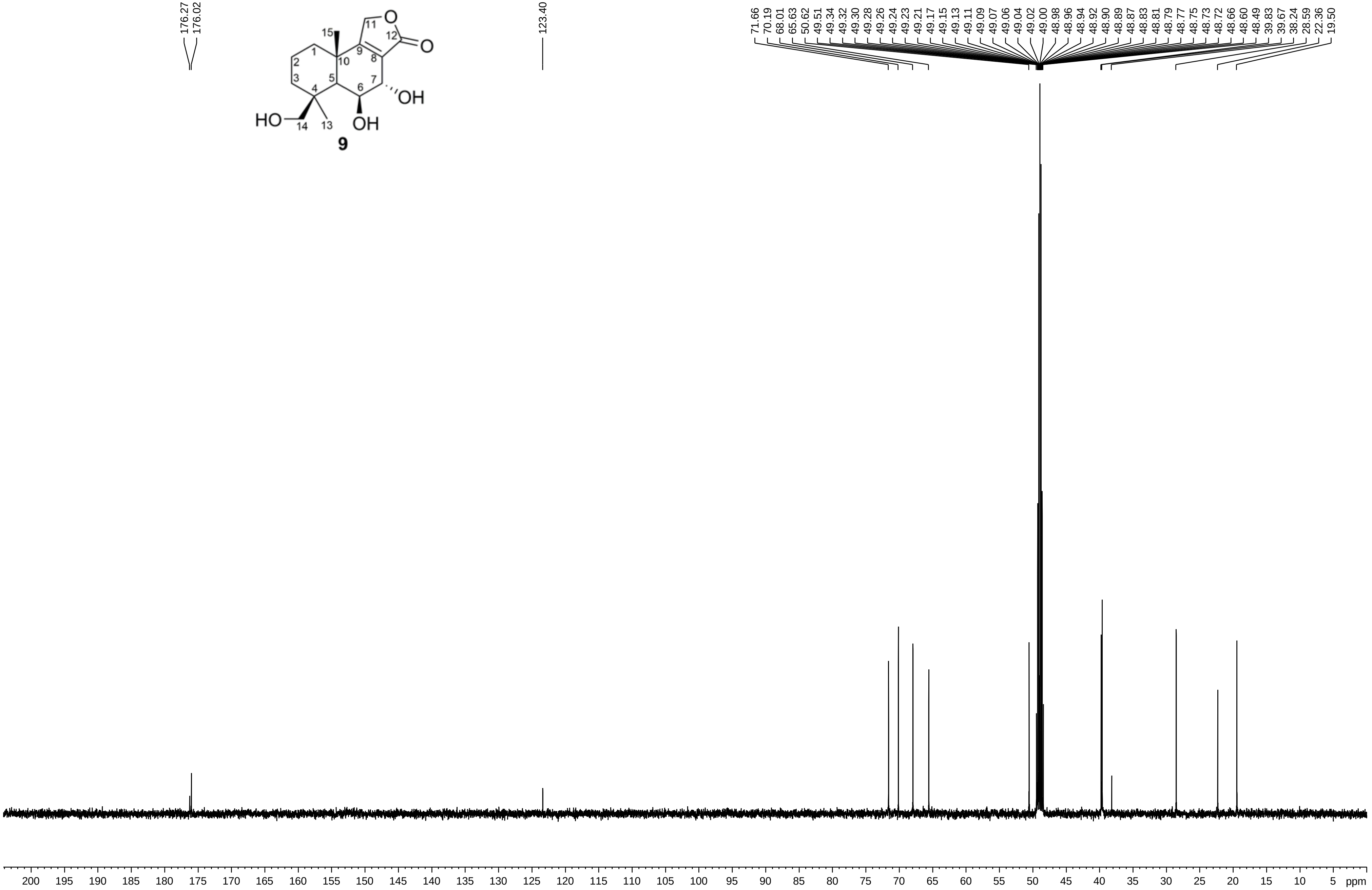

Figure S52. DEPT-135 (125 MHz, MeOD) spectrum of 6β,7α,14-trihydroxyconfertifolin (**9**)

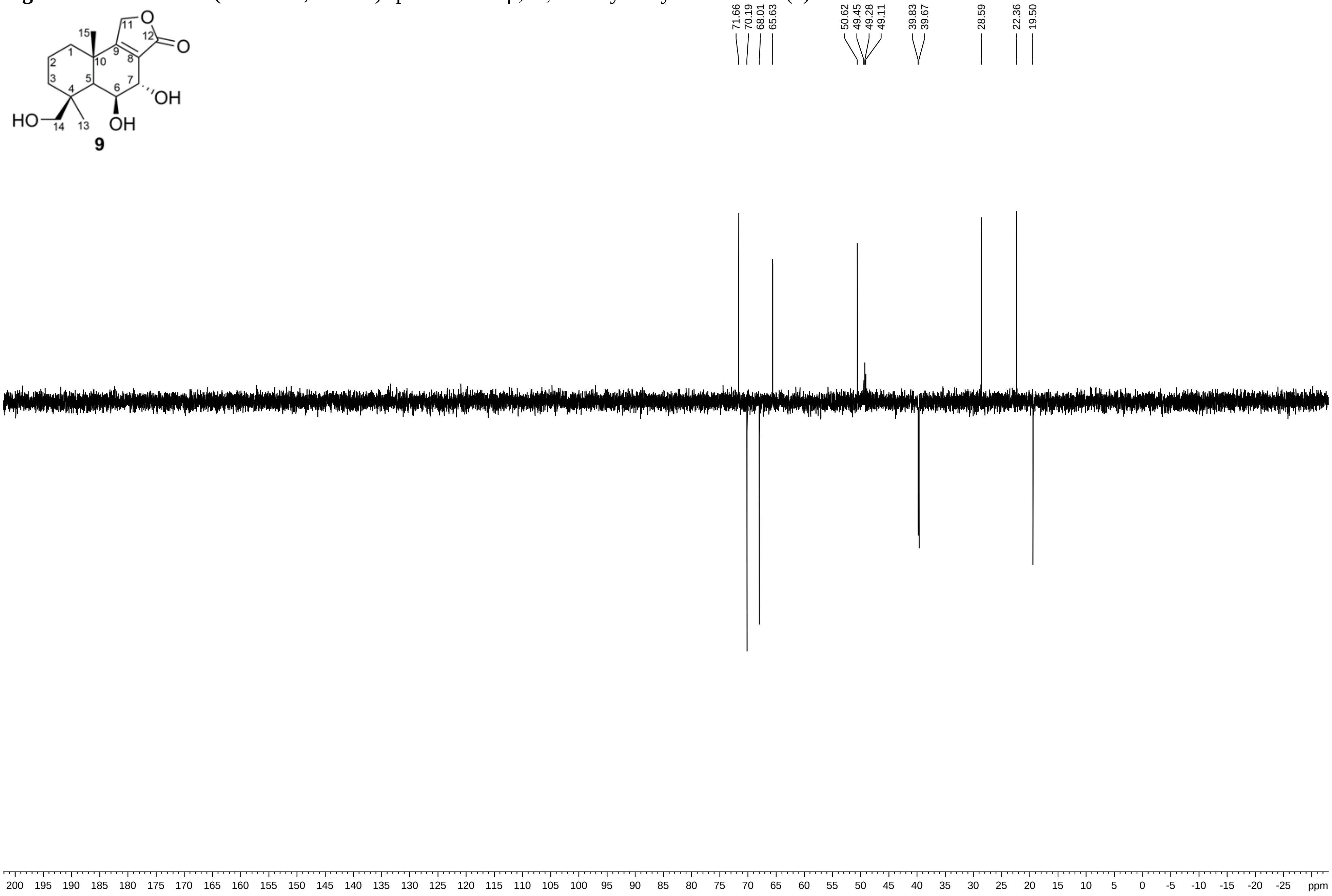

Figure S53. ^{1}H-^{1}H COSY (700 MHz, MeOD) spectrum of 6β,7α,14-trihydroxyconfertifolin (**9**)

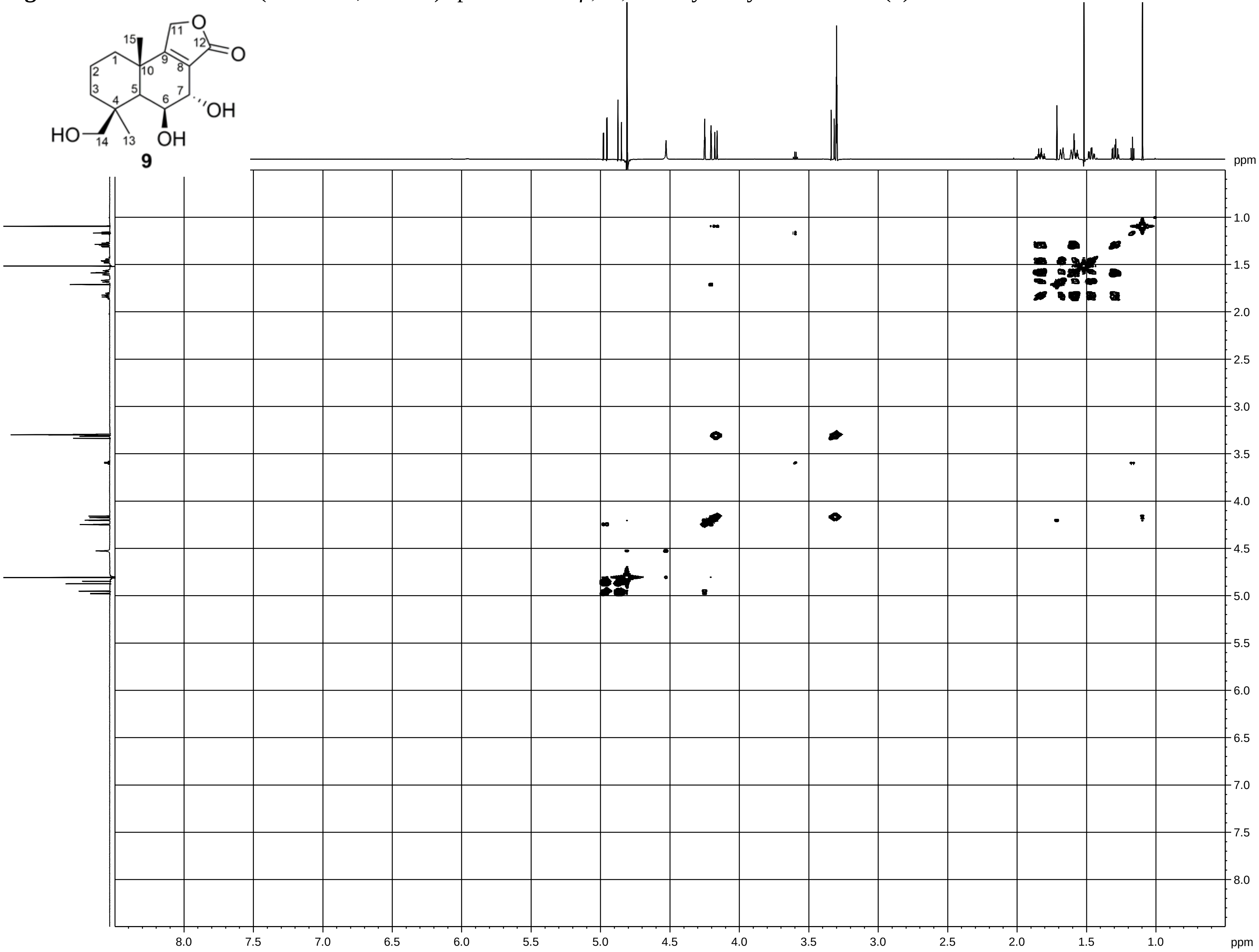

Current Data Parameters
NAME AF-33h-4
EXPNO 21
PROCNO 1

F2 - Acquisition Parameters
Date_ 20170922
Time 13.28
INSTRUM spect
PROBHD 5 mm PABBI 1H/
PULPROG cosyqf45
TD 2048
SOLVENT MeOD
NS 8
DS 16
SWH 7102.273 Hz
FIDRES 3.467907 Hz
AQ 0.1442292 sec
RG 57
DW 70.400 usec
DE 6.50 usec
TE 303.0 K
D0 0.00000300 sec
D1 2.00000000 sec
IN0 0.00014080 sec

======== CHANNEL f1 ========
NUC1 1H
P1 8.00 usec
PL1 0 dB
PL1W 23.41078186 W
SFO1 700.0030370 MHz

F1 - Acquisition parameters
TD 512
SFO1 700.003 MHz
FIDRES 13.871627 Hz
SW 10.146 ppm
FnMODE QF

F2 - Processing parameters
SI 2048
SF 699.9999876 MHz
WDW SINE
SSB 0
LB 0 Hz
GB 0
PC 4.00

F1 - Processing parameters
SI 2048
MC2 QF
SF 699.9999876 MHz
WDW GM
SSB 0
LB 0 Hz
GB 0

Figure S54. ECD spectrum of 6β,7α,14-trihydroxyconfertifolin (**9**) in methanol

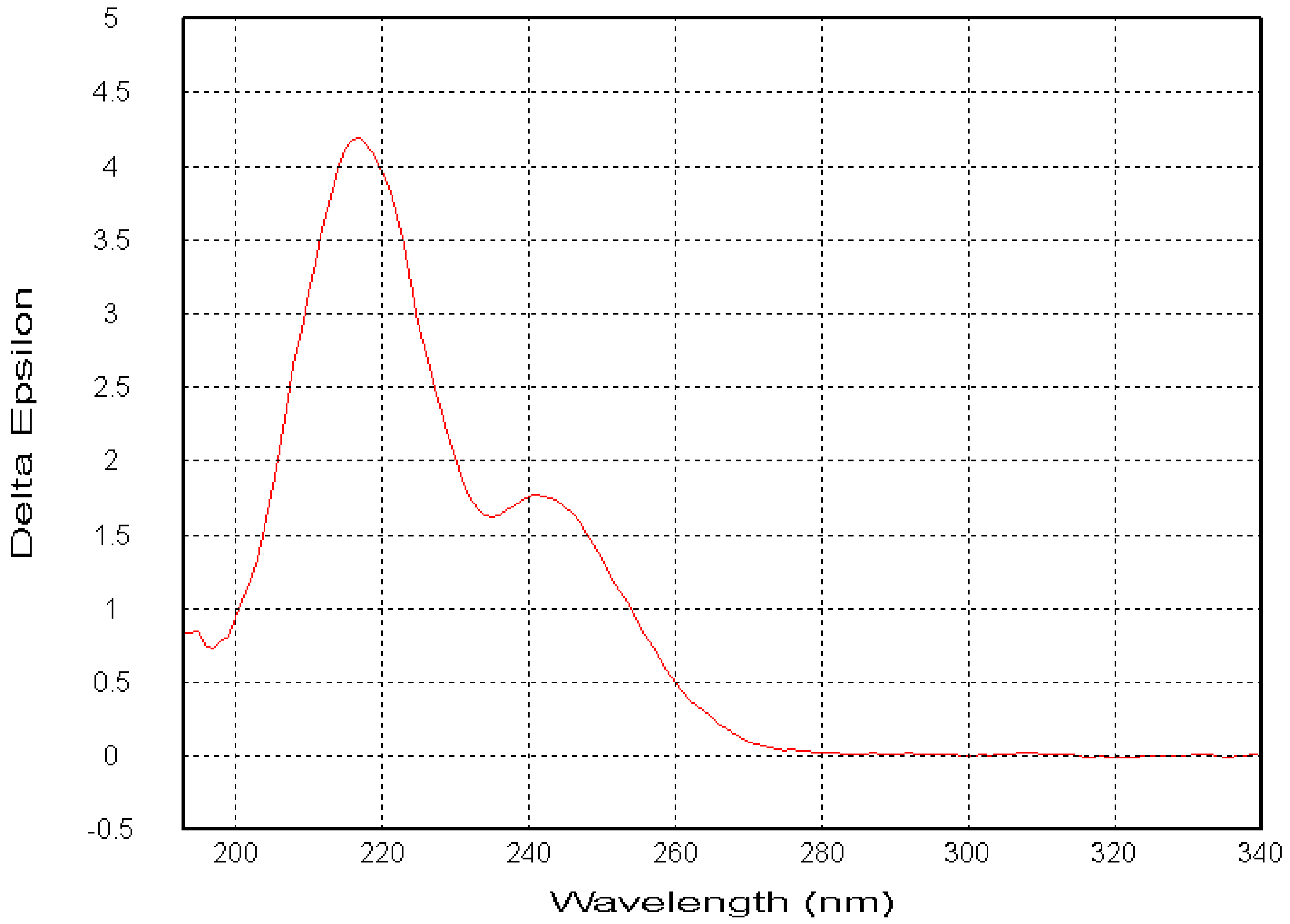

Figure S55. ^{1}H NMR (700 MHz, acetone-d_6) spectrum of 7α,14-dihydroxy-6β-p-nitrobenzoylconfertifolin (**10**)

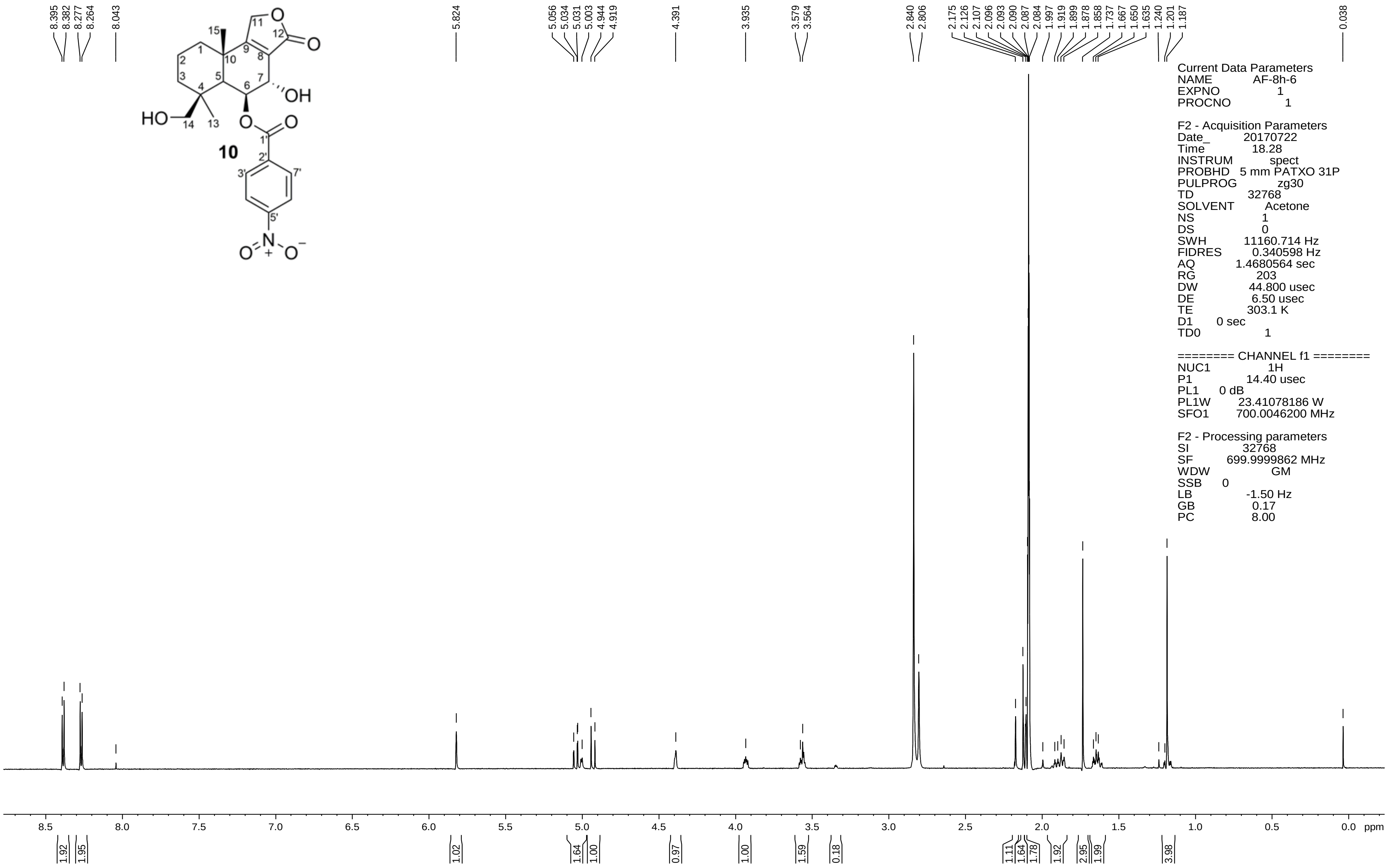

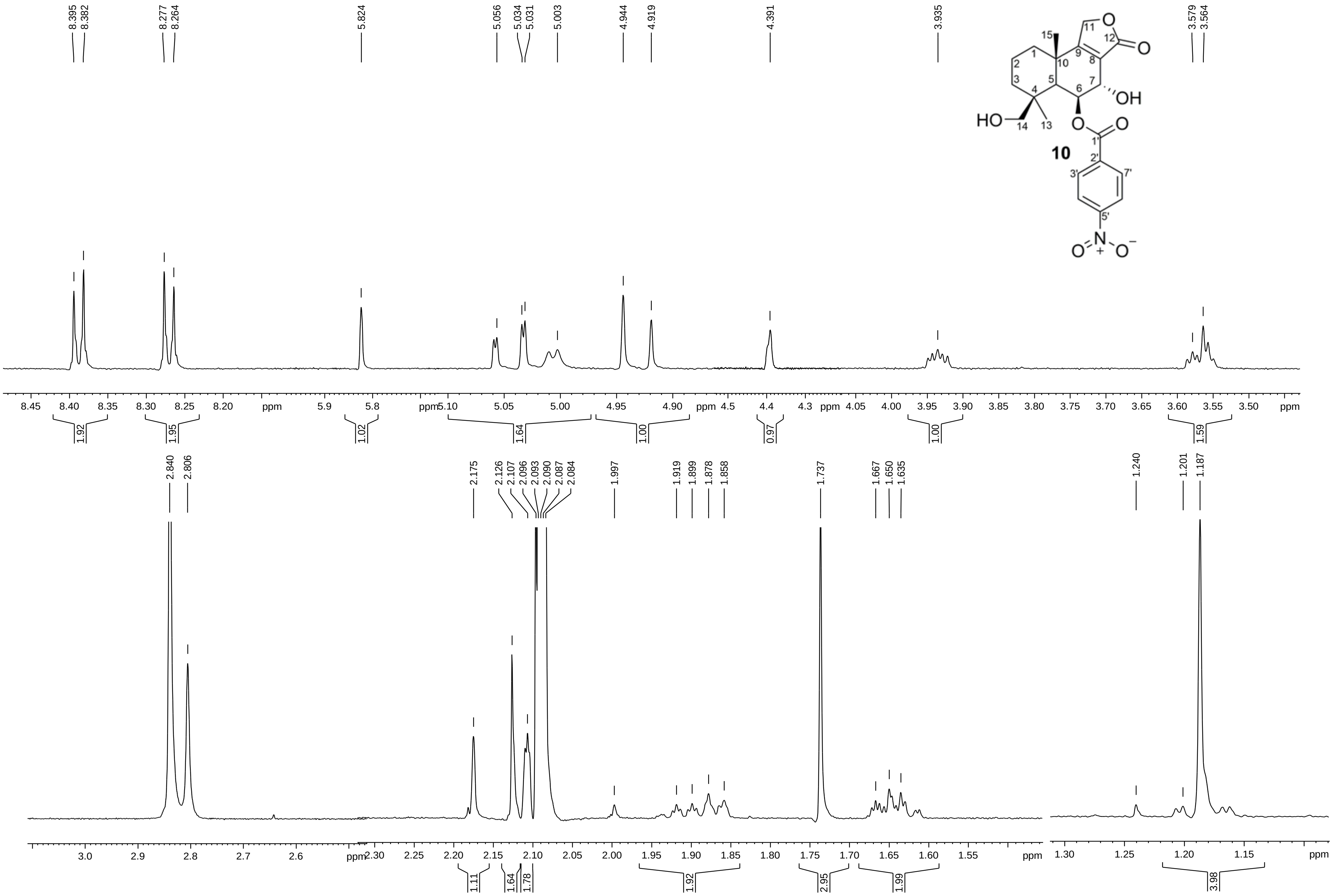
8.395
8.382
8.277
8.264
5.824
5.056
5.034
5.031
5.003
4.944
4.919
4.391
3.935
3.579
3.564
HO
OH
10
1.92
1.95
1.02
1.64
1.00
0.97
1.00
1.59
2.840
2.806
2.175
2.126
2.107
2.096
2.093
2.090
2.087
2.084
1.997
1.919
1.899
1.878
1.858
1.737
1.667
1.650
1.635
1.240
1.201
1.187
1.11
1.64
1.78
1.92
2.95
1.99
3.98
ppm

Figure S56. ^{13}C NMR (175 MHz, acetone-d$_6$) spectrum of 7α,14-dihydroxy-6β-p-nitrobenzoylconfertifolin (**10**)

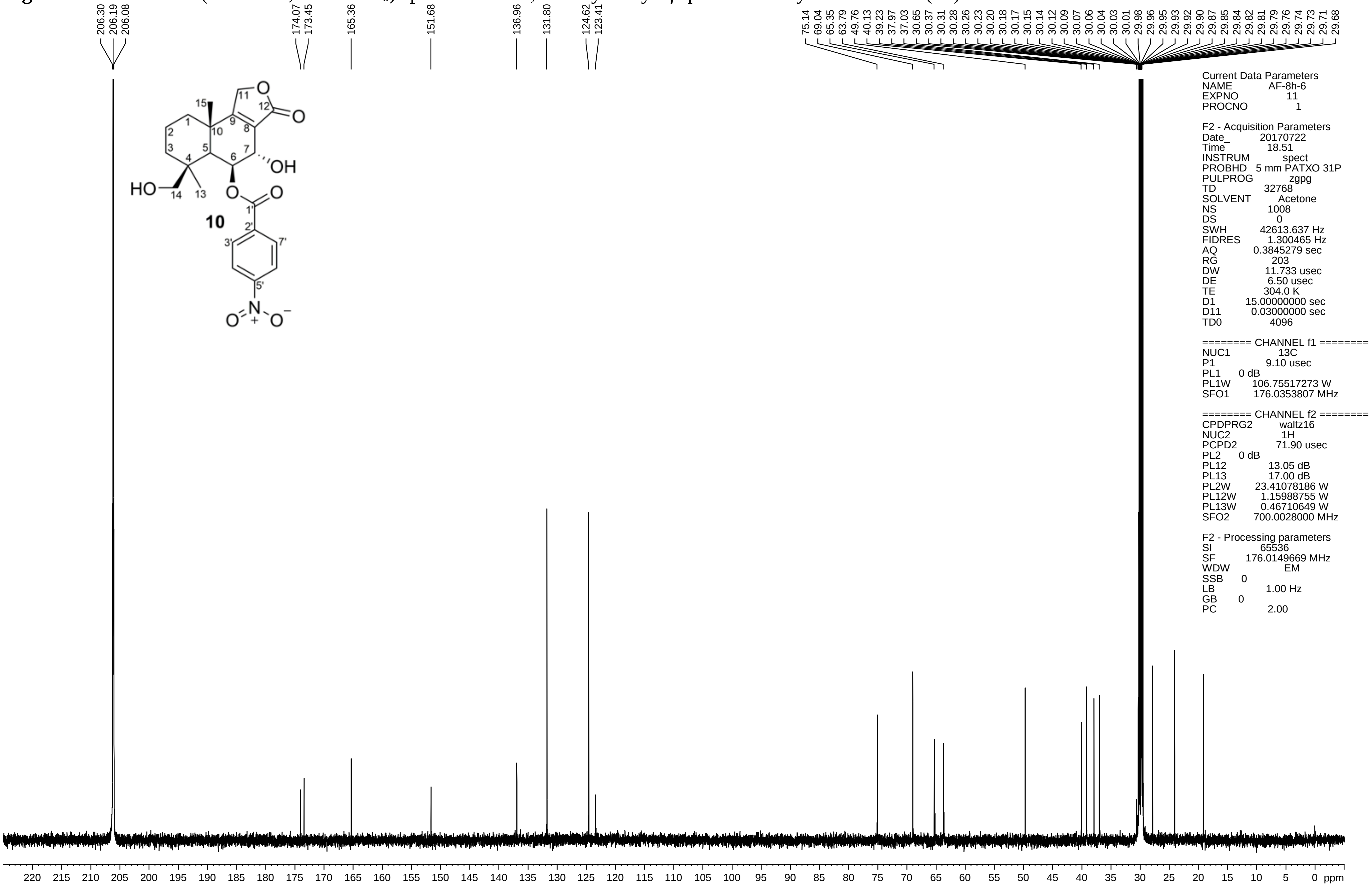

Figure S57. ECD spectrum of 7α,14-dihydroxy-6β-p-nitrobenzoylconfertifolin (**10**) in methanol

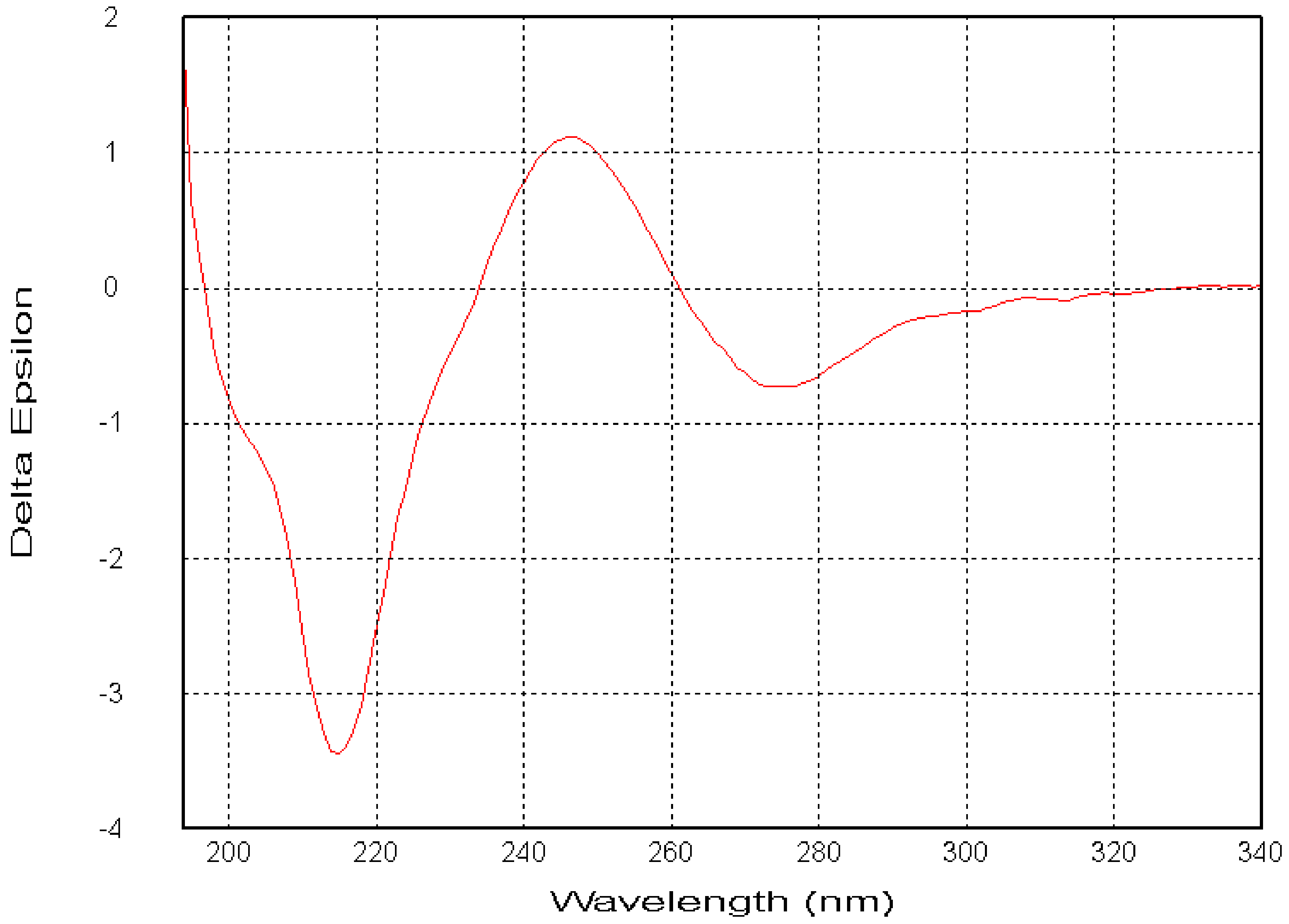

Supplement: Supplementary file 1 [file marinedrugs-17-00579-s001.pdf]
